# Global food retail environments are increasingly dominated by large chains and linked to the rising prevalence of obesity

**DOI:** 10.1038/s43016-025-01134-x

**Published:** 2025-03-03

**Authors:** Tailane Scapin, Helena Romaniuk, Alison Feeley, Karla P. Corrêa, Roland Kupka, Clara Gomez-Donoso, Liliana Orellana, Adyya Gupta, Gary Sacks, Adrian J. Cameron

**Affiliations:** 1 Global Centre for Preventive Health and Nutrition (GLOBE), Institute for Health Transformation, School of Health and Social Development, Faculty of Health, Deakin University, Geelong, Victoria Australia; 2 Biostatistics Unit, Institute for Health Transformation, School of Health and Social Development, Faculty of Health, Deakin University, Geelong, Victoria Australia; 3 UNICEF East Asia and Pacific Regional Office, Bangkok, Thailand

**Keywords:** Obesity, Risk factors

## Abstract

Retail food environments influence food purchasing and dietary patterns. A global analysis of the food retail landscape allowing comparisons across geographical regions is therefore needed to tackle diet-related non-communicable diseases. Here we examine trends in retail food environments from 2009 to 2023 across 97 countries, exploring associations with changes in obesity prevalence. Increases were observed in the density of chain outlets, grocery sales from chain retailers, unhealthy food sales per capita and digital grocery sales; non-chain outlet density and the ratio of non-chain to chain outlets declined over time. South Asia and low- and middle-income countries overall experienced the most rapid transformation. Changes in retail environments and the prevalence of obesity were found to be positively correlated. As retail environments become increasingly digital and dominated by large chains, important implications for diets and health should be expected, particularly in lower-income countries.

## Abstract (web summary)

Understanding the impact of retail food environments on human health is key to food systems transformation. This study examines global trends in retail food environments over the past two decades, showing that changes in these environments are associated with the prevalence of obesity.

## Main

Globalization, urbanization, rising incomes, market liberalization, technological advancements and customer demand have driven a large-scale transformation of food systems in most countries over the past 50 years^1–3^. This has included a shift from largely independently owned and traditional food systems to urbanized, industrialized and increasingly digitalized food systems dominated by large retail chains^4,5^. The impacts of these shifting trends have been profound, encompassing both positive outcomes, such as enhanced affordability of nutritious diets, and negative repercussions, including exacerbated inequalities in food access^6,7^. Changes in food systems have been paralleled by a notable nutrition transition, characterized by a shift from traditional diets predominantly composed of staple grains, fruits and vegetables to diets increasingly dominated by unhealthy and highly processed packaged food^8^. Typically, these foods are high in energy and nutrients of concern, with the current global epidemic of diet-related non-communicable diseases (NCDs) such as obesity, type 2 diabetes and cardiovascular diseases being the seemingly inevitable outcome^9,10^. Moreover, overconsumption of such foods considerably affects planetary health^11,12^.

Within the broad food system, retail food environments encompass the various settings where food and beverages are sourced and purchased by individuals, such as supermarkets, convenience stores, vending machines, cafes and restaurants—including their online interfaces^13,14^. Retail food environments can be assessed at both a macro level (for example, the spatial distribution of different retail types) and a micro level (for example, the availability, nutritional quality and marketing strategies within outlets)^15,16^. Both levels influence food purchases, dietary intake and, ultimately, health outcomes^17,18^.

Ownership and management structures of retail food outlets can have important implications for public health. Large retail chains, with their extensive supply chains and notable market power, influence food access, dietary patterns and health at local, national and even global scales^19^. The close collaboration between chained retailers and multinational food manufacturers often results in the extensive and uniform promotion of highly processed, unhealthy foods^20^. Monitoring the proliferation of chained retail outlets is therefore important to provide an indication of their potential influence on diets at a population level. It is also important to assess changes in the density of non-chained outlets. Although they also often sell unhealthy foods, in some low- and middle-income country settings and in underserved areas in higher-income countries, independent outlets can sometimes be the only source of food in a community and play a substantial role in ensuring food access^21–23^.

With the World Health Organization recognizing the urgent need for transformation of food systems to tackle the pandemic of diet-related NCDs^24^, it is important to understand how food systems and retail food environments are changing and whether they are indeed becoming healthier. Few studies have assessed the changes in the density of retail food environments within and between countries^25,26^, with none offering a global perspective of the food retail landscape or allowing comparisons across geographical regions or country income groups.

The aim of this study was to describe the changes in trends in (1) physical retail food environment indicators across 97 countries over a 15-year period (2009–2023), overall and by geographic region and country income status, and (2) a digital retail food environment indicator across 27 countries over a 10-year period (2014–2023) and to estimate the association between changes over time in both retail food environments and the prevalence of obesity.

## Results

### Changes in the physical retail food environment from 2009 to 2023

**Fig. 1:**
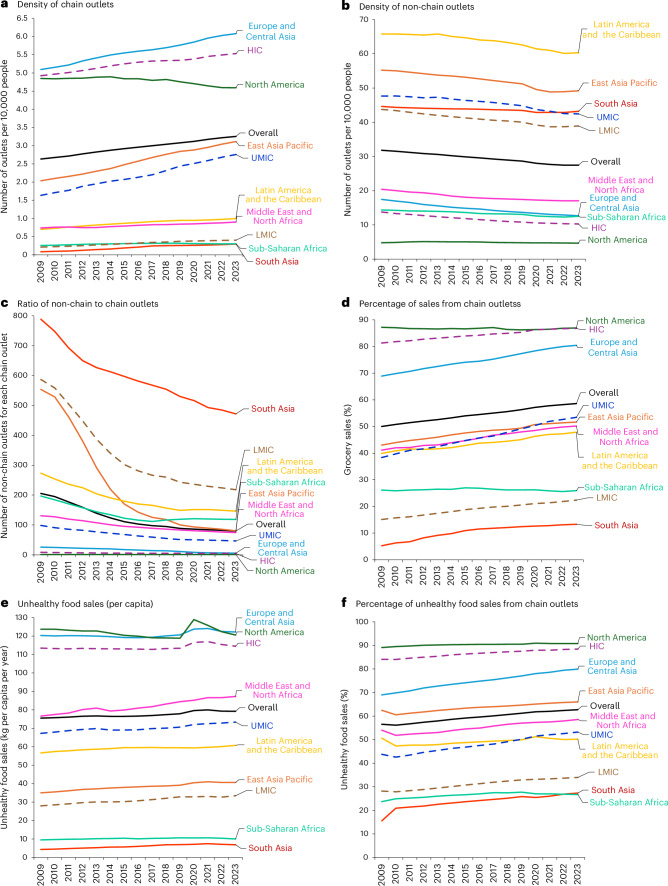
Trends over time in physical retail food environment indicators (2009–2023) overall and by region and country income group. **a**–**f**, Mean estimates of the physical retail food environment indicators: density of chain outlets (**a**), density of non-chain outlets (**b**), ratio of non-chain to chain outlets (**c**), percentage of sales from chain outlets (**d**), unhealthy food sales (per capita) (**e**) and percentage of unhealthy food sales from chain outlets (**f**); *n* = 97 countries. The lines show how each indicator changes over time. Original units and descriptions of each calculated indicator are reported in Supplementary Table 3.

**Fig. 2:**
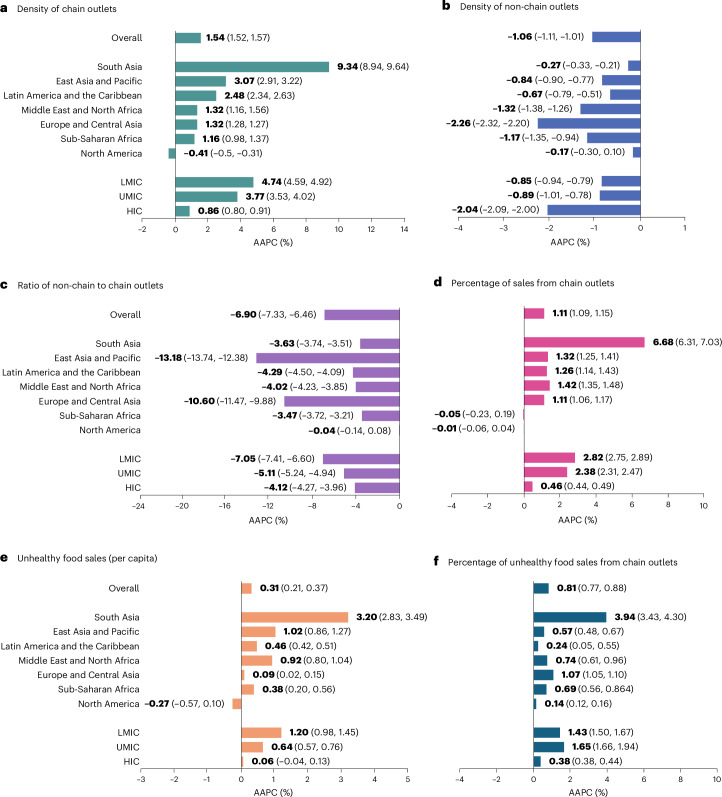
AAPC for retail food environment indicators overall and by region and country income group. **a**–**f**, AAPC and 95% CI of physical retail food environment indicators: density of chain outlets (**a**), density of non-chain outlets (**b**), ratio of non-chain to chain outlets (**c**), percentage of sales from chain outlets (**d**), unhealthy food sales (per capita) (**e**) and percentage of unhealthy food sales from chain outlets (**f**) are shown overall and by geographic region and country income status. AAPC was estimated from a joinpoint regression model. The analysis used data from 2009 to 2023 from 97 countries. Original units and descriptions of each calculated indicator are reported in Supplementary Table 3. The AAPC serves as an overall measure of the trend observed across the target period of time; it is a weighted average of the APCs identified for each segment. The AAPC values are a summary measure of the annual change in the food indicators over the study period (increase, decrease or no change).

Overall, the density of chain outlets increased from 2.63 to 3.25 outlets per 10,000 population between 2009 and 2023 (Fig. 1a), which represents a 23.6% increase over the period (Table 1). Joinpoint analysis estimated an average annual percentage change (AAPC) of 1.54% per year (95% confidence interval (CI): 1.52%, 1.57%; *P* < 0.001; Fig. 2a), with two trends estimated during the study period. Trends differed before and after 2014, with the annual percentage change (APC) slowing from 1.82% per year (95% CI: 1.72%, 1.95%; *P* < 0.001) to 1.39% per year (95% CI: 1.34%, 1.43%; *P* < 0.001; Table 1).

**Table 1:** Joinpoint regressions for the physical retail food environment indicators, overall and by regions

	Metric	Change (%)	Segment 1			Segment 2			Segment 3		
	2023	2009–2023	Years	APC (%) (95% CI)	*P* value	Years	APC (%) (95% CI)	*P* value	Years	APC (%) (95% CI)	*P* value
**Density of chain outlets**											
Overall (*n* = 97)	3.25	23.6	2009–2014	1.82 (1.72, 1.95)	<0.001	2014–2023	1.39 (1.34, 1.43)	<0.001	–	–	–
East Asia and Pacific (*n* = 14)	3.11	52.5	2009–2013	3.06 (2.45, 3.33)	<0.001	2013–2018	3.84 (3.59, 4.11)	<0.001	2018–2023	2.31 (2.04, 2.54)	<0.001
South Asia (*n* = 4)	0.30	275.0	2009–2017	14.39 (13.67, 15.02)	<0.001	2017–2023	2.96 (1.9, 4.35)	<0.001	–	–	–
Europe and Central Asia (*n* = 38)	6.08	19.4	2009–2014	1.59 (1.47, 1.81)	<0.001	2014–2018	0.89 (0.77, 1.09)	<0.001	2018–2023	1.39 (1.27, 1.72)	<0.001
Middle East and North Africa (*n* = 13)	0.90	21.6	2009–2012	0.19 (−1.26, 1.4)	0.758	2012–2023	1.63 (1.4, 2.39)	0.004	–	–	–
Sub-Saharan Africa (*n* = 10)	0.31	19.2	2009–2012	3.99 (2.89, 5.94)	<0.001	2012–2017	2.13 (−0.58, 2.62)	0.073	2017–2023	−1.02 (−1.65, −0.52)	0.024
Latin America and the Caribbean (*n* = 16)	1.00	42.9	2009–2013	3.96 (3.47, 5.15)	<0.001	2013–2017	2.75 (1.33, 3.3)	<0.001	2017–2023	1.33 (0.52, 1.74)	0.026
North America (*n* = 2)	4.59	−5.4	2009–2014	0.16 (−0.2, 1.03)	0.154	2014–2018	−0.41 (−1.02, 0.19)	0.101	2018–2023	−0.98 (−1.73, −0.64)	0.001
**Density of non-chain outlets**											
Overall (*n* = 97)	27.47	−13.7	2009–2017	−1.02 (−1.08, −0.87)	<0.001	2017–2021	−1.43 (−1.58, −1.24)	<0.001	2021–2023	−0.46 (−0.92, −0.11)	0.012
East Asia and Pacific (*n* = 14)	49.19	−10.9	2009–2017	−0.67 (−0.76, −0.5)	<0.001	2017–2021	−1.7 (−1.89, −1.42)	<0.001	2021–2023	0.19 (−0.47, 0.75)	0.469
South Asia (*n* = 4)	43.20	−3.2	2009–2023	−0.27 (−0.33, −0.21)	<0.001	–	–	–	–	–	–
Europe and Central Asia (*n* = 38)	12.68	−27.4	2009–2013	−2.9 (−3.47, −2.65)	<0.001	2013–2021	−2.13 (−2.45, −1.99)	<0.001	2021–2023	−1.47 (−2.08, −1.08)	<0.001
Middle East and North Africa (*n* = 13)	17.07	−16.4	2009–2016	−1.96 (−2.14, −1.82)	<0.001	2016–2023	−0.67 (−0.82, −0.49)	<0.001	–	–	–
Sub-Saharan Africa (*n* = 10)	12.50	−13.0	2009–2023	−1.17 (−1.35, −0.94)	<0.001	–	–	–	–	–	–
Latin America and the Caribbean (*n* = 16)	60.32	−8.3	2009–2014	−0.13 (−0.49, 0.83)	0.478	2014–2023	−0.96 (−1.25, −0.81)	<0.001	–	–	–
North America (*n* = 2)	4.69	−1.9	2009–2012	2.6 (1.36, 4.42)	<0.001	2012–2023	−0.91 (−1.06, −0.77)	<0.001	–	–	–
**Ratio of non-chain to chain outlets**											
Overall (*n* = 97)	79.61	−61.3	2009–2016	−10.12 (−11.53, −9.05)	<0.001	2016–2023	−3.58 (−4.66, −1.99)	0.001	–	–	–
East Asia and Pacific (*n* = 14)	80.65	−85.4	2009–2011	−7.95 (−14.36, −1.08)	0.024	2011–2016	−22.24 (−24.67, −20.65)	<0.001	2016–2023	−7.63 (−8.81, −6.08)	<0.001
South Asia (*n* = 4)	471.64	−40.2	2009–2012	−6.29 (−7.57, −5.43)	<0.001	2012–2023	−2.89 (−3.03, −2.75)	<0.001	–	–	–
Europe and Central Asia (*n* = 38)	5.56	−78.4	2009–2017	−6.51 (−11.24, −1.1)	0.042	2017–2021	−18.41 (−20.54, −3.54)	<0.001	2021–2023	−10.3 (−16.98, −5.12)	<0.001
Middle East and North Africa (*n* = 13)	74.50	−43.0	2009–2011	−4.11 (−5.5, −2.69)	<0.001	2011–2015	−5.9 (−6.29, −2.65)	<0.001	2015–2023	−3.05 (−4.14, −2.36)	<0.001
Sub-Saharan Africa (*n* = 10)	118.61	−39.7	2009–2016	−7.56 (−8.08, −6.99)	<0.001	2016–2023	0.8 (0.21, 1.34)	0.013	–	–	–
Latin America and the Caribbean (*n* = 16)	146.18	−46.5	2009–2015	−6.86 (−8.15, −6.36)	<0.001	2015–2019	−4.12 (−5.86, −2.37)	<0.001	2019–2023	−0.46 (−1.43, 1.72)	0.402
North America (*n* = 2)	1.07	−1.8	2009–2012	1.12 (0.42, 2.28)	<0.001	2012–2018	−0.94 (−1.32, −0.7)	<0.001	2018–2023	0.35 (0.09, 0.75)	0.007
**Percentage of grocery sales (chain outlets)**											
Overall (*n* = 97)	58.60	17.2	2009–2021	1.17 (1.15, 1.21)	<0.001	2021–2023	0.76 (0.57, 1.03)	<0.001	–	–	–
East Asia and Pacific (*n* = 14)	51.65	20.1	2009–2015	1.63 (1.41, 2.14)	<0.001	2015–2023	1.10 (0.82, 1.22)	<0.001	–	–	–
South Asia (*n* = 4)	13.31	153.0	2009–2015	13.08 (11.81, 14.27)	<0.001	2015–2023	2.12 (1.37, 2.87)	<0.001	–	–	–
Europe and Central Asia (*n* = 38)	80.44	16.7	2009–2023	1.11 (1.06, 1.17)	<0.001	–	–	–	–	–	–
Middle East and North Africa (*n* = 13)	50.16	21.9	2009–2013	1.06 (0.39, 1.33)	0.002	2013–2017	2.0 (1.71, 2.2)	<0.001	2017–2023	1.28 (1.03, 1.4)	<0.001
Sub-Saharan Africa (*n* = 10)	25.91	−0.8	2009–2015	0.6 (0.19, 1.71)	0.008	2015–2023	−0.53 (−1.06, −0.28)	<0.001	–	–	–
Latin America and the Caribbean (*n* = 16)	47.87	19.9	2009–2014	0.94 (−0.08, 1.93)	0.065	2014–2023	1.44 (0.37, 2.24)	0.012	–	–	–
North America (*n* = 2)	86.97	−0.3	2009–2020	−0.07 (−0.32, 0.1)	0.067	2020–2023	0.22 (−0.05, 0.6)	0.150	–	–	–
**Unhealthy food sales per capita**											
Overall (*n* = 97)	79.23	4.9	2009–2017	0.17 (−0.13, 0.29)	0.152	2017–2021	0.98 (0.63, 1.19)	0.006	2021–2023	−0.49 (−1.17, 0.17)	0.144
East Asia and Pacific (*n* = 14)	40.61	16.0	2009–2021	1.2 (0.93, 2.5)	0.001	2021–2023	−0.06 (−1.34, 1.2)	0.763	–	–	–
South Asia (*n* = 4)	6.90	58.6	2009–2021	4.61 (4.31, 4.99)	<0.001	2021–2023	−4.84 (−7.55, −1.24)	0.007	–	–	–
Europe and Central Asia (*n* = 38)	122.14	1.5	2009–2017	−0.15 (−0.3, −0.05)	0.002	2017–2021	1.03 (0.75, 1.22)	<0.001	2021–2023	−0.84 (−1.34, −0.3)	0.003
Middle East and North Africa (*n* = 13)	87.24	13.9	2009–2023	0.92 (0.8, 1.04)	<0.001	–	–	–	–	–	–
Sub-Saharan Africa (*n* = 10)	10.02	5.1	2009–2013	1.59 (1.06, 3.09)	<0.001	2013–2021	0.6 (0.23, 0.86)	0.008	2021–2023	−2.84 (−4.11, −1.22)	<0.001
Latin America and the Caribbean (*n* = 16)	60.74	7.2	2009–2015	0.8 (0.68, 0.92)	<0.001	2015–2020	−0.08 (−0.27, 0.06)	0.153	2020–2023	0.72 (0.44, 1.17)	<0.001
North America (*n* = 2)	120.55	−2.5	2009–2017	−0.6 (−1.34, 0.35)	0.067	2017–2021	1.45 (−0.94, 2.12)	0.132	2021–2023	−2.32 (−4.52, 0.79)	0.144
**Percentage of unhealthy food sales (chain outlets)**											
Overall (*n* = 97)	62.7	10.9	2009–2020	0.89 (0.84, 1.09)	<0.001	2020–2023	0.51 (0.31, 0.81)	<0.001	–	–	–
East Asia and Pacific (*n* = 14)	66.0	5.7	2009–2023	0.57 (0.48, 0.67)	<0.001	–	–	–	–	–	–
South Asia (*n* = 4)	27.3	75.7	2009–2012	12.84 (8.87, 14.8)	<0.001	2012–2023	1.63 (0.67, 2.31)	0.006	–	–	–
Europe and Central Asia (*n* = 38)	79.9	15.8	2009–2013	1.34 (1.21, 1.54)	<0.001	2013–2023	0.97 (0.93, 1)	<0.001	–	–	–
Middle East and North Africa (*n* = 13)	58.5	8.4	2009–2012	−0.26 (−0.9, 0.84)	0.841	2012–2023	1.01 (0.81, 1.62)	0.001	–	–	–
Sub-Saharan Africa (*n* = 10)	26.7	12.8	2009–2017	1.65 (1.36, 1.99)	<0.001	2017–2023	−0.56 (−1.01, −0.19)	0.006	–	–	–
Latin America and the Caribbean (*n* = 16)	50.2	−0.8	2009–2012	−1.12 (−2.1, 0.3)	0.209	2012–2023	0.62 (0.39, 1.43)	0.012	–	–	–
North America (*n* = 2)	90.8	1.9	2009–2012	0.38 (0.26, 0.47)	<0.001	2012–2023	0.08 (0.05, 0.10)	0.003	–	–	–

APC and 95% CI estimated using joinpoint regression models are shown. Original units and descriptions of each calculated indicator are reported in Supplementary Table 3. The results represent the APC in each indicator for each identified segment over the analysis period (2009–2023), overall and by region. The sample size (number of countries) is given for each region (first column). The percentage change was calculated as follows: (2023 crude metric − 2009 crude metric)/2009 crude metric × 100. ‘Change (%)’ indicates the increase or decrease in the 2023 metric compared with the metric in the series’ first year (2009). Two-tailed *t*-test was used to test whether APC was statistically different from zero, with no adjustment made for multiple comparisons.

The trends in each geographic region were not constant over time (Table 1), with a faster increase in the density of chain outlets observed in earlier years (before 2017 and 2018). In low- and middle-income countries (LMICs), growth was faster in earlier years, while high-income countries (HICs) experienced three distinct trends, with the slowest growth from 2016 to 2020 (Table 2). There was constant growth in the density of chain outlets during the study period in upper-middle-income countries (UMICs).

**Table 2:** Joinpoint regressions for the physical and digital retail food environment indicators, by country income group

	Metric	Change (%)	Segment 1			Segment 2			Segment 3		
	2023	2009–2023	Years	APC (%) (95% CI)	*P* value	Years	APC (%) (95% CI)	*P* value	Years	APC (%) (95% CI)	*P* value
**Density of chain outlets**											
LMICs (*n* = 29)	0.40	89.32	2009–2015	6.35 (6.1, 7.26)	<0.001	2015–2019	5.01 (4.06, 5.68)	<0.001	2019–2023	2.1 (1.18, 2.59)	<0.001
UMICs (*n* = 26)	2.76	68.67	2009–2023	3.77 (3.53, 4.02)	<0.001	–	–	–	–	–	–
HICs (*n* = 42)	5.53	12.34	2009–2016	1.09 (0.96, 1.26)	<0.001	2016–2020	0.39 (0.23, 1.06)	<0.001	2020–2023	0.98 (0.58, 1.49)	<0.001
**Density of non-chain outlets**											
LMICs (*n* = 29)	38.92	−11.06	2009–2021	−0.98 (−1.12, −0.92)	<0.001	2021–2023	−0.1 (−0.8, 0.35)	0.49	–	–	–
UMICs (*n* = 26)	42.46	−10.91	2009–2016	−0.46 (−0.67, −0.1)	0.031	2016–2023	−1.33 (−1.72, −1.11)	<0.001	–	–	–
HICs (*n* = 42)	10.29	−25.32	2009–2016	−2.48 (−2.72, −2.4)	<0.001	2016–2020	−2.1 (−2.42, −1.8)	<0.001	2020–2023	−0.93 (−1.27, −0.43)	<0.001
**Ratio of non-chain to chain outlets**											
LMICs (*n* = 29)	217.87	−62.87	2009–2011	−7.03 (−11.1, −3.34)	<0.001	2011–2015	−12.92 (−13.8, −5)	<0.001	2015–2023	−3.98 (−4.67, −2.88)	<0.001
UMICs (*n* = 26)	46.43	−52.82	2009–2015	−5.8 (−6.03, −4.68)	<0.001	2015–2019	−6.76 (−7.2, −6.26)	<0.001	2019–2023	−2.37 (−2.94, −1.32)	<0.001
HICs (*n* = 42)	4.69	−44.40	2009–2015	−6.64 (−7.3, −6.18)	<0.001	2015–2023	−2.19 (−2.5, −1.76)	<0.001	–	–	–
**Percentage of grocery sales from chain outlets**											
LMICs (*n* = 29)	22.39	65.13	2009–2016	3.57 (3.37, 3.76)	<0.001	2016–2023	2.08 (1.91, 2.24)	<0.001	–	–	–
UMICs (*n* = 26)	53.45	81.00	2009–2023	2.38 (2.31, 2.47)	<0.001	–	–	–	–	–	–
HICs (*n* = 42)	86.80	11.42	2009–2021	0.5 (0.48, 0.56)	<0.001	2021–2023	0.22 (0.06, 0.45)	0.002	–	–	–
**Unhealthy food sales per capita**											
LMICs (*n* = 29)	33.47	19.69	2009–2020	1.42 (1.21, 2.76)	<0.001	2020–2023	0.37 (−1.53, 1.3)	0.501	–	–	–
UMICs (*n* = 26)	73.35	9.08	2009–2012	1.13 (0.74, 2.21)	<0.001	2012–2016	−0.17 (−0.4, 0.24)	0.407	2016–2023	0.9 (0.73, 1.37)	0.007
HICs (*n* = 42)	114.45	0.89	2009–2017	−0.11 (−0.36, 0.02)	0.107	2017–2021	0.92 (0.57, 1.15)	0.002	2021–2023	−1.01 (−1.69, −0.26)	<0.001
**Percentage of unhealthy food sales from chain outlets**											
LMICs (*n* = 29)	33.96	20.6	2009–2012	1.16 (0.76, 1.7)	<0.001	2012–2018	2.06 (1.87, 2.57)	<0.001	2018–2023	0.85 (0.52, 1.1)	<0.001
UMICs (*n* = 26)	53.22	21.7	2009–2023	1.65 (1.53, 1.78)	<0.001	–	–	–	–	–	–
HICs (*n* = 42)	88.44	5.2	2009–2017	0.48 (0.43, 0.55)	<0.001	2017–2023	0.26 (0.18, 0.32)	<0.001	–	–	–
**Digital food sales per capita**											
Overall (*n* = 27)	116.74	325	2014–2023	20.04 (14.91, 25.87)	<0.001	–	–	–	–	–	–
LMICs (*n* = 3)	3.30	1,550	2014–2020	54.18 (47.21, 68.53)	<0.001	2020–2023	10.05 (−20.05, 24.7)	0.276	–	–	–
UMICs (*n* = 8)	29.06	665	2014–2018	10.57 (−15.47, 26.4)	0.364	2018–2023	44.15 (34.46, 69.68)	<0.001	–	–	–
HICs (*n* = 16)	181.84	310	2014–2023	19.58 (14.5, 25.36)	<0.001	–	–	–	–	–	–

APC and 95% CI estimated using joinpoint regression models are shown. Original units and descriptions of each calculated indicator are reported in Supplementary Table 3. The results represent the APC in each indicator for each identified segment over the analysis period (2009–2023) by country income group. The sample size (number of countries) is given for each income group (first column). Data for the physical retail food environment indicators included data from 97 countries from 2009 to 2023. For the digital food sales per capita indicator, overall results are also presented and data were available for 27 countries from 2014 to 2023. The percentage change was calculated as follows: (2023 crude metric − 2009 crude metric)/2009 crude metric × 100—except for the digital food sales indicators where data from 2014 rather than 2009 were used. ‘Change (%)’ indicates the increase or decrease in the 2023 metric compared with the metric in the series’ first year (2009 or 2014 for the digital food sales indicator). Two-tailed *t*-test was used to determine whether APC was statistically different from zero, with no adjustment made for multiple comparisons.

From 2009 to 2023, the number of non-chain outlets per 10,000 population (density) decreased overall from 31.82 to 27.47 (Fig. 1), representing a −13.7% change (Fig. 1b and Table 1), with an AAPC of −1.06% annually (AAPC 95% CI: −1.11%, −1.01%; *P* < 0.001) observed (Fig. 2b).

In all regions, except for North America, there was an average annual reduction in the density of non-chain outlets over the study period (Figs. 1b and 2b), though the timing of changes varied (Table 1). South Asia and sub-Saharan Africa experienced uniform declines. The largest absolute decrease occurred in UMICs, followed by LMICs and HICs (Fig. 1b). Joinpoint analysis showed that the annual percentage decrease in the density of non-chain outlets in HICs was faster before 2016 than during 2016–2020, and then slower after 2020 (Table 2). LMICs showed a significant decrease only before 2021, while UMICs experienced an accelerated decline after 2016 (Table 2).

Results for this indicator were similar to trends for chain and non-chain outlet density. The ratio of non-chain to chain outlets decreased overall by 61.3% between 2009 and 2023 (Fig. 1c), with an AAPC of −6.90% (95% CI: −7.33%, −6.46%; *P* < 0.001). A significant change in trends occurred in 2016, where the overall rate of decrease slowed from an APC of −10.12% (95% CI: −11.53%, −9.05%; *P* < 0.001) to −3.58% (95% CI: −4.66%, −1.99%; *P* < 0.001).

Joinpoint regressions revealed considerable heterogeneity in the pattern of change across regions, although in most regions the decreases observed in the latest segment were smaller than in the preceding segment (Table 1). Changes across income groups showed that despite having the largest number of non-chain outlets per chain outlet, LMICs experienced the most substantial drop in the ratio over the period, with an AAPC of −7.05% (95% CI: −7.41%, −6.60%; *P* < 0.001) (Figs. 1c and 2c). Different paces of reduction in the ratio over time across all income groups were observed (Table 2).

Overall, the grocery sales from chain outlets increased from 50% in 2009 to 58.5% in 2023 (a 17.2% increase) (Fig. 1d), with an AAPC of 1.11% (95% CI: 1.09%, 1.15%; *P* < 0.001) (Fig. 2d). Joinpoint analysis identified a change of trend in 2021, with a slower APC after that year (Table 1).

Sales from chain outlets varied across regions (Fig. 1d). North America and sub-Saharan Africa were the only regions where no changes in the percentage of grocery sales from chain retailers were observed while South Asia had a strikingly high AAPC increase of 6.68% (95% CI: 6.31%, 7.03%; *P* < 0.001) (Fig. 2d). Joinpoint analysis revealed regional variations in the timing of changes (Table 2). By income level, HICs had the higher percentage of grocery sales from chain outlets, while LMICs had lower percentages (Figs. 1d and 2d). Joinpoint analysis showed a consistent increase in the percentage of grocery sales through chain outlets across the study period for UMICs and HICs, but for LMICs, the APC slowed down after 2016 (Table 2).

Between 2009 and 2023, sales of unhealthy foods increased from 75.5 kg per capita to 79.23 kg per capita (a 4.9% increase over the period) (Fig. 1e), with an AAPC of 0.31% (95% CI: 0.21%, 0.37%; *P* < 0.001) (Fig. 2e). Joinpoint analysis identified two trend changes (at 2017 and 2021), but only the first was statistically significant (Table 1). South Asia had a large average annual increase in unhealthy food sales between 2009 and 2023 (AAPC = 3.20%, 95% CI: 2.83%, 3.49%; *P* < 0.001), although this was from a very low base (average sales of 4.35 kg per capita in 2009). No significant changes in sales of unhealthy foods were observed for North America (Fig. 1e).

The AAPC in sales of unhealthy food categories between 2009 and 2023 was higher for LMICs than UMICs, with no significant change observed in HICs (Fig. 2e). Joinpoint analysis showed that the annual increase in unhealthy food sales observed in LMICs was significant only for the segment between 2009 and 2020 (APC = 1.42%, 95% CI: 1.21%, 2.76%; *P* < 0.001), with no significant changes from 2020 onwards (Table 2).

Between 2009 and 2023, the percentage of unhealthy food sales from chain outlets increased from 56.6% to 62.7%, marking a 10.9% rise over the period (Fig. 1f), with an AAPC of 0.81% (95% CI: 0.77%, 0.88%; *P* < 0.001) (Fig. 2f). Two slopes of change were identified (Table 1).

Regionally, there was considerable variation in the sale of unhealthy food from chain outlets, with over 80% of unhealthy food sales in North America from chain retailers and under 30% in South Asia during the study period (Fig. 1f). All regions showed increases in the percentage of unhealthy food sales from chain outlets over time (Fig. 2f), with the timing of those changes varying across regions (Table 1). Variations across income levels were also seen. HICs consistently had the highest percentages of unhealthy sales from chain outlets (over 80% throughout the period), followed by UMICs and LMICs (Fig. 1f). However, UMICs showed the highest AAPC, closely followed by LMICs, with a slower increase in HICs (Fig. 2f). Joinpoint analysis showed a steady growth in unhealthy food sales from chain outlets in UMICs. In LMICs, growth was faster between 2012 and 2018, with a slightly reduced rate of growth thereafter (Table 2). In HICs, growth was faster before 2017 and slower from then on (Table 2).

### Changes in the digital retail food environment from 2014 to 2023

In 2014, grocery sales per capita through digital channels were relatively low for many countries (that is, 13 out of 27 countries had an average annual expenditure of less than US$10 per capita in 2014); there was a rapid and statistically significant increase in purchases through digital channels across all countries (except for Brazil). Overall, from 2014 to 2023, grocery sales through digital channels increased from US$27.4 to US$116.7 per capita annually (a 325% increase), with an AAPC of 20.0% (95% CI: 14.9%, 25.9%; *P* < 0.001). No breakpoints were identified in the overall and income-level analysis, indicating continuous and stable growth since 2014. LMICs had the largest AAPC, followed by UMICs and HICs (Supplementary Fig. 1 and Supplementary Table 11).

### Association between the retail food environment and obesity

Overall, the AAPC in obesity prevalence was 2.05% (95% CI: 2.04%, 2.05%; *P* < 0.01) between 2009 and 2022. The prevalence of obesity increased significantly in all geographic regions and all country income groups over the entire study period, and in each of the segments identified in joinpoint analysis (Supplementary Table 13).

**Fig. 3:**
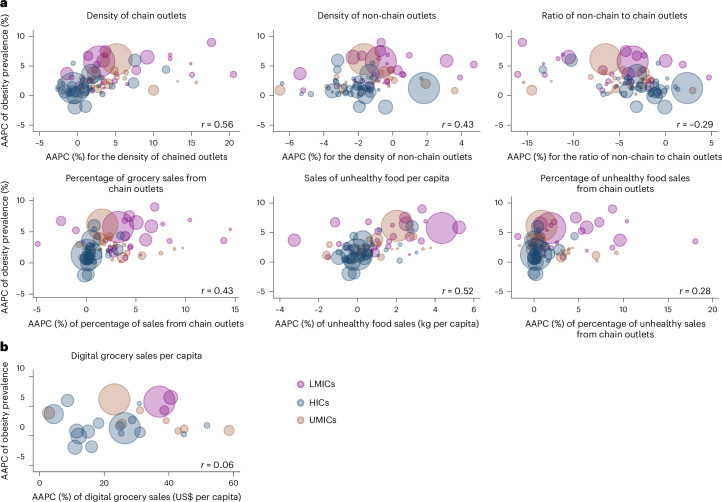
Relationship between the AAPC in retail food environment indicators and AAPC in obesity prevalence, by country. **a**,**b**, The AAPC in physical (**a**) and digital (**b**) retail food environment indicators is shown on the *x* axis, and the AAPC in obesity prevalence is plotted on the *y* axis at the country level. AAPCs were calculated for 2009–2022 for physical retail environments and 2014–2022 for digital retail environments. Each country is represented by a bubble, with the bubble size reflecting its population in 2022. Poland was excluded from the analysis for the density of non-chain outlets and ratio of non-chain to chain outlets because of outlier values. Bubble colours represent the income status of each country. Unadjusted Spearman’s correlation coefficients (*r*) are reported for the association between AAPC in retail food environment indicators and AAPC in obesity prevalence, indicating the strength of the relationship between changes in retail food indicators and obesity prevalence across all countries aggregated.

Moderate positive correlations were observed between the AAPC in the prevalence of obesity and the AAPC for several retail indicators, including the density of chain outlets (*r* = 0.56, *P* < 0.001), the sales of unhealthy foods (*r* = 0.52, *P* < 0.001), the density of non-chain outlets (*r* = 0.42, *P* = 0.002) and the percentage of grocery sales from chain outlets (*r* = 0.43, *P* < 0.001). A weak positive correlation was observed between the AAPC in the prevalence of obesity and the percentage of sales of selected unhealthy food products from chain retailers (*r* = 0.28, *P* < 0.005), while no significant correlation was observed between change in the prevalence of obesity and change in grocery sales through digital channels (*r* = 0.06, *P* = 0.768). A weak negative correlation was observed between the AAPC in the prevalence of obesity and the AAPC in the ratio of non-chain to chain outlets (*r* = −0.29, *P* < 0.004), where countries with a decreasing number of non-chain stores relative to chain stores were seen to have an obesity prevalence increase over the study period (Fig. 3). Correlation analyses stratified by region and country income groups are available in Supplementary Figs. 2–9.

## Discussion

Using data from 97 countries from 2009 to 2023, we showed rapid changes in the retail food environment at a global scale and across geographic regions and country income levels. A significant increase in the density of chain outlets, the percentage of grocery sales from chain outlets, unhealthy food sales per capita and the percentage of unhealthy food sales from chain outlets was observed on a global scale. Concurrently, there was a decline in the density of non-chain outlets and the ratio of non-chain to chain outlets. The ongoing shift from traditional, local food retail to a chained food retail market is correlated with the rising prevalence of obesity, with moderate positive correlations found between the average annual percentage increase in the prevalence of obesity and the change in the density of chain outlets, the percentage of grocery sales from chain retailers and unhealthy food sales between 2009 and 2022. Data from 27 countries between 2014 and 2023 revealed that the digital retail food environment is a rapidly growing market.

While the direction of trends was broadly consistent across geographic regions, North America was a notable exception with a small but statistically significant decline in the density of chain outlets and no change in unhealthy food sales over the study period. Our results show that HICs, particularly those in Europe and Central Asia and North America, represent a more fully ‘corporatized’ food retail sector, characterized by a higher density of chain outlets and higher unhealthy food sales per capita compared with other regions. As the regions with the earliest expansion of large chained retailers, the transformation to a ‘modern’ retail food sector dominated by supermarkets, hypermarkets and convenience stores that began in the early 1990s or earlier in those regions may have largely come to an end^3^. By contrast, LMICs, particularly those in the East Asia and Pacific and South Asia regions, currently maintain a more ‘traditional’ retail food environment, with a greater density of non-chain outlets and a lower percentage of grocery sales from chain retailers. This is rapidly changing, however, with these LMICs experiencing the fastest changes in the food retail landscape over the past 15 years.

Between 2009 and 2023, the period covered by our study, various national, regional and global events markedly impacted the production and distribution of food products. Globally, the COVID-19 pandemic caused widespread disruptions in food supply chains owing to lockdowns and logistical challenges, altering food distribution patterns and people’s purchasing behaviours^27,28^. Although these disruptions were not directly accounted for in our trend analysis, the aggregated data across countries, regions and income levels did not show clear changes in the assessed retail indicators at the time of the pandemic. At the regional and country levels, changes in trends for retail-related indicators should be interpreted in light of relevant external factors, such as country or regionally specific political, economic and social changes that may have affected the retail sector in those contexts.

Despite limited data, our findings reveal the widespread expansion of grocery e-commerce. Although large annual percentage increases were observed in LMICs and UMICs, these were from a low base, with HICs having the highest average annual per capita sales through digital channels. Variation in the use of digital groceries might be explained by contextual factors such as infrastructure, internet accessibility and cultural norms, which can vary between countries and have a strong influence on the growth of digital food retail^29,30^. Further research on the degree to which the digital retail food environment influences the healthiness of customer food purchasing is required, with some early research pointing to extensive marketing of unhealthy foods in these settings^31–33^. The dynamic and highly personalized nature of digital food environments represent an evolving public health challenge, particularly in relation to monitoring for accountability and regulatory initiatives.

While the expansion of the chained food retail sector within countries can present benefits for a growing population, such as increased food safety standards, convenience and cheaper prices due to economies of scale^34^, it also presents several public health challenges. We found that sales of unhealthy foods from chain retailers increased over the past 15 years. Our findings also showed that countries with the highest annual percentage increase in the density of chain outlets, the percentage of grocery sales from chain retailers and the unhealthy food sales per capita were also some of those with the highest annual percentage increase in the prevalence of obesity from 2009 to 2022. Although these are ecological observations, there are several ways in which change in retail mix can be contributing to the increasing obesity prevalence. Firstly, while chain outlets such as supermarkets and hypermarkets may provide a wide variety of healthier food products, they typically also subject customers to aggressive marketing of an enormous array of unhealthy products^35^, exacerbating dietary risks associated with overconsumption of such foods. Secondly, the dominance of a few large, chained retailers grants them considerable corporate power to influence the entire food system, allowing them to manipulate prices to maximize profits and, together with national and multinational food manufacturers, drive the sale of ultra-processed, unhealthy packaged foods^36^. Large multinational food and retail companies have been observed to leverage foreign direct investment to penetrate new markets and consolidate their presence. This strengthens their control over pricing, supply chains and marketing and has been found to result in the greater availability and promotion of unhealthy products in a country^1,19^. Finally, large food manufacturers and retail companies also exert political power by delaying and hindering government regulation that prioritizes public health^36,37^.

Our findings suggest that correlations between change in obesity prevalence and change in retail food environment metrics differ. We hypothesize that this reflects shifts in the food system leading to a nutrition transition from traditional diets to diets increasingly dominated by unhealthy and highly processed foods and the weight gain that follows^9^. The strongest correlations were observed between changes in obesity prevalence and the density of chain retail outlets and unhealthy foods sales. A negative correlation was found between changes in obesity prevalence and the ratio of non-chained to chained outlets, suggesting that countries with a declining number of non-chain stores relative to chain stores experienced rising obesity prevalence over the study period. Although many non-chained outlets also sell unhealthy and highly processed foods, it is modern chained retail that is far more effective and aggressive in promoting these products because of longer opening hours, greater volumes of products sold (especially in supermarkets and hypermarkets) and opportunities provided for chain-wide and sophisticated marketing campaigns^38^. As our results show, the percentage of sales for selected unhealthy food categories from chain retailers increased over time, replacing non-chain retailers as a source of these products. As chained retailers gradually out-compete non-chained retailers with their greater resources and economies of scale^39,40^, an increasingly monopolized food environment is created that promotes the purchase and consumption of industrialized and ultra-processed food at a population scale. Over time, we hypothesize that this is reflected in increases in excess body weight and eventually in increases in the prevalence of obesity. The decline in small traditional food stores, particularly those selling fresh fruits and vegetables, could potentially exacerbate food access problems, particularly in underserved communities where these stores are often relied upon as the daily source of food. In addition, public health benefits of these more ‘traditional’ retail settings can extend beyond nutrition, including the valuable social connections they help establish^41^.

Especially in settings where mean body weight is lower, increases in the prevalence of obesity can be considered a late-stage consequence of food system transition, given that this captures only the movement of individuals into the highest body mass index (BMI) category (≥30 kg m^−^^2^)^42^. We recognize that the correlations between changes in the food retail environment indicators and obesity prevalence reported here represent ecological associations. They should therefore not be interpreted as evidence confirming causality given the possibility of unaccounted confounding factors. While consistent with the hypothesis that changes in retail food environments impact dietary patterns and health^43^, further study is needed to explore these associations. Methodological improvements, including better data on body weight and food sales at the category level, incorporation into analysis of additional potential confounding factors representing the socioeconomic and political context and a dedicated examination of market concentration within the retail sector, could allow a more nuanced exploration of these relationships in the future. Further examination of the impact of changes in retail food environments on planetary health is also urgently needed as beyond their impact on human health, unhealthy and highly processed foods are linked with substantial environmental impacts^12,44^. The World Health Organization 2030 Sustainable Development Goals highlight the importance of sustainable food production and consumption, and the need for the private sector, including retailers, to take action to make this happen^45^.

As this analysis builds on secondary data sources, the limitations mainly relate to the availability, quality and reliability of those secondary data. While 97 countries were included in the analysis, they are not necessarily globally representative, with larger countries over-represented in the Euromonitor database. In our analysis of the digital retail food environment, data were available only for a smaller subset of countries, meaning aggregated summaries by geographic region could not be generated. The Euromonitor Passport database does not capture products sold through informal (non-registered) channels or account for food waste, potentially leading to either overestimation or underestimation of unhealthy food sales. The inability to capture sales from informal food retailers may be a bigger missing piece of the puzzle in LMICs where informal retail food channels are highly prevalent and contribute substantially to the diet of populations^46^. As Euromonitor data include only formal channels, we chose not to analyse sales of healthy food categories (for example, fresh fruits and vegetables) as these are often sold through informal channels (in LMICs in particular), which would have introduced considerable uncertainty and potential biases in our findings. If more complete data on sales of healthy food categories become available from Euromonitor data, analysis of their sales through retail channels would be a valuable addition to this work in future. Due to the lack of detailed nutritional information for individual food products in the Euromonitor database, in the ‘unhealthy’ food sales indicator, we included only selected food categories that have been commonly described as having overall poor nutritional quality^47^, in line with conceptualizations of ‘unhealthy’ in a range of national dietary guidelines^48–50^. Despite this, we acknowledge that some other relevant food products that contribute to the energy density of diets and excessive intake of nutrients of concern may be missing from our analysis. Furthermore, Euromonitor states that data for some of the unhealthy food categories included in our analysis of food sales were modelled from matched approximator countries, for which accuracy has been disputed^51^. In our study, modelled data for unhealthy food sales were used for 45 of the 97 countries included in the analysis, meaning inferences should be drawn with caution for that indicator. The percentage of unhealthy food sales from chain retailers is based on a limited number of food categories owing to data availability and should be interpreted with this limitation in mind. Despite their limitations as noted, Euromonitor databases are a trusted commercial data source that has been used in numerous academic studies^25,52–54^, particularly in research on the retail food environment that is retrospective, covers extensive geographic areas or examines diverse store types^55^. As all data were retrieved from Euromonitor and our focus was on reporting changes over time, we believe that measurement error commonly associated with commercial databases is minimized across all data points and diluted by our emphasis on temporal changes rather than absolute metrics. Given the lack of other comprehensive datasets that retrospectively cover various retail food environment metrics across countries, future studies validating Euromonitor’s food retail data (similar to those conducted for other commercial datasets in the United States, such as InfoUSA and Dun and Bradstreet^56^) would be valuable. In relation to the data analysis, we did not account for the multiple statistical tests conducted in this study, although most of the *P* values reported were highly statistically significant (*P* < 0.001). We also recognize that using a BMI ≥ 30 kg m^−^^2^ as a threshold for obesity probably underestimates the prevalence of obesity in Asian populations^57^ and overestimates it in Black African populations^58^.

In conclusion, our study provides evidence for rapid changes in retail food environments globally, regionally and across country income levels over the past 15 years. An increasingly corporatized (chained) retail food environment is associated with increases in obesity prevalence. Urgent consideration of the impacts of these changes on both human and planetary health is required, especially in LMICs where change is most rapid. For countries in the early stages of a food retail transition, ensuring that corporatized retail food environments are healthy when being established is likely to be far easier than changing them when fully established. Where corporatized retail food environments are already well established, retrofitting them to encourage healthier diets is an enormous challenge for policymakers, public health advocates and retailers.

## Methods

### Retail food environment indicators

Food retail data (number of outlets and food sales) were obtained from the Euromonitor International Passport Global Market Information Database, 2023 Edition^59^. All countries with relevant data available were included in the analysis. Euromonitor applies standardized methods to gather and report market data at a national level, with data sources including trade associations, industry bodies, company financial reports and official government statistics. Euromonitor provides access to data for a range of metrics spanning the past 15 years of date of access (except for the digital sales that are available only since 2014). Data quality checks with in-country experts are undertaken to ensure data accuracy, allowing comparable data across time and between countries. Euromonitor data are widely used by food companies for market research and by academic researchers in peer-reviewed research^25,52,53^.

Seven retail food environment indicators were derived from Euromonitor data, six describing the physical retail food environment and one the digital retail food environment. The physical retail food environment refers to brick-and-mortar settings and was grouped into chain retailers (convenience stores, supermarkets and hypermarkets with more than 10 stores) and non-chain retailers (small independently owned grocery stores with fewer than 10 locations). The digital retail food environment refers to retailers that sell groceries through online platforms, including websites and mobile apps. From 2009 to 2023, 97 countries (44% of all countries worldwide; Supplementary Table 1) had complete data for the six physical retail food environment indicators. Complete data for the digital retail food environment indicator were available only from 2014 to 2023 and for 27 countries (12% of all countries). For detailed definitions of each retailer type and the rationale for the selection of the retail food environment indicators and their definitions, please refer to Supplementary Tables 2 and 3, respectively.

#### Physical retail food environment indicators

For each year (2009–2023) and country (*n* = 97), we calculated the following indicators: (1) the number of chain outlets per 10,000 population (density; annual population data sourced from the World Bank database); (2) the number of non-chain outlets per 10,000 population (density); (3) the ratio of non-chain to chain outlets (number of non-chain outlets divided by the number of chain outlets), where the higher the ratio, the more non-chain food retail outlets exist; (4) total grocery sales (US$) from chain outlets as the percentage of all grocery sales from physical outlets (chained and non-chained); (5) sales of unhealthy food products per capita (kg per capita), where unhealthy food products that are typically high in saturated fats, sodium and/or added sugars were identified using predefined Euromonitor food categories (Supplementary Table 4); and (6) sales of selected unhealthy food products (US$) as the percentage of all sales of unhealthy food from physical outlets (chained and non-chained).

#### Digital retail food environment indicator

For each year (2014–2023) and country (*n* = 27), we calculated the grocery sales per capita (US$ per capita) through digital channels.

### Obesity prevalence

The prevalence of obesity among adults (aged 18 years or older), defined as BMI ≥ 30 kg m^−^^2^, for each year between 2009 and 2022 (last year with data available) for 97 countries was obtained from the NCD Risk Factor Collaboration (NCD-RisC) database^10^. NCD-RisC is a worldwide network of health researchers that collect data on NCD risk factors from 200 countries pooling information from nationally and subnationally representative surveys to produce estimates of the prevalence of NCD risk factors in each country. NCD-RisC data are verified by NCD-RisC members and subsequently peer reviewed and published for a wider academic audience. More information about the NDC-RisC data is available at https://www.ncdrisc.org/.

### Classification of countries

Countries were organized into seven geographic regions according to World Bank lending classifications (East Asia and Pacific, Europe and Central Asia, Latin America and the Caribbean, Middle East and North Africa, North America, South Asia and sub-Saharan Africa)^60^. Countries were also classified into three income groups (LMIC, UMIC and HICs) based on World Bank rankings (Supplementary Table 1).

### Statistical analysis

#### Time trends analysis

We evaluated trends in retail food environment indicators and obesity prevalence using joinpoint regression^61^, to identify the number and temporal location of points where there was a significant change in the slope of a linear time trend on the natural log scale. We selected the grid search method for fitting the model and Monte Carlo permutation tests for determining the optimal number of joinpoints^62^. For the permutation test, an overall significance level of 0.05 was used with Bonferroni adjustment for multiple comparison. A maximum of two joinpoints were allowed for the analysis of the physical retail food environment indicators and obesity trends (2009–2023) and up to one joinpoint for the digital retail food environment indicator as it was available only for a shorter period (2014–2023). The final model estimated the APC in each joinpoint segment and the AAPC, a weighted average of APCs for the entire study period, which allows a single number to represent the trend over multiple years, with 95% CIs. We used *t*-test to assess whether APCs were statistically different from zero. All statistical tests were two sided. We conducted these analyses for retail food environment indicators at the global (overall), geographic region and country income group level. Due to limited data availability on the digital retail food environment indicator, we were not able to analyse this indicator at the geographic region level. To check the assumptions of linearity and homoscedasticity, the residuals from each model were visually inspected. In a sensitivity analysis allowing autocorrelation over time (time lag of 1 year), similar findings were observed (not reported).

#### Association analysis

We also conducted joinpoint analyses by country (results are available in Supplementary Tables 5–13, but are not described) to investigate whether there was an association between changes in the food retail environment indicators and changes in obesity prevalence (2009–2022). The association between the AAPC for each of the seven food retail environment indicators and the AAPC in obesity prevalence was assessed using Spearman’s correlation coefficient (*r*). Correlation coefficients lower than 0.40 were considered weak, between 0.40 and 0.69 were deemed moderate and 0.70 or higher were regarded as strong^63^. Poland was excluded from the analysis for two retail food environment indicators (density of chain outlets and ratio of non-chain to chain outlets) owing to the extremely rapid decreases in the density of non-chain outlets over time and the sensitivity of analysis methods to the presence of outliers.

Joinpoint Trend Analysis software from the Surveillance Research Program of the US National Cancer Institute, version 5.0.2 (Statistical Research and Applications Branch, National Cancer Institute, United States)^64^ and Stata 18 were used.

### Reporting summary

Further information on research design is available in the Nature Portfolio Reporting Summary linked to this article.

## Supplementary information

Supplementary Information Supplementary Figs. 1–9 and Tables 1–13.

Reporting Summary

## Data Availability

Euromonitor Passport data used for all analysis are not publicly available. Data were accessed through institutional Deakin University access, and their policy prevents us from sharing it publicly. Obesity prevalence data are publicly available and can be obtained without restrictions from the NCD-RisC website at https://www.ncdrisc.org/.
