# Supplementary material for: Global food retail environments are increasingly dominated by large chains and linked to the rising prevalence of obesity
Source: Nat Food. 2025 Mar 3;6(3):283–95. doi: 10.1038/s43016-025-01134-x (PMC11932928; doi:10.1038/s43016-025-01134-x)
Supplement: Supplementary file 1 — Supplementary Figs. 1–9 and Tables 1–13. [file 43016_2025_1134_MOESM1_ESM.pdf]

nature food

Supplementary information https://doi.org/10.1038/s43016-025-01134-x

Global food retail environments are increasingly dominated by large chains and linked to the rising prevalence of obesity

In the format provided by the authors and unedited

Table of contents

Supplementary Figures 2

Supplementary Figure 1. Average annual percent change for food sales through digital channels overall, by income groups, and at national level. 2

Supplementary Figure 2. Distribution of the average annual percent change in the density of chain outlets and obesity prevalence, by region. 3

Supplementary Figure 3. Distribution of the average annual percent change in the density of non-chain outlets and obesity prevalence, by region. 4

Supplementary Figure 4. Distribution of the average annual percent change in the ratio of non-chain to chain outlets and obesity prevalence, by region. 5

Supplementary Figure 5. Distribution of the average annual percent change in unhealthy food sales and obesity prevalence, by region. 6

Supplementary Figure 6. Distribution of the average annual percent change in the density of chain outlets and obesity prevalence, by country income groups. 7

Supplementary Figure 7. Distribution of the average annual percent change in the density of non-chain outlets and obesity prevalence, by country income groups. 8

Supplementary Figure 8. Distribution of the average annual percent change in the ratio of non-chain to outlets and obesity prevalence, by country income groups. 9

Supplementary Figure 9. Distribution of the average annual percent change in the unhealthy food sales and obesity prevalence, by country income groups. 10

Supplementary Tables 11

Supplementary Table 1. Number of countries with data available according to the type of indicator, by geographic region and country income group. 11

Supplementary Table 2. Retail type definitions 12

Supplementary Table 4. Food groups classified as 'unhealthy'. 16

Supplementary Table 5. Density of chain food retail outlets per 10,000 population from 2009 to 2023, by country: joinpoint regression analysis. 17

Supplementary Table 6. Density of non-chain food retail outlets per 10,000 population from 2009 to 2023, by country: joinpoint regression analysis. 21

Supplementary Table 7. Ratio of non-chain to chain outlets from 2009 to 2023, by country: joinpoint regression analysis. 25

Supplementary Table 8. Percentage of sales from chain retailers from 2009 to 2023, by country: joinpoint regression analysis. 29

Supplementary Table 9. Unhealthy food sales (kg per capita) from 2009 to 2023, by country: joinpoint regression analysis. 33

Supplementary Table 10. Percentage of unhealthy food sales for selected food categories from chain retailers from 2009 to 2023, by country: joinpoint regression analysis. 37

Supplementary Table 11. Grocery sales per capita (US$/year) through digital channels, overall and by income group and at national level, 2014–2023 based on joinpoint analysis including 27 countries. 41

Supplementary Table 12. Obesity prevalence from 2009 to 2023, by geographic region and country income group: joinpoint regression analysis. 42

Supplementary Table 13. Obesity prevalence from 2009 to 2023, by country: joinpoint regression analysis. 43

Supplementary Figures

Supplementary Figure 1. Average annual percent change for food sales through digital channels overall, by income groups, and at national level.

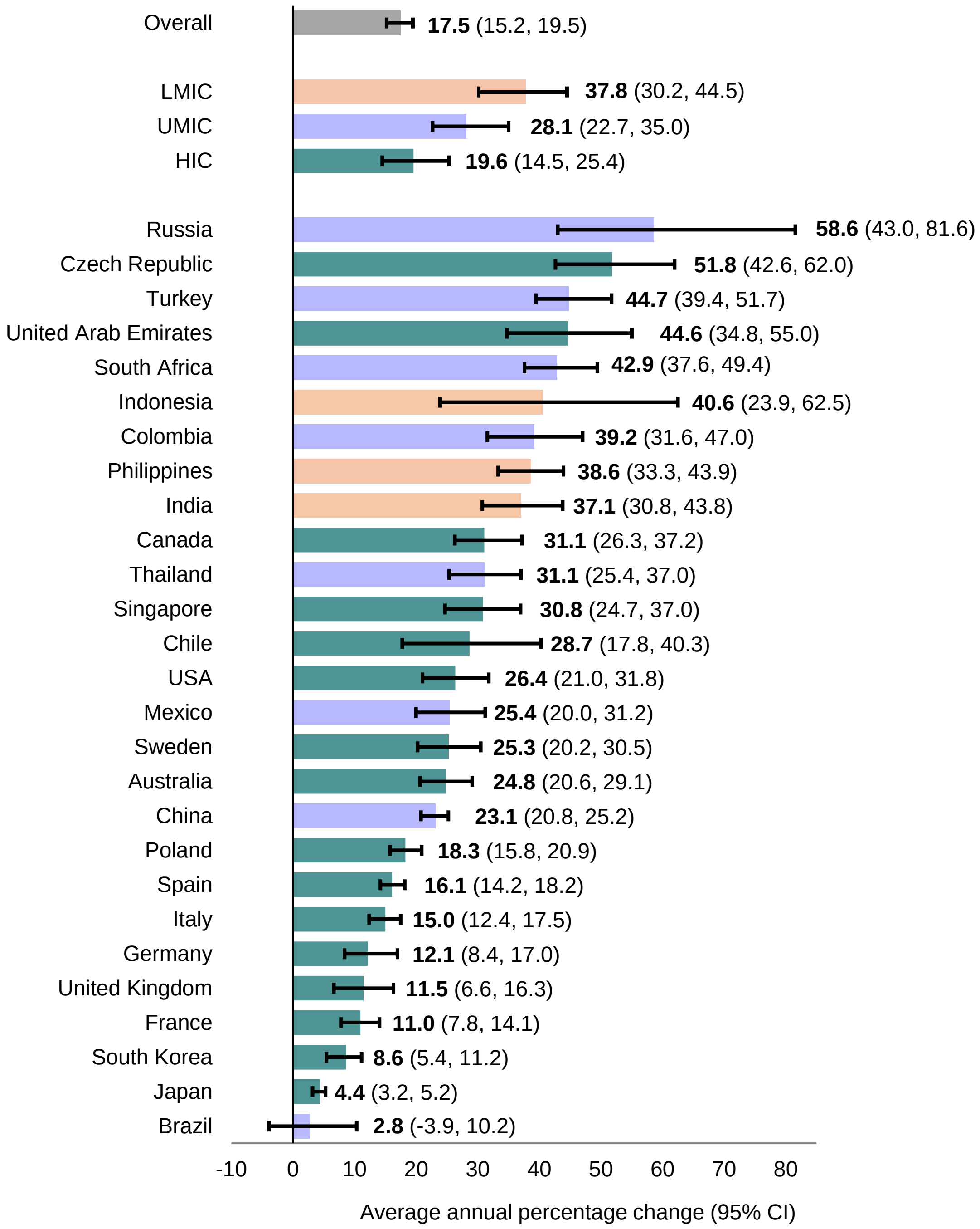

Legend: Annual Average Percent Change (AAPC) was calculated using Joinpoint regression analysis for each country from 2014 to 2023. Error bars represent the 95% Confidence Intervals (CI) derived from the regression analysis. LMIC = Low- and middle-income countries; UMIC = Upper-middle-income countries; HIC = High-income countries. Food sales represent the average annual US$ per capita spent on grocery shopping through digital retail channels.

Supplementary Figure 2. Distribution of the average annual percent change in the density of chain outlets and obesity prevalence, by region.

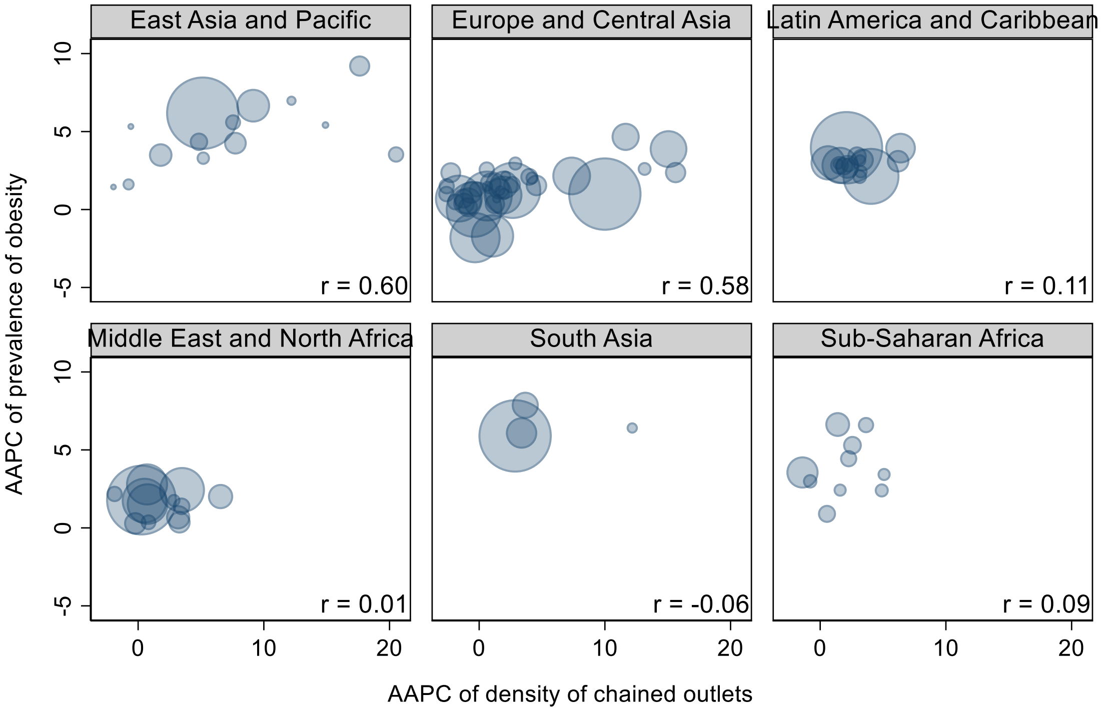

Legend: The Average Annual Percent Change (AAPC) for the density of chain outlets is shown on the X-axis, and the AAPC for obesity prevalence is plotted on the Y-axis at the country level, by region. AAPCs were calculated for 2009–2022. Each country is represented by a bubble, with the bubble size reflecting its population in 2022. Unadjusted Spearman's correlation coefficients (r) are reported for the association between changes in the AAPC of chain outlet density and changes in the AAPC of obesity prevalence, indicating the strength of the relationship by region. North America was excluded from the regional analysis as it contains only two countries.

Supplementary Figure 3. Distribution of the average annual percent change in the density of non-chain outlets and obesity prevalence, by region.

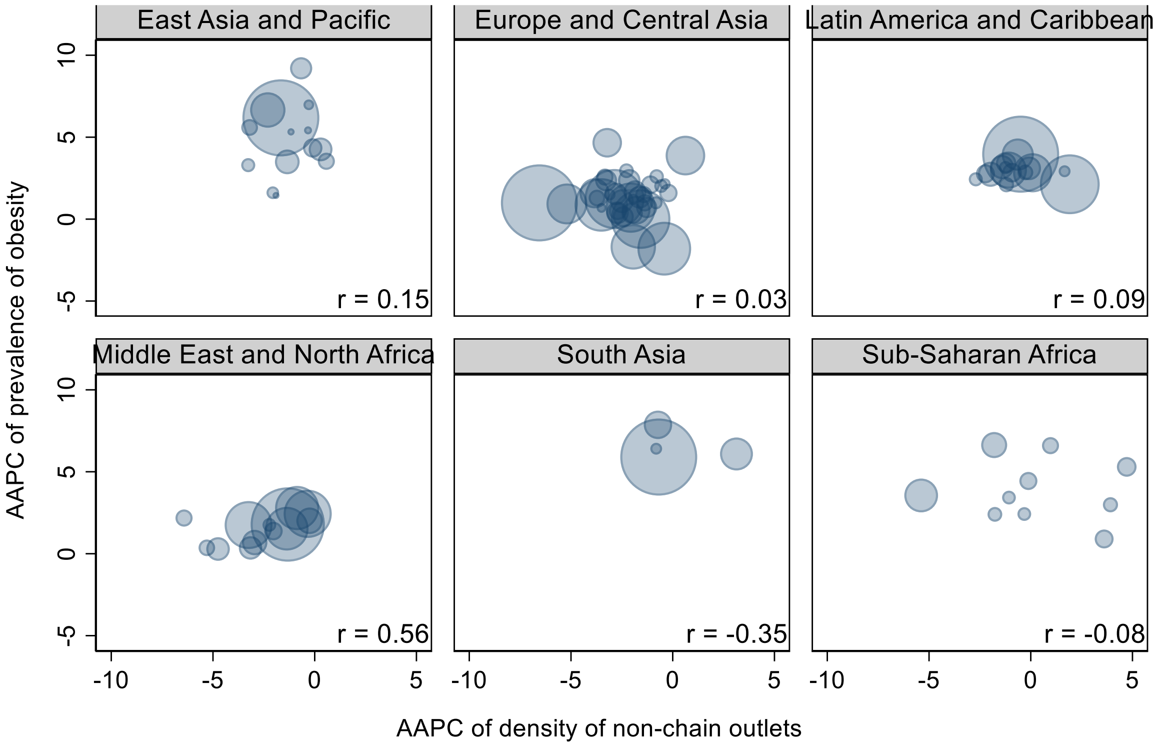

Legend: The Average Annual Percent Change (AAPC) for the density of non-chain outlets is shown on the X-axis, and the AAPC for obesity prevalence is plotted on the Y-axis at the country level, by region. AAPCs were calculated for 2009–2022. Each country is represented by a bubble, with the bubble size reflecting its population in 2022. Unadjusted Spearman's correlation coefficients (r) are reported for the association between changes in the AAPC of density of non-chain outlets and changes in the AAPC of obesity prevalence, indicating the strength of the relationship by region. North America was excluded from the regional analysis as it contains only two countries. Poland was excluded from this analysis due to its outlier status.

Supplementary Figure 4. Distribution of the average annual percent change in the ratio of non-chain to chain outlets and obesity prevalence, by region.

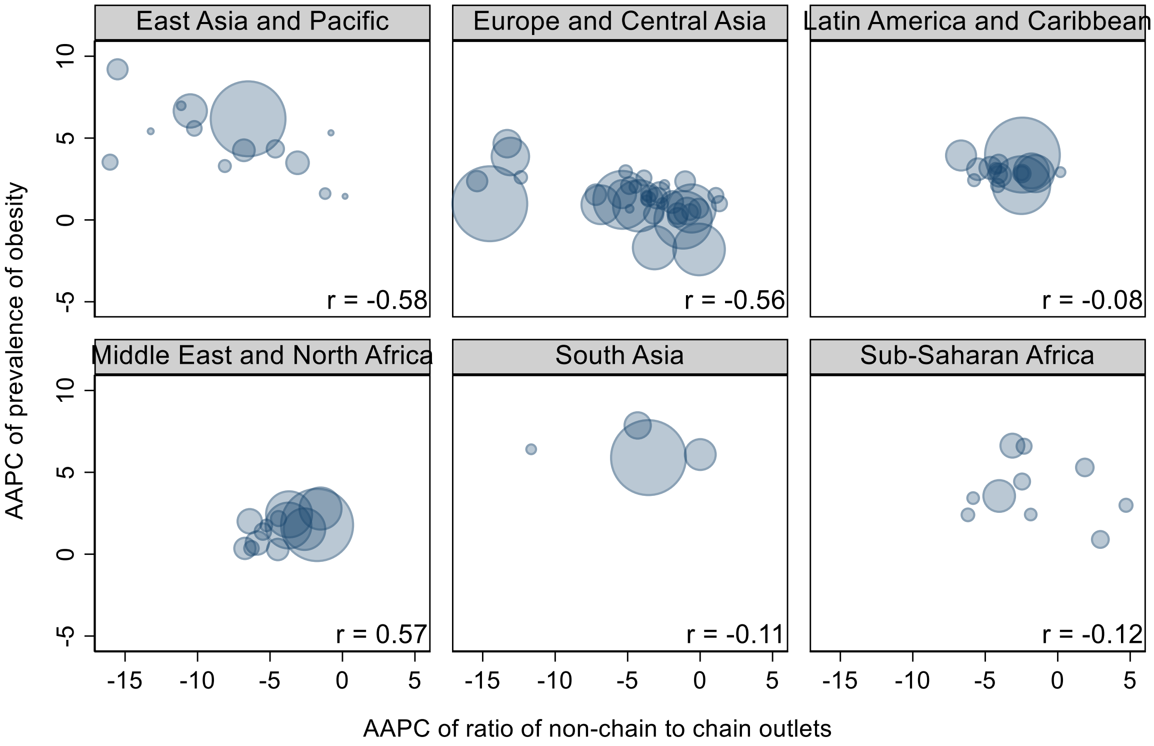

Legend: The Average Annual Percent Change (AAPC) for the ratio of non-chain to chain outlets is shown on the X-axis, and the AAPC for obesity prevalence is plotted on the Y-axis at the country level, by region. AAPCs were calculated for 2009–2022. Each country is represented by a bubble, with the bubble size reflecting its population in 2022. Unadjusted Spearman's correlation coefficients (r) are reported for the association between changes in the AAPC of the ratio of non-chain to chain outlets and changes in the AAPC of obesity prevalence, indicating the strength of the relationship by region. North America was excluded from the regional analysis as it contains only two countries. Poland was excluded from this analysis due to its outlier status.

Supplementary Figure 5. Distribution of the average annual percent change in unhealthy food sales and obesity prevalence, by region.

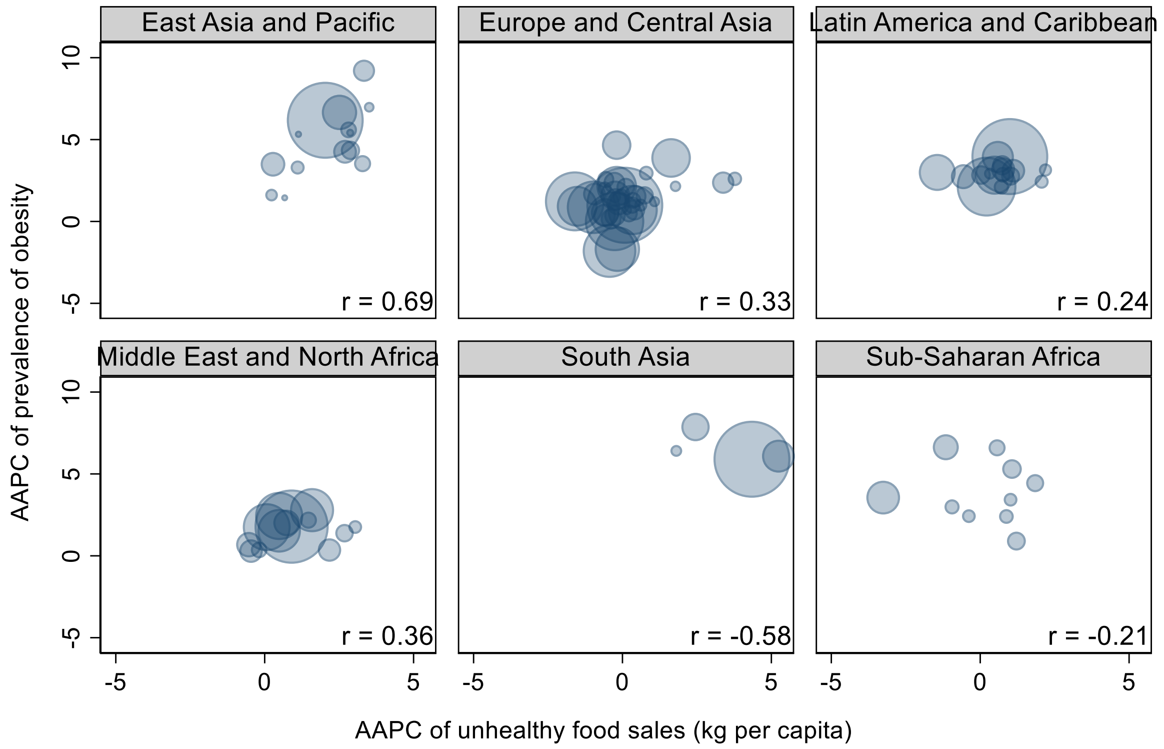

Legend: The Average Annual Percent Change (AAPC) for the unhealthy food sales per capita is shown on the X-axis, and the AAPC for obesity prevalence is plotted on the Y-axis at the country level, by region. AAPCs were calculated for 2009–2022. Each country is represented by a bubble, with the bubble size reflecting its population in 2022. Unadjusted Spearman's correlation coefficients (r) are reported for the association between changes in the unhealthy food sales per capita and changes in the AAPC of obesity prevalence, indicating the strength of the relationship by region. North America was excluded from the regional analysis as it contains only two countries.

Supplementary Figure 6. Distribution of the average annual percent change in the density of chain outlets and obesity prevalence, by country income groups.

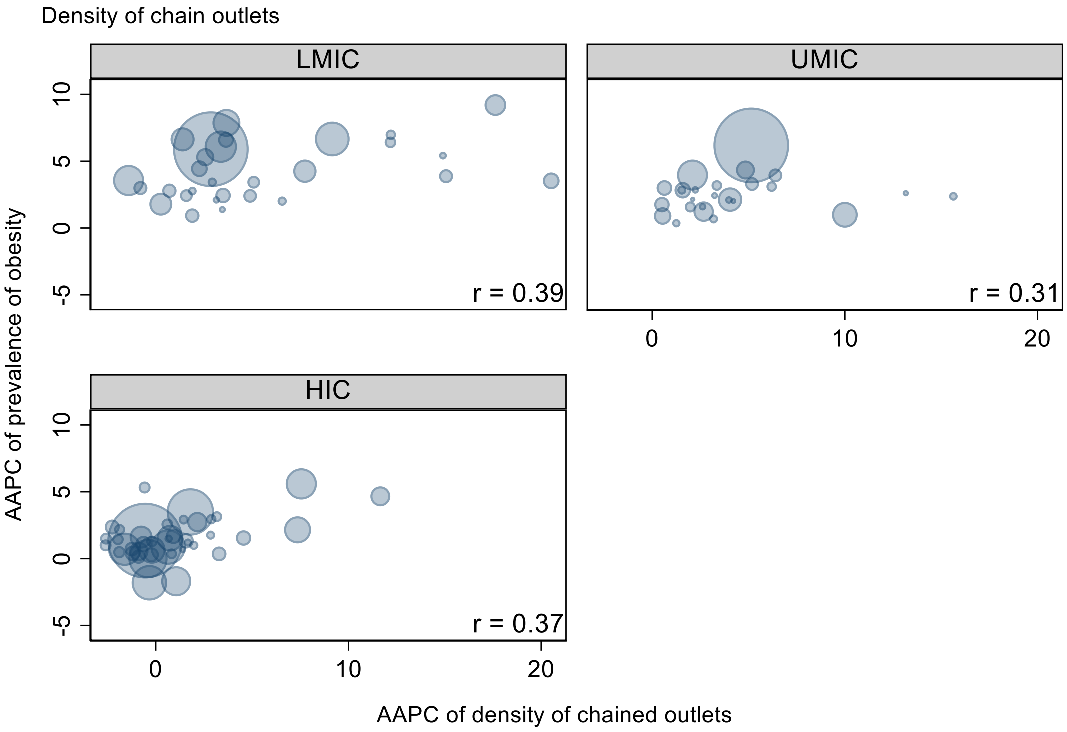

Legend: The Average Annual Percent Change (AAPC) for the density of chain outlets is shown on the X-axis, and the AAPC for obesity prevalence is plotted on the Y-axis at the country level, by country income groups. AAPCs were calculated for 2009–2022. Each country is represented by a bubble, with the bubble size reflecting its population in 2022. Unadjusted Spearman's correlation coefficients (r) are reported for the association between changes in the density of chain outlets and changes in the AAPC of obesity prevalence, indicating the strength of the relationship by income group. LMIC = Low-and-middle income countries; UMIC = Upper-middle income countries; HIC = High-income countries.

Supplementary Figure 7. Distribution of the average annual percent change in the density of non-chain outlets and obesity prevalence, by country income groups.

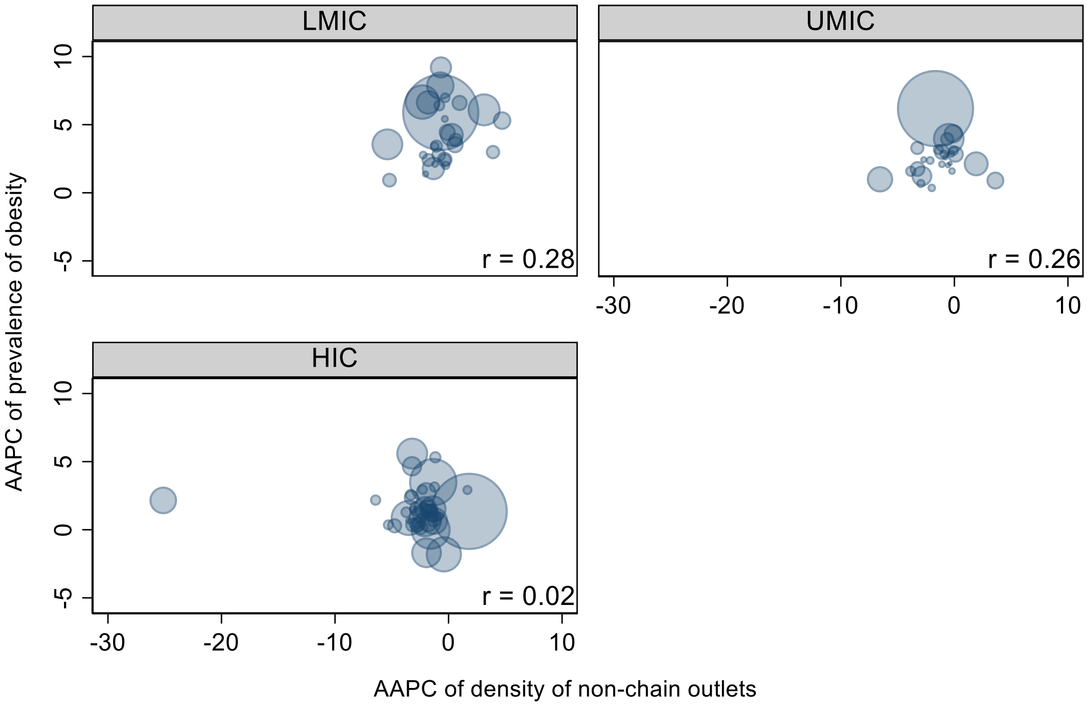

Legend: The Average Annual Percent Change (AAPC) for the density of non-chain outlets is shown on the X-axis, and the AAPC for obesity prevalence is plotted on the Y-axis at the country level, by country income groups. AAPCs were calculated for 2009–2022. Each country is represented by a bubble, with the bubble size reflecting its population in 2022. Unadjusted Spearman's correlation coefficients (r) are reported for the association between changes in the density of non-chain outlets and changes in the AAPC of obesity prevalence, indicating the strength of the relationship by income group. Poland was excluded from this analysis due to its outlier status. LMIC = Low-and-middle income countries; UMIC = Upper-middle income countries; HIC = High-income countries.

Supplementary Figure 8. Distribution of the average annual percent change in the ratio of non-chain to outlets and obesity prevalence, by country income groups.

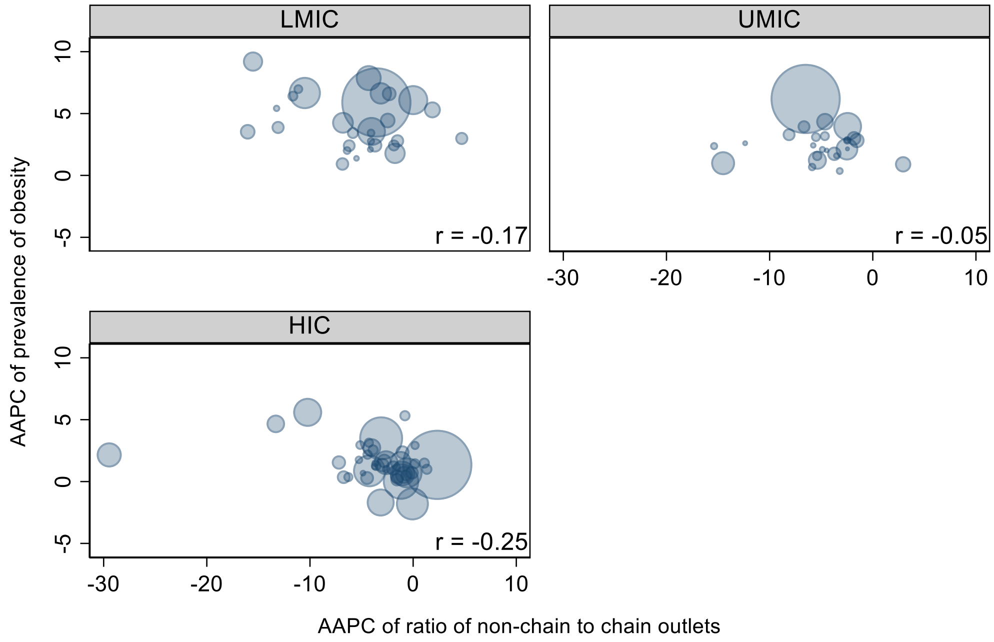

Legend: The Average Annual Percent Change (AAPC) for the ratio of non-chain to chain outlets is shown on the X-axis, and the AAPC for obesity prevalence is plotted on the Y-axis at the country level, by country income groups. AAPCs were calculated for 2009–2022. Each country is represented by a bubble, with the bubble size reflecting its population in 2022. Unadjusted Spearman's correlation coefficients (r) are reported for the association between changes in the ratio of non-chain to chain outlets and changes in the AAPC of obesity prevalence, indicating the strength of the relationship by income group. Poland was excluded from this analysis due to its outlier status. LMIC = Low-and-middle income countries; UMIC = Upper-middle income countries; HIC = High-income countries.

Supplementary Figure 9. Distribution of the average annual percent change in the unhealthy food sales and obesity prevalence, by country income groups.

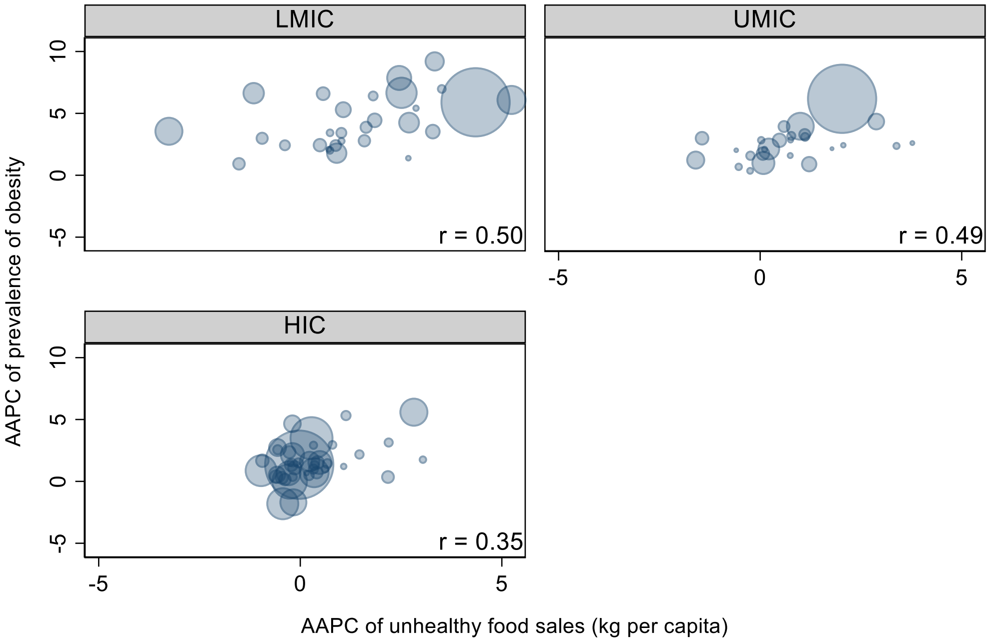

Legend: The Average Annual Percent Change (AAPC) for the unhealthy food sales per capita is shown on the X-axis, and the AAPC for obesity prevalence is plotted on the Y-axis at the country level, by country income groups. AAPCs were calculated for 2009–2022. Each country is represented by a bubble, with the bubble size reflecting its population in 2022. Unadjusted Spearman's correlation coefficients (r) are reported for the association between changes in the unhealthy food sales per capita and changes in the AAPC of obesity prevalence, indicating the strength of the relationship by income group. LMIC = Low-and-middle income countries; UMIC = Upper-middle income countries; HIC = High-income countries.

Supplementary Tables

Supplementary Table 1. Number of countries with data available according to the type of indicator, by geographic region and country income group.

Geographic region	Number of countries	LMIC	UMIC	HIC	Percentage of countries covered in this region[c]
		n (%)	n (%)	n (%)	
Physical retail food environment indicators[a]					
East Asia and Pacific	14	6 (43%)	3 (21%)	5 (36%)	37%
South Asia	4	4 (100%)	0	0	50%
Europe and Central Asia	38	2 (5%)	10 (26%)	26 (69%)	66%
Middle East and North Africa	13	5 (39%)	2 (15%)	6 (46%)	62%
Sub-Saharan Africa	10	9 (90%)	1 (10%)	0	21%
Latin America and Caribbean	16	3 (19%)	10 (62%)	3 (19%)	38%
North America	2	0	0	2 (100%)	67%
**Overall**	**97**	**29 (30%)**	**26 (27%)**	**42 (43%)**	**44%**
Digital retail food environment indicator[b]					
East Asia and Pacific	8	2 (25%)	2 (25%)	4 (50%)	21%
South Asia	1	1 (100%)	-	-	13%
Europe and Central Asia	10	-	2 (20%)	8 (80%)	17%
Middle East and North Africa	1	-	-	1 (100%)	5%
Sub-Saharan Africa	1	-	1 (100%)	-	2%
Latin America and Caribbean	4	-	3 (75%)	1 (25%)	10%
North America	2	-	-	2 (100%)	67%
**Overall**	**27**	**3 (10%)**	**8 (30%)**	**16 (60%)**	**12%**

LMIC = Low-and-middle income countries; UMIC = Upper-middle income countries; HIC = high-income countries.

[a] Indicators included: density of chain outlets, density of non-chain outlets, ratio of non-chain to chain outlets, proportion of sales from chain retailers, sales of 'unhealthy' foods, percentage of unhealthy food sales from chain retailers.

[b] Indicator included: per capita grocery sales through digital channels.

[c] Percentage of countries included in our analysis of the total number of countries in the region (based on listing of countries at the World Bank).

Supplementary Table 2. Retail type definitions

Type of retail	Definition (according to Euromonitor Passport)
**Chain**	
Convenience stores retailers	Chained (with more than 10 stores) grocery retail outlets selling a wide range of groceries and fitting several of the following characteristics: extended opening hours, selling area of less than 400 square metres, offer foodservice products prepared take-away, made-to-order, and hot foods.
Supermarkets	Chained (with more than 10 stores) grocery retail outlets selling groceries like non-perishable products (e.g., rice, pasta and sauces), fruit and vegetables, beverages and household products. Outlets usually have a selling space of between 400 and 2,500 square metres.
Hypermarkets	Chained (with more than 10 stores) grocery retail outlets selling a range of grocery and non-grocery merchandise, with over 2,500 square metres selling space. Hypermarkets are frequently located on out-of-town sites or as the anchor store in a shopping centre.
**Non-chain**	
Small local grocers	Small local grocers are mostly independent retail outlets (with a selling space of under 400 square metres), kiosks, market stalls or fixed street vendors, owned by families and/or run on an individual basis, and with a primary focus on selling food/beverages/tobacco and other groceries. Euromonitor describe small local grocers as also including ethnic grocery stores that specialize in foreign brands and food types, health food stores, confectioners/ tobacconists /newsagents (CTNs), food & drink souvenir stores, legally registered mobile shops that are run out of a truck, and regional specialty stores.
**Digital retail food environment**	
Digital grocery channels (e-commerce grocery retailers)	Websites and mobile apps of chain grocery retailers. Sales data includes item purchased via the internet and delivered to customers' homes or made available for pickup at a designated location. It includes services offered by both pure-play online grocery retailers and the online platforms of traditional brick-and-mortar chain grocery stores.

Supplementary Table 3. Description of retail food environment indicators reported in the study.

Reported Indicator	Original Euromonitor Metrics Used	Calculation Methodology (for country-level data)	Rationale for Chosen Indicator
Density of chain outlets	• Number of convenience store outlets<br>• Number of supermarket outlets<br>• Number of hypermarket outlets	Sum of the number of each type of chain outlet, divided by the population* of each country multiplied by 10,000.<br><br>*Annual population data sourced from the World Bank database	The density of retail outlets is the most commonly used metric in studies examining the spatial distribution of retail outlets and its association with obesity.[1] National outlet density per capita can be considered a valid overall representation of the degree to which a country is saturated by a particular retail type, overcoming limitations associated with smaller-scale (i.e., city level) or individual-level (i.e., proximity of retail outlets) analyses previously described.[2]
Density of non-chain outlets	• Number of small local grocery outlets	Number of small local grocery outlets divided by the population* of each country multiplied by 10,000.<br><br>*Annual population data sourced from the World Bank database	
Ratio of non-chain to chain outlets	• Number of convenience store outlets<br>• Number of supermarket outlets<br>• Number of hypermarket outlets<br>• Number of small local grocery outlets	Number of small local grocery outlets divided by the sum of the number of chain outlets (convenience stores, supermarkets, and hypermarkets).	Provides a metric that reflects the relative number of chained and non-chained retail outlets. Relative food retail outlet metrics have been consistently demonstrated to yield the largest effect sizes, support clear, positive dose-response associations with weight status, and provide the best model fit, making them highly recommended for use in retail food environment research.[3,4]
Percentage of total grocery sales from chain outlets	• Retail revenue excluding sales tax (USD per capita) from convenience store retailers<br>• Retail revenue excluding sales tax (USD per capita) from supermarket retailers<br>• Retail revenue excluding sales tax (USD per capita) from hypermarket retailers<br>• Retail revenue excluding sales tax (USD per capita) from small local grocers	Total grocery sales (retail revenue USD) from chain outlets (convenience stores, supermarkets and hypermarkets) as percentage of all grocery sales in chain and non-chain outlets	The percentage of total grocery sales from chain outlets was included to provide insights into the extent and pace at which large chain retailers are capturing market share from smaller non-chain retailers. This indicator complements the density of outlets indicators (i.e., availability and exposure to food outlets) by reflecting changes in food purchasing across both chain and non-chain retailers. The percentage of grocery sales from non-chain retailers are the mirror image of this data, given that total grocery sales are the sum of sales from both retail channels (chained and non-changed).

Sales of unhealthy food products per capita	Retail volume (kg) per capita for each unhealthy food category assessed (see Supplementary Table 4)	Sum of retail volume (kg) per capita for all selected unhealthy food categories	The sales of unhealthy foods per capita was included to provide an indication of the impact of retail structure on the type of food sold. This type of metric has been used in previous food retail research.[5,6] Data was available at a food category level, with categories being classified as "unhealthy" based on prior research showing that the selected categories typically contain foods with poor nutritional quality across packaged food supplies worldwide,[7,8,9,10,11] with most products within those food categories containing high levels of nutrients of concern.[8] The food categories in our analysis align with previous research[12,13] using Euromonitor data to explore unhealthy foods. The selected food categories are also widely recognised as unhealthy in national dietary guidelines.[14,15,16] Even though detailed nutritional information for individual food products included in each category was not available, the categories described as unhealthy were all considered to contain few if any products that could be described as healthy (selected categories described in Supplementary Table 4).
Percentage of total sales of selected unhealthy food products from chain outlets	• Retail revenue excluding sales tax (USD million) for each selected unhealthy food category from convenience store retailers<br>• Retail revenue excluding sales tax (USD million) for each selected unhealthy food category from supermarket retailers<br>• Retail revenue excluding sales tax (USD million) for each selected unhealthy food category from hypermarket retailers<br>• Retail revenue excluding sales tax (USD million) for each selected unhealthy food category from small local grocers.	Total sales (retail revenue USD)of selected* unhealthy food products from chain outlets (convenience stores, supermarkets and hypermarkets) as percentage of all sales of selected unhealthy food products in chain and non-chain outlets<br><br>*Unhealthy food categories included in this indicator were: Baked goods, Breakfast Cereals, Confectionery, Sweet Spreads, Processed Meat, Seafood and Alternatives to Meat, Sauces, Dips and Condiments, Savoury Snacks, and Sweet Biscuits, Snack Bars and Fruit Snacks.	Similar rationale to that described for the percentage of total grocery sales from chain outlets, but this metric aimed to capture the sales of unhealthy foods by chain retailers compared to non-chain retailers. A smaller number of unhealthy food categories (compared to the total sales of unhealthy foods per capita, indicator 'Sales of unhealthy food products per capita') was included as Euromonitor did not provide sales by retail chain for all food categories. The percentage of unhealthy food sales from non-chain retailers are the mirror image of this data, given that total unhealthy food sales are the sum of sales from both retail channels.
Grocery sales per capita through digital channels	• Retail revenue excluding sales tax (USD) per capita from e-commerce grocery retailers	None, reported as the original metric	The inclusion of this indicator is justified by the ongoing expansion in the use of online retail food environments by customers[17] and its potential role in promoting unhealthy dietary behaviours.[18]

1 Pineda E, Stockton J, Scholes S, Lassale C, Mindell JS. Food environment and obesity: a systematic review and meta-analysis. BMJ Nutrition, Prevention & Health. 2024.
2 Cameron et al. A proposed research agenda for promoting healthy retail food environments in the East Asia-Pacific region. Curr Nutr Rep. 2021;10(4):267-281.
3 Wilkins E, Morris M, Radley D, Griffiths C. Methods of measuring associations between the retail food environment and weight status: importance of classifications and metrics. SSM-Population Health. 2019; 1;8:100404.
4 Clary CM, Ramos Y, Shareck M, Kestens Y. Should we use absolute or relative measures when assessing foodscape exposure in relation to fruit and vegetable intake? Evidence from a wide-scale Canadian study. Preventive Medicine. 2015; 1;71:83-7.
5 Huse et al. The nutrition transition, food retail transformations, and policy responses to overnutrition in the East Asia region: a descriptive review. Obesity Reviews. 2022;23(4):e13412.
6 Baker P, Friel S. Food systems transformations, ultra-processed food markets and the nutrition transition in Asia. Globalization and Health 2016; 12(1): 80.
7 Dunford et al. A comparison of the healthiness of packaged foods and beverages from 12 countries using the Health Star Rating nutrient profiling system, 2013–2018. Obesity Reviews. 2019; 20:107-15.
8 Li et al. Nutritional quality of pre-packaged foods in China under various nutrient profile models. Nutrients. 2022; 29;14(13):2700.
9 Mora-Plazas M, Gómez LF, Miles DR, Parra DC, Taillie LS. Nutrition quality of packaged foods in Bogotá, Colombia: a comparison of two nutrient profile models. Nutrients. 2019; 4;11(5):1011.

[10] Crino et al. Measuring the Healthiness of the Packaged Food Supply in Australia. Nutrients. 2018; 31;10(6):702.
[11] Vergeer L et al. A comparison of the nutritional quality of products offered by the top packaged food and beverage companies in Canada. BMC Public Health. 2020; 20:1-4.
[12] Baker et al. Ultra-processed foods and the nutrition transition: Global, regional and national trends, food systems transformations and political economy drivers. Obesity Reviews 2020; 21(12): e13126.
[13] Liang W, Sivashankar P, Hua Y, Li W. Global food expenditure patterns diverge between low-income and high-income countries. Nature Food. 2024;5(7):592-602.
[14] Cámara M, Giner RM, González-Fandos E, López-García E, Mañes J, Portillo MP, Rafecas M, Domínguez L, Martínez JA. Food-based dietary guidelines around the world: a comparative analysis to update AESAN scientific committee dietary recommendations. Nutrients. 2021;8;13(9):3131.
[15] Herforth A, Arimond M, Álvarez-Sánchez C, Coates J, Christianson K, Muehlhoff E. A Global Review of Food-Based Dietary Guidelines. Advances in Nutrition. 2019; 10(4): 590-605.
[16] Anastasiou et al. From harmful nutrients to ultra-processed foods: exploring shifts in 'foods to limit' terminology used in national food-based dietary guidelines. Public Health Nutrition. 2023; 26(11): 2539-50.
[17] Gupta et al. Use of online food delivery services among adults in five countries from the International Food Policy Study 2018–2021. Preventive Medicine Reports 2024; 43: 102766.
[18] Bennett R, Keeble M, Zorbas C, Sacks G, Driessen C, Grigsby-Duffy L, Adams J, Burgoine T, Backholer K. The potential influence of the digital food retail environment on health: A systematic scoping review of the literature. Obesity Reviews. 2024;25(3):e13671.

Supplementary Table 4. Food groups classified as 'unhealthy'.

'Unhealthy' food category	Foods included[a]
Baked goods	Dessert mixes, frozen baked goods, packaged cakes, packaged flat bread, packaged leavened bread, packaged pastries
Breakfast Cereals	Ready-to-eat and hot cereals
Confectionery	Chocolate confectionery, sugar confectionery and gum
Sweet Spreads	Jams and preserves, honey, chocolate spreads, and nut and seed-based spreads
Margarine and Spreads	Margarines and other vegetable-oil based spreads
Processed Cheese	Only includes spreadable (cream cheese) and processed (slices/singles, smoked, cheese sticks, cheese strings) cheese
Flavoured Yoghurts	Yoghurts containing artificial or natural fruit flavours or other added sugars
Chilled and Shelf Stable Desserts	Ready-to-eat dairy desserts (e.g., flans, crème caramels, mousses, cream desserts, gelatine desserts) either chilled or not. Includes chilled snacks.
Other dairy-based products	Coffee whiteners, flavoured condensed milk, flavoured fromage frais and quark.
Frozen processed potatoes	Frozen processed potatoes
Ice cream	Frozen yoghurt, impulse ice cream, plant-based ice cream, unpackaged ice cream and take-home ice cream
Instant Noodles	Instant noodles
Processed Meat, Seafood and Alternatives to Meat	Processed meat, processed seafood and meat and seafood substitutes
Ready meals	Shelf stable, frozen, dried, chilled ready meals, dinner mixes, frozen pizza, chilled pizza, food kits (e.g., tacos), dried (dehydrated formats), shelf stable and chilled and frozen soups. Pre-packaged salads and sandwiches were NOT included in this category.
Sauces, Dips and Condiments	This is the aggregation of cooking ingredients and condiments, dips, pickled products, sauces, yeast-based spreads, and other sauces, dips and condiments
Savoury Snacks	Chips/crisps, extruded snacks, tortilla/corn chips, popcorn, pretzels, nuts and other savoury snacks
Sweet Biscuits, Snack Bars and Fruit Snacks	Biscuits, snack barns (granola bars, breakfast bars, fruit bars), fruit snacks (dried fruits and processed fruit snacks)

[a] Foods included in each category, as per Euromonitor Passport database.

Supplementary Table 5. Density of chain food retail outlets per 10,000 population from 2009 to 2023, by country: joinpoint regression analysis.

	Income status	Density per 10,000 population		AAPC		Segment 1			Segment 2			Segment 3		
		2009	2023	AAPC (95% CI)	p-value	Years	APC (95% CI)	p-value	Years	APC (95% CI)	p-value	Years	APC (95% CI)	p-value
**East Asia and Pacific**														
Australia	HIC	5.05	4.55	-0.76 (-1.02, -0.51)	<0.01	2009-2023	-0.76 (-1.02, -0.51)	<0.01	–	–	–	–	–	–
Cambodia	LMIC	0.08	0.43	12.2 (11.61, 12.94)	<0.01	2009-2011	11.3 (7.06, 18.04)	<0.01	2011-2017	19.16 (7.91, 22.22)	<0.01	2017-2023	5.93 (3.6, 7.81)	<0.01
China	UMIC	1.02	2.10	5.14 (4.83, 5.46)	<0.01	2009-2014	9.03 (7.74, 10.56)	<0.01	2014-2023	3.04 (2.47, 3.55)	<0.01	–	–	–
Indonesia	LMIC	0.48	1.61	9.16 (8.93, 9.41)	<0.01	2009-2015	15.38 (14.74, 16.09)	<0.01	2015-2023	4.72 (4.28, 5.14)	<0.01	–	–	–
Japan	HIC	5.20	6.70	1.8 (1.7, 1.92)	<0.01	2009-2011	0.9 (0.14, 1.87)	0.02	2011-2015	4.15 (3.6, 4.52)	<0.01	2015-2023	0.87 (0.69, 1.02)	<0.01
Laos	LMIC	0.03	0.20	14.91 (13.63, 16.46)	<0.01	2009-2011	10.4 (0.95, 23.82)	0.03	2011-2015	39.36 (30.35, 45.21)	<0.01	2015-2023	5.39 (2.74, 7.44)	0.0104
Malaysia	UMIC	1.65	3.38	5.18 (4.87, 5.42)	<0.01	2009-2019	5.92 (5.63, 6.34)	<0.01	2019-2023	3.35 (1.25, 4.37)	0.0188	–	–	–
Myanmar	LMIC	0.01	0.15	20.52 (18.67, 22.27)	<0.01	2009-2018	29.82 (26.92, 33.61)	<0.01	2018-2023	5.43 (-1.99, 11.04)	0.1064	–	–	–
New Zealand	HIC	6.21	4.65	-1.96 (-2.31, -1.6)	<0.01	2009-2023	-1.96 (-2.31, -1.6)	<0.01	–	–	–	–	–	–
Philippines	LMIC	0.28	0.79	7.74 (7.43, 8.07)	<0.01	2009-2011	6 (3.87, 8.98)	<0.01	2011-2018	10.67 (10.03, 12.04)	<0.01	2018-2023	4.44 (3.17, 5.52)	<0.01
Singapore	HIC	2.29	2.09	-0.58 (-0.94, -0.13)	0.02	2009-2011	3.43 (-0.32, 7.38)	0.08	2011-2017	-4.69 (-6.54, -3.92)	<0.01	2017-2023	2.35 (1.34, 3.91)	<0.01
South Korea	HIC	4.56	13.19	7.56 (7.02, 8.13)	<0.01	2009-2018	9.05 (8.26, 10.69)	<0.01	2018-2023	4.91 (0.78, 6.75)	0.0344	–	–	–
Thailand	UMIC	1.56	3.01	4.84 (4.66, 5.03)	<0.01	2009-2012	3.72 (2.07, 4.9)	<0.01	2012-2019	6.33 (5.96, 7.06)	<0.01	2019-2023	3.11 (2.1, 3.88)	<0.01
Vietnam	LMIC	0.08	0.75	17.63 (16.24, 19.04)	<0.01	2009-2013	2.69 (-5.74, 8.15)	0.28	–	–	–	2013-2019	40.77 (35.97, 47.14)	<0.01
**Europe and Central Asia**														
Austria	HIC	7.00	5.95	-1.24 (-1.4, -1.09)	<0.01	2009-2011	-3.98 (-5.18, -2.73)	<0.01	2011-2018	-1.22 (-1.52, -0.91)	<0.01	2018-2023	-0.15 (-0.5, 0.72)	0.5611
Azerbaijan	UMIC	0.35	2.50	15.63 (14.88, 16.39)	<0.01	2009-2014	4.63 (2.51, 6.62)	<0.01	2014-2019	36.28 (33.51, 39.03)	<0.01	2019-2023	6.69 (3.84, 9.62)	<0.01
Belarus	UMIC	11.75	13.87	1.25 (1.12, 1.42)	<0.01	2009-2012	-1.14 (-2.34, -0.18)	0.02	2012-2016	0.24 (-0.46, 3.3)	0.226	2016-2023	2.88 (2.04, 3.67)	<0.01
Belgium	HIC	3.55	3.32	-0.64 (-0.95, -0.32)	<0.01	2009-2012	1.03 (-0.51, 4.11)	0.17	2012-2023	-1.09 (-1.88, -0.87)	<0.01	–	–	–
Bosnia and Herzegovina	UMIC	2.45	4.33	4.21 (3.94, 4.48)	<0.01	2009-2011	9.13 (6.67, 11.64)	<0.01	2011-2015	4.3 (2.87, 4.8)	<0.01	2015-2023	2.97 (0.87, 3.62)	<0.01
Bulgaria	UMIC	3.46	5.04	2.62 (0.5, 5.02)	0.04	2009-2013	10.95 (3.55, 35.69)	<0.01	2013-2023	-0.54 (-6.55, 1.2)	0.4627	–	–	–
Croatia	HIC	8.64	12.46	2.88 (2.53, 3.24)	<0.01	2009-2016	1.33 (-0.42, 2.14)	0.08	2016-2023	4.46 (3.63, 6.11)	<0.01	–	–	–
Czech Republic	HIC	6.90	7.75	0.92 (0.82, 1.02)	<0.01	2009-2012	1.03 (0.01, 1.6)	0.05	2012-2016	2.82 (2.25, 3.15)	<0.01	2016-2023	-0.2 (-0.41, -0.03)	0.022
Denmark	HIC	6.01	4.62	-1.89 (-2.11, -1.69)	<0.01	2009-2016	-4.21 (-4.91, -3.71)	<0.01	2016-2023	0.48 (-0.04, 1.19)	0.0644	–	–	–
Estonia	HIC	6.81	8.08	1.4 (1.03, 1.8)	<0.01	2009-2015	0.77 (-1.78, 3.44)	0.42	2015-2023	1.88 (-0.94, 4.28)	0.076	–	–	–

Finland	HIC	8.18	5.63	-2.6 (-2.72, -2.45)	<0.01	2009-2015	-2.31 (-2.6, -1.66)	<0.01	2015-2019	-3.92 (-4.33, -3.28)	<0.01	2019-2023	-1.71 (-2.25, -0.52)	0.0152
France	HIC	3.49	3.34	-0.33 (-0.39, -0.27)	<0.01	2009-2023	-0.33 (-0.39, -0.27)	<0.01	–	–	–	–	–	–
Georgia	UMIC	0.89	4.96	13.16 (11.94, 14.19)	<0.01	2009-2014	6.73 (0.76, 10.08)	0.03	2014-2020	21.59 (18.84, 27.08)	<0.01	2020-2023	8.06 (-1.3, 13.46)	0.0868
Germany	HIC	3.96	3.72	-0.4 (-0.51, -0.23)	<0.01	2009-2012	0.88 (0.21, 2.23)	<0.01	2012-2016	-1.41 (-1.75, -0.88)	<0.01	2016-2023	-0.37 (-0.62, 0.55)	0.2583
Greece	HIC	5.48	6.79	1.57 (1.4, 1.77)	<0.01	2009-2015	2.45 (2.01, 3.21)	<0.01	2015-2019	0.17 (-0.37, 1.12)	0.4051	2019-2023	1.68 (0.89, 3.23)	<0.01
Hungary	HIC	10.59	7.91	-2.26 (-2.59, -1.86)	<0.01	2009-2013	0.85 (-0.75, 3.4)	0.28	2013-2019	-4.86 (-6.44, -3.97)	<0.01	2019-2023	-1.36 (-2.8, 1.83)	0.2771
Ireland	HIC	8.77	8.56	-0.2 (-0.3, -0.08)	<0.01	2009-2012	-3.28 (-4.79, -2.57)	<0.01	2012-2023	0.66 (0.52, 0.8)	<0.01	–	–	–
Italy	HIC	4.71	3.86	-1.61 (-1.88, -1.34)	<0.01	2009-2014	-2.9 (-4.97, -2)	<0.01	2014-2023	-0.89 (-1.29, -0.1)	0.0388	–	–	–
Kazakhstan	UMIC	1.06	1.42	1.98 (1.17, 2.56)	<0.01	2009-2011	6.2 (1.44, 11.14)	<0.01	2011-2023	1.29 (-2.32, 2.05)	0.11	–	–	–
Latvia	HIC	9.00	11.85	1.67 (1.13, 2.26)	<0.01	2009-2014	8.71 (6.37, 11.61)	<0.01	2014-2023	-2.04 (-3.06, -1.14)	<0.01	–	–	–
Lithuania	HIC	7.04	9.28	1.97 (1.75, 2.19)	<0.01	2009-2017	3.15 (2.75, 3.69)	<0.01	2017-2023	0.42 (-0.37, 1.01)	0.2128	–	–	–
Netherlands	HIC	4.78	4.23	-0.88 (-0.99, -0.79)	<0.01	2009-2011	-0.67 (-1.43, -0.01)	0.05	2011-2015	-1.58 (-1.79, -0.35)	<0.01	2015-2023	-0.58 (-1.15, -0.23)	0.0156
North Macedonia	UMIC	2.06	2.77	2.11 (1.85, 2.42)	<0.01	2009-2011	10.32 (7.66, 12.8)	<0.01	2011-2015	-0.97 (-1.76, 0.27)	0.1424	2015-2023	1.69 (1.31, 2.62)	<0.01
Norway	HIC	8.96	6.27	-2.58 (-2.87, -2.29)	<0.01	2009-2019	-2.19 (-2.42, -1.18)	0.03	2019-2023	-3.56 (-6.13, -2.65)	<0.01	–	–	–
Poland	HIC	6.08	15.56	7.36 (6.97, 7.83)	<0.01	2009-2014	10.75 (9.27, 13.05)	<0.01	2014-2023	5.52 (4.74, 6.13)	<0.01	–	–	–
Portugal	HIC	2.48	4.62	4.56 (4.29, 4.86)	<0.01	2009-2012	4.52 (2.12, 6.48)	<0.01	2012-2016	7.53 (3.15, 8.44)	<0.01	2016-2023	2.91 (1.96, 3.61)	<0.01
Romania	HIC	0.95	4.15	11.66 (10.77, 12.7)	<0.01	2009-2012	22.32 (16.18, 34.44)	<0.01	2012-2023	8.91 (7.65, 9.89)	<0.01	–	–	–
Russia	UMIC	1.82	6.94	10 (9.76, 10.26)	<0.01	2009-2012	15.84 (14.2, 17.77)	<0.01	2012-2019	10.4 (9.8, 10.95)	<0.01	2019-2023	5.14 (3.88, 6.27)	<0.01
Serbia	UMIC	2.89	4.90	3.98 (3.81, 4.17)	<0.01	2009-2014	5.7 (5.03, 6.61)	<0.01	2014-2023	3.04 (2.72, 3.32)	<0.01	–	–	–
Slovakia	HIC	8.95	10.00	0.6 (0.19, 1.23)	<0.01	2009-2011	-2.61 (-5.71, 2.8)	0.20	2011-2015	3.95 (-0.93, 5.53)	0.0788	2015-2023	-0.23 (-1.62, 1.31)	0.3559
Slovenia	HIC	7.32	7.99	0.67 (0.55, 0.79)	<0.01	2009-2023	0.67 (0.55, 0.79)	<0.01	–	–	–	–	–	–
Spain	HIC	5.19	6.00	1.06 (0.94, 1.18)	<0.01	2009-2012	-1.05 (-2.55, -0.31)	0.014	2012-2018	2.35 (2.06, 2.91)	<0.01	2018-2023	0.81 (0.28, 1.16)	0.0148
Sweden	HIC	5.45	4.54	-1.2 (-1.36, -1)	<0.01	2009-2011	-3.45 (-4.31, -1.84)	<0.01	2011-2019	-1.48 (-1.87, -0.73)	<0.01	2019-2023	0.5 (-0.24, 2.15)	0.1404
Switzerland	HIC	7.25	6.44	-0.89 (-1.09, -0.73)	<0.01	2009-2013	-2.06 (-3.96, -1.31)	<0.01	2013-2017	0.65 (-0.11, 1.22)	0.0924	2017-2023	-1.13 (-1.97, -0.84)	<0.01
Turkey	UMIC	2.77	3.89	2.67 (2.37, 2.99)	<0.01	2009-2014	5.25 (4.16, 6.48)	<0.01	2014-2019	-0.47 (-1.74, 0.54)	0.2987	2019-2023	3.48 (2.16, 6.65)	<0.01
Ukraine	LMIC	1.39	1.69	1.9 (0.96, 2.79)	<0.01	2009-2013	4.91 (2.05, 13.08)	<0.01	2013-2023	0.71 (-3.69, 1.48)	0.3123	–	–	–
United Kingdom	HIC	5.10	5.59	0.64 (0.55, 0.74)	<0.01	2009-2011	4.09 (3.02, 5.01)	<0.01	2011-2015	0.82 (0.25, 1.09)	0.016	2015-2023	-0.29 (-0.53, -0.17)	<0.01
Uzbekistan	LMIC	0.03	0.17	15.07 (14.06, 16.07)	<0.01	2009-2014	3.95 (-2.16, 6.63)	0.18	2014-2018	10.72 (5.98, 27.12)	<0.01	2018-2023	31.36 (27.77, 38.66)	<0.01
**Latin America and Caribbean**														
Argentina	UMIC	1.14	1.26	0.63 (0.41, 0.86)	<0.01	2009-2023	0.63 (0.41, 0.86)	<0.01	–	–	–	–	–	–

Bolivia	LMIC	0.06	0.09	2.93 (2.44, 3.39)	<0.01	2009-2015	7.72 (5.59, 10.09)	<0.01	2015-2019	1.23 (-0.68, 8.53)	0.1608	2019-2023	-2.24 (-6.06, -0.52)	0.0136
Brazil	UMIC	0.46	0.63	2.09 (1.78, 2.36)	<0.01	2009-2011	5.41 (3.41, 7.71)	<0.01	2011-2016	2.84 (-0.12, 3.36)	0.0644	2016-2023	0.64 (-1.24, 1.84)	0.1104
Chile	HIC	0.93	1.24	2.16 (1.88, 2.46)	<0.01	2009-2013	7.47 (5.86, 8.78)	<0.01	2013-2019	-0.68 (-1.74, 0)	0.0512	2019-2023	1.29 (0.21, 3.64)	0.0196
Colombia	UMIC	0.57	0.72	1.59 (1.36, 1.81)	<0.01	2009-2023	1.59 (1.36, 1.81)	<0.01	–	–	–	–	–	–
Costa Rica	UMIC	0.94	1.47	3.24 (2.98, 3.49)	<0.01	2009-2017	5.18 (4.65, 5.77)	<0.01	2017-2023	0.71 (-0.18, 1.46)	0.0908	–	–	–
Dominican Republic	UMIC	0.38	0.47	1.54 (1.4, 1.69)	<0.01	2009-2013	4.33 (3.39, 5.07)	<0.01	2013-2023	0.44 (0.22, 0.63)	<0.01	–	–	–
Ecuador	UMIC	0.24	0.38	3.36 (3.11, 3.62)	<0.01	2009-2016	5.44 (4.82, 6.17)	<0.01	2016-2023	1.32 (0.64, 1.93)	<0.01	–	–	–
El Salvador	LMIC	0.48	0.74	3.14 (2.89, 3.37)	<0.01	2009-2017	4.9 (4.4, 5.44)	<0.01	2017-2023	0.85 (0.03, 1.54)	0.0456	–	–	–
Guatemala	UMIC	0.24	0.54	6.2 (5.37, 7.01)	<0.01	2009-2017	3.79 (-0.05, 5.32)	0.05	2017-2023	9.5 (7.16, 15.72)	<0.01	–	–	–
Honduras	LMIC	0.38	0.50	1.9 (1.7, 2.11)	<0.01	2009-2012	7.88 (6.3, 9.31)	<0.01	2012-2017	1.58 (0.96, 2.39)	<0.01	2017-2023	-0.71 (-1.39, -0.28)	<0.01
Mexico	UMIC	1.42	2.52	4.04 (3.75, 4.42)	<0.01	2009-2013	6.68 (5.27, 9.26)	<0.01	2013-2023	3.01 (2.5, 3.38)	<0.01	–	–	–
Panama	HIC	0.34	0.53	3.17 (2.97, 3.41)	<0.01	2009-2011	0.98 (-0.53, 3.29)	0.18	2011-2016	8.4 (7.87, 9.2)	<0.01	2016-2023	0.2 (-0.18, 0.57)	0.2643
Paraguay	UMIC	1.26	1.77	2.24 (1.83, 2.63)	<0.01	2009-2023	2.24 (1.83, 2.63)	<0.01	–	–	–	–	–	–
Peru	UMIC	0.15	0.39	6.39 (5.68, 7.02)	<0.01	2009-2019	9.52 (8.61, 10.59)	<0.01	2019-2023	-1.04 (-5.58, 1.98)	0.4571	–	–	–
Uruguay	HIC	2.20	2.69	1.45 (1.36, 1.53)	<0.01	2009-2013	1.14 (0.22, 1.49)	0.02	2013-2017	2.54 (2.1, 2.82)	<0.01	2017-2023	0.93 (0.64, 1.12)	<0.01
**Middle East and North Africa**														
Algeria	LMIC	0.05	0.08	3.49 (3.4, 3.6)	<0.01	2009-2011	2.52 (1.77, 3.4)	<0.01	2011-2015	5.85 (5.39, 6.2)	<0.01	2015-2023	2.58 (2.4, 2.72)	<0.01
Egypt	LMIC	0.32	0.33	0.26 (0.16, 0.4)	<0.01	2009-2012	0.06 (-0.48, 1.3)	0.67	2012-2016	-1.8 (-2.16, -1.24)	0.018	2016-2023	1.56 (1.31, 1.89)	<0.01
Iraq	UMIC	0.14	0.15	0.51 (0.23, 0.71)	<0.01	2009-2014	-0.4 (-2.35, 0.3)	0.19	2014-2018	2.56 (1.49, 3.28)	<0.01	2018-2023	-0.2 (-1.81, 0.37)	0.4183
Israel	HIC	1.71	1.67	-0.22 (-0.34, -0.1)	<0.01	2009-2012	1.56 (0.87, 2.65)	<0.01	2012-2017	0.06 (-0.78, 0.48)	0.871	2017-2023	-1.33 (-1.87, -1.06)	<0.01
Jordan	UMIC	0.65	1.02	3.18 (2.89, 3.57)	<0.01	2009-2012	12.7 (10.65, 15.05)	<0.01	2012-2016	-1.38 (-2.31, 0.21)	0.084	2016-2023	1.96 (1.39, 3.74)	<0.01
Kuwait	HIC	0.60	0.69	0.82 (0.38, 1.52)	<0.01	2009-2012	-1.98 (-5.74, 1.37)	0.35	2012-2023	1.6 (-2.27, 4.58)	0.0624	–	–	–
Lebanon	LMIC	0.28	0.46	3.46 (2.86, 4.2)	<0.01	2009-2014	0.35 (-5.56, 2.79)	0.80	2014-2023	5.23 (4.21, 8.32)	<0.01	–	–	–
Morocco	LMIC	0.32	0.35	0.71 (0.53, 0.92)	<0.01	2009-2013	-1.93 (-3.61, -0.95)	<0.01	2013-2023	1.78 (1.5, 2.14)	<0.01	–	–	–
Oman	HIC	1.59	1.26	-1.87 (-2.34, -1.25)	<0.01	2009-2013	-5.46 (-10.42, -2.75)	<0.01	2013-2023	-0.4 (-1.09, 1.21)	0.4107	–	–	–
Qatar	HIC	1.27	1.95	2.85 (2.42, 3.55)	<0.01	2009-2012	-1.84 (-6.2, 2.11)	0.29	2012-2023	4.16 (3.56, 5.51)	<0.01	–	–	–
Saudi Arabia	HIC	1.42	1.65	0.75 (0.24, 1.32)	0.01	2009-2015	2.94 (1.72, 5.37)	<0.01	2015-2023	-0.87 (-2.2, -0.09)	0.0312	–	–	–
Tunisia	LMIC	0.15	0.35	6.56 (6.15, 7.02)	<0.01	2009-2015	10.02 (8.83, 11.75)	<0.01	2015-2023	4.04 (3.04, 4.82)	<0.01	–	–	–
United Arab Emirates	HIC	1.09	1.75	3.29 (2.6, 3.77)	<0.01	2009-2013	-2.66 (-6.02, -0.46)	0.02	2013-2017	9.42 (6.49, 11.08)	<0.01	2017-2023	3.4 (-1.29, 4.71)	0.1056
**North America**														

Canada	HIC	4.00	3.90	-0.2 (-0.39, -0.02)	0.03	2009-2013	1.09 (0.5, 2.63)	0.01	2013-2018	-0.22 (-1.17, 0.42)	0.3951	2018-2023	-1.21 (-2.73, -0.56)	0.0132
USA	HIC	5.70	5.28	-0.55 (-0.67, -0.42)	<0.01	2009-2017	-0.37 (-0.51, 0.45)	0.10	2017-2023	-0.78 (-1.72, -0.57)	<0.01	–	–	–
**South Asia**														
Bangladesh	LMIC	0.02	0.04	3.67 (3.46, 3.91)	<0.01	2009-2012	8.86 (6.92, 10.16)	<0.01	2012-2023	2.3 (2.03, 2.53)	<0.01	–	–	–
India	LMIC	0.10	0.15	2.86 (2.51, 3.16)	<0.01	2009-2018	3.62 (3.23, 4.4)	<0.01	2018-2023	1.5 (-0.77, 2.45)	0.0884	–	–	–
Pakistan	LMIC	0.04	0.06	3.38 (2.99, 3.74)	<0.01	2009-2017	1.1 (-0.12, 2.42)	0.06	2017-2021	8.09 (0.51, 9.05)	0.0268	2021-2023	3.39 (0.5, 6.86)	0.016
Sri Lanka	LMIC	0.18	0.94	12.18 (11.63, 12.72)	<0.01	2009-2017	20.11 (19.02, 21.28)	<0.01	2017-2023	2.42 (1.03, 3.82)	<0.01	–	–	–
**Sub-Saharan Africa**														
Angola	LMIC	0.56	0.51	-0.8 (-0.95, -0.64)	<0.01	2009-2013	2.38 (1.41, 3.1)	<0.01	2013-2023	-2.04 (-2.27, -1.84)	<0.01	–	–	–
Cameroon	LMIC	0.24	0.31	1.59 (1.36, 1.8)	<0.01	2009-2018	3.28 (2.9, 3.69)	<0.01	2018-2023	-1.39 (-2.37, -0.52)	<0.01	–	–	–
Côte d'Ivoire	LMIC	0.08	0.17	5.09 (4.77, 5.48)	<0.01	2009-2011	-0.19 (-2.39, 3.47)	0.89	2011-2017	9.76 (8.81, 11.2)	<0.01	2017-2023	2.35 (1.4, 3.28)	<0.01
Ethiopia	LMIC	0.01	0.01	1.39 (1, 1.7)	<0.01	2009-2014	0.82 (-2.35, 1.88)	0.39	2014-2018	4.96 (3.27, 6.05)	<0.01	2018-2023	-0.81 (-2.61, 0.15)	0.0768
Ghana	LMIC	0.13	0.25	4.9 (4.41, 5.33)	<0.01	2009-2018	7.63 (6.89, 8.51)	<0.01	2018-2023	0.15 (-1.81, 1.76)	0.8002	–	–	–
Kenya	LMIC	0.09	0.12	2.26 (1.93, 2.61)	<0.01	2009-2015	6.87 (5.65, 7.96)	<0.01	2015-2023	-1.06 (-1.74, -0.34)	<0.01	–	–	–
Nigeria	LMIC	0.05	0.04	-1.41 (-1.95, -0.92)	<0.01	2009-2016	4.87 (3.91, 6.08)	<0.01	2016-2020	-11.14 (-12.62, -8.61)	<0.01	2020-2023	-1.95 (-4.84, 3.7)	0.3219
South Africa	UMIC	1.17	1.27	0.54 (0.4, 0.7)	<0.01	2009-2012	3.62 (2.77, 5.12)	<0.01	2012-2017	1.18 (0.38, 1.73)	0.0152	2017-2023	-1.49 (-1.97, -1.12)	<0.01
Tanzania	LMIC	0.12	0.17	2.57 (2.29, 2.91)	<0.01	2009-2012	5.04 (3.27, 8.47)	<0.01	2012-2023	1.9 (1.45, 2.19)	<0.01	–	–	–
Uganda	LMIC	0.13	0.20	3.65 (3.23, 4.06)	<0.01	2009-2016	7.1 (6.04, 8.39)	<0.01	2016-2023	0.3 (-0.86, 1.27)	0.5255	–	–	–

Legend: Annual Average Percentage Change (AAPC), Annual Percentage Change (APC) and 95% Confidence Interval (CI) estimated using joinpoint regression models are shown. Original units and the description for the used indicator are reported in Supplementary Table 3. The results represent the percentage change in the density of chain outlets for each identified segment and for the entire analysis period (2009 to 2023), by country. Crude metrics for the first (2009) and last (2023) time points are reported. Two-tailed t-test was used to test whether AAPC and APC was statistically different from zero, with no adjustment made for multiple comparisons. LMICs=Low-and-middle-income countries; UMICs=Upper-and-middle-income countries; HICs=High-income countries.

Supplementary Table 6. Density of non-chain food retail outlets per 10,000 population from 2009 to 2023, by country: joinpoint regression analysis.

	Income status	Density of non-chain outlets		AAPC		Segment 1			Segment 2			Segment 3		
		2009	2023	AAPC (95% CI)	p-value	Years	APC (95% CI)	p-value	Years	APC (95% CI)	p-value	Years	APC (95% CI)	p-value
**East Asia and Pacific**														
Australia	HIC	4.9	3.6	-2.06 (-2.18, -1.91)	<0.01	2009-2011	-1.99 (-2.99, -0.9)	<0.01	2011-2018	-3.65 (-4.09, -3.46)	<0.01	2018-2023	0.18 (-0.18, 0.59)	0.25
Cambodia	LMIC	67.8	65.7	-0.29 (-0.37, -0.21)	<0.01	2009-2018	-0.17 (-0.26, 0.23)	0.15	2018-2023	-0.51 (-1.11, -0.31)	<0.01	–	–	–
China	UMIC	86.6	69.4	-1.66 (-1.85, -1.48)	<0.01	2009-2016	-0.92 (-1.3, -0.29)	0.02	2016-2023	-2.4 (-3.02, -2.04)	<0.01	–	–	–
Indonesia	LMIC	189.7	141.7	-2.3 (-2.64, -1.98)	<0.01	2009-2018	-1.28 (-1.75, -0.55)	0.01	2018-2023	-4.11 (-6.14, -3.09)	<0.01	–	–	–
Japan	HIC	9.6	7.9	-1.35 (-1.36, -1.33)	<0.01	2009-2011	-1.69 (-1.8, -1.52)	<0.01	2011-2017	-1.46 (-1.5, -1.18)	<0.01	2017-2023	-1.12 (-1.16, -1.05)	<0.01
Laos	LMIC	69.7	67.4	-0.33 (-0.41, -0.24)	<0.01	2009-2017	-0.15 (-0.27, 0.19)	0.20	2017-2023	-0.56 (-1.11, -0.38)	<0.01	–	–	–
Malaysia	UMIC	14.0	8.8	-3.27 (-3.56, -2.98)	<0.01	2009-2018	-2.71 (-3.05, -1.73)	0.02	2018-2023	-4.25 (-6.43, -3.46)	<0.01	–	–	–
Myanmar	LMIC	33.4	37.4	0.57 (-0.1, 1.32)	0.08	2009-2018	1.34 (0.74, 6.38)	0.01	2018-2023	-0.79 (-6.05, 0.51)	0.2695	–	–	–
New Zealand	HIC	7.2	5.5	-1.9 (-2, -1.83)	<0.01	2009-2014	-0.99 (-1.29, -0.72)	<0.01	2014-2021	-2.64 (-2.97, -2.48)	<0.01	2021-2023	-1.57 (-2.41, -1.09)	<0.01
Philippines	LMIC	117.1	121.7	0.29 (0.15, 0.41)	<0.01	2009-2019	-1.04 (-1.22, -0.85)	<0.01	2019-2023	3.69 (3.07, 4.25)	<0.01	–	–	–
Singapore	HIC	2.9	2.4	-1.16 (-1.35, -0.96)	<0.01	2009-2014	-0.84 (-1.29, 0.6)	0.12	2014-2020	-2.13 (-2.94, -1.75)	<0.01	2020-2023	0.24 (-0.92, 2.07)	0.63
South Korea	HIC	19.7	12.3	-3.2 (-3.53, -2.72)	<0.01	2009-2014	-3.34 (-4.47, 0.18)	0.06	2014-2018	-5.86 (-7.06, -4.18)	<0.01	2018-2023	-0.87 (-2.12, 2.53)	0.40
Thailand	UMIC	81.1	80.1	-0.09 (-0.29, 0.05)	0.20	2009-2017	0.16 (-0.58, 0.75)	0.12	2017-2021	-1.06 (-1.51, 0.48)	0.1172	2021-2023	0.86 (-0.74, 1.92)	0.26
Vietnam	LMIC	69.5	64.7	-0.67 (-0.87, -0.49)	<0.01	2009-2019	-0.15 (-0.37, 0.18)	0.33	2019-2023	-1.96 (-3.42, -1.17)	<0.01	–	–	–
**Europe and Central Asia**														
Austria	HIC	1.9	1.6	-1.28 (-1.37, -1.18)	<0.01	2009-2023	-1.28 (-1.37, -1.18)	<0.01	–	–	–	–	–	–
Azerbaijan	UMIC	49.7	36.9	-2.13 (-2.2, -2.05)	<0.01	2009-2012	-2.64 (-2.97, -1.91)	<0.01	2012-2016	-3.65 (-3.85, -3.33)	<0.01	2016-2023	-1.04 (-1.18, -0.88)	<0.01
Belarus	UMIC	12.5	9.8	-1.99 (-2.39, -1.51)	<0.01	2009-2011	9.18 (4.73, 13.65)	<0.01	2011-2016	2.72 (-6.61, 3.56)	0.1236	2016-2023	-8.11 (-8.97, -7.27)	<0.01
Belgium	HIC	6.8	4.8	-2.5 (-2.58, -2.41)	<0.01	2009-2011	-3.23 (-3.7, -2.43)	<0.01	2011-2018	-2.12 (-2.25, -1.73)	<0.01	2018-2023	-2.74 (-3.18, -2.54)	<0.01
Bosnia and Herzegovina	UMIC	14.3	13.2	-0.57 (-0.78, -0.37)	<0.01	2009-2012	-3.33 (-5.51, -2.09)	<0.01	2012-2016	1.03 (0.33, 1.72)	<0.01	2016-2023	-0.27 (-1.42, -0.04)	0.03
Bulgaria	UMIC	46.4	44.6	-0.21 (-0.43, 0.02)	0.07	2009-2023	-0.21 (-0.43, 0.02)	0.07	–	–	–	–	–	–
Croatia	HIC	28.4	20.4	-2.27 (-2.47, -2.11)	<0.01	2009-2014	-5.41 (-6.57, -4.87)	<0.01	2014-2020	-1.1 (-3.31, -0.58)	<0.01	2020-2023	0.75 (-0.44, 2.3)	0.16
Czech Republic	HIC	11.1	8.5	-1.85 (-1.93, -1.78)	<0.01	2009-2019	-2.42 (-2.54, -2.32)	<0.01	2019-2023	-0.41 (-0.8, 0.23)	0.0844	–	–	–
Denmark	HIC	3.2	2.2	-2.71 (-2.88, -2.53)	<0.01	2009-2015	-5.48 (-6.14, -4.95)	<0.01	2015-2023	-0.58 (-0.95, -0.15)	0.0176	–	–	–
Estonia	HIC	9.1	5.6	-3.5 (-3.65, -3.35)	<0.01	2009-2011	-3.07 (-4.4, -1.91)	<0.01	2011-2016	-5.4 (-6.04, -4.87)	<0.01	2016-2023	-2.24 (-2.58, -1.84)	<0.01

Finland	HIC	3.2	2.6	-1.38 (-1.47, -1.25)	<0.01	2009-2012	-3.31 (-4.7, -2.51)	<0.01	2012-2023	-0.84 (-0.96, -0.68)	<0.01	–	–	–
France	HIC	3.8	3.6	-0.41 (-0.55, -0.29)	<0.01	2009-2014	-0.76 (-1.62, -0.37)	<0.01	2014-2018	1.5 (0.76, 1.91)	<0.01	2018-2023	-1.57 (-2.11, -1.21)	<0.01
Georgia	UMIC	49.7	44.2	-0.79 (-0.94, -0.67)	<0.01	2009-2012	-0.44 (-0.89, 0.6)	0.13	2012-2023	-0.89 (-1.82, -0.71)	<0.01	–	–	–
Germany	HIC	3.8	3.1	-1.57 (-1.68, -1.47)	<0.01	2009-2011	0.96 (-0.19, 1.81)	0.09	2011-2016	-2.91 (-3.35, -2.56)	<0.01	2016-2023	-1.33 (-1.55, -1.05)	<0.01
Greece	HIC	32.3	25.7	-1.63 (-1.69, -1.57)	<0.01	2009-2013	-3.79 (-4.04, -3.55)	<0.01	2013-2023	-0.75 (-0.84, -0.67)	<0.01	–	–	–
Hungary	HIC	19.9	12.3	-3.27 (-3.41, -3.06)	<0.01	2009-2011	-5.89 (-6.92, -4.11)	<0.01	2011-2016	-3.43 (-3.91, -2.26)	<0.01	2016-2023	-2.4 (-3.33, -1.31)	<0.01
Ireland	HIC	6.4	3.8	-3.72 (-3.81, -3.66)	<0.01	2009-2017	-5.01 (-5.15, -4.89)	<0.01	2017-2021	-2.95 (-3.41, -2.72)	<0.01	2021-2023	-0.04 (-0.79, 0.53)	0.90
Italy	HIC	20.3	15.4	-2.06 (-2.34, -1.74)	<0.01	2009-2020	-1.76 (-2.02, -0.27)	0.04	2020-2023	-3.15 (-5.59, -1.97)	<0.01	–	–	–
Kazakhstan	UMIC	28.8	17.2	-3.84 (-4.68, -2.98)	<0.01	2009-2016	-1.72 (-3.13, 1.92)	0.16	2016-2023	-5.91 (-9.56, -4.57)	<0.01	–	–	–
Latvia	HIC	16.6	12.4	-1.94 (-2.07, -1.81)	<0.01	2009-2017	-2.8 (-3.13, -2.54)	<0.01	2017-2023	-0.78 (-1.17, -0.22)	0.014	–	–	–
Lithuania	HIC	11.8	10.5	-0.82 (-0.98, -0.68)	<0.01	2009-2013	-2.56 (-3.59, -1.95)	<0.01	2013-2019	0.49 (0.14, 1.18)	<0.01	2019-2023	-1 (-2.09, -0.5)	<0.01
Netherlands	HIC	7.3	5.7	-1.8 (-1.92, -1.7)	<0.01	2009-2015	-1.69 (-2.19, -1.44)	<0.01	2015-2019	0.14 (-0.46, 0.48)	0.6307	2019-2023	-3.86 (-4.34, -3.44)	<0.01
North Macedonia	UMIC	38.5	36.5	-0.38 (-0.41, -0.35)	<0.01	2009-2011	-1.26 (-1.44, -0.97)	<0.01	2011-2018	-0.41 (-0.48, -0.33)	<0.01	2018-2023	0.01 (-0.08, 0.15)	0.86
Norway	HIC	1.5	1.3	-1.42 (-1.56, -1.27)	<0.01	2009-2012	1.29 (0.28, 2.87)	0.01	2012-2019	-2.44 (-3, -2.22)	<0.01	2019-2023	-1.63 (-2.09, -0.57)	<0.01
Poland	HIC	29.1	0.6	-25.13 (-25.93, -24.33)	<0.01	2009-2016	-10.03 (-12.23, -7.67)	<0.01	2016-2023	-37.69 (-39.27, -36.12)	<0.01	–	–	–
Portugal	HIC	14.8	10.0	-2.8 (-2.89, -2.71)	<0.01	2009-2014	-4.21 (-4.68, -3.93)	<0.01	2014-2019	-1.67 (-1.93, -1.28)	<0.01	2019-2023	-2.41 (-3.23, -2.14)	<0.01
Romania	HIC	29.1	18.5	-3.22 (-3.29, -3.14)	<0.01	2009-2013	-6.47 (-6.8, -6.19)	<0.01	2013-2017	-3.45 (-3.73, -3.15)	<0.01	2017-2023	-0.83 (-1.01, -0.62)	<0.01
Russia	UMIC	19.7	8.1	-6.55 (-7.05, -6.14)	<0.01	2009-2019	-4.26 (-4.88, -3.56)	<0.01	2019-2023	-12.04 (-15.06, -9.95)	<0.01	–	–	–
Serbia	UMIC	36.6	31.3	-1.1 (-1.14, -1.06)	<0.01	2009-2023	-1.1 (-1.14, -1.06)	<0.01	–	–	–	–	–	–
Slovakia	HIC	7.5	4.5	-3.33 (-3.65, -2.78)	<0.01	2009-2011	-13.16 (-15.27, -8.55)	<0.01	2011-2023	-1.58 (-1.99, -1.09)	<0.01	–	–	–
Slovenia	HIC	7.0	4.5	-3.09 (-3.21, -2.95)	<0.01	2009-2012	-5.33 (-6.43, -4.5)	<0.01	2012-2017	-3.59 (-4.17, -2.39)	<0.01	2017-2023	-1.53 (-1.85, -0.88)	<0.01
Spain	HIC	6.6	5.0	-1.94 (-2.13, -1.76)	<0.01	2009-2017	-0.8 (-1.15, -0.36)	<0.01	2017-2023	-3.44 (-4.09, -2.94)	<0.01	–	–	–
Sweden	HIC	2.5	1.7	-2.72 (-2.79, -2.64)	<0.01	2009-2017	-2.21 (-2.32, -2.06)	<0.01	2017-2021	-4.02 (-4.24, -3.67)	<0.01	2021-2023	-2.12 (-2.78, -1.54)	<0.01
Switzerland	HIC	4.6	3.2	-2.43 (-2.6, -2.26)	<0.01	2009-2016	-3.67 (-4.3, -3.26)	<0.01	2016-2023	-1.17 (-1.6, -0.55)	<0.01	–	–	–
Turkey	UMIC	30.7	20.5	-2.86 (-2.99, -2.73)	<0.01	2009-2014	-1.64 (-2.13, -0.97)	<0.01	2014-2023	-3.54 (-3.78, -3.33)	<0.01	–	–	–
Ukraine	LMIC	31.6	14.8	-5.2 (-5.9, -4.56)	<0.01	2009-2014	-11.07 (-14.55, -8.85)	<0.01	2014-2023	-1.78 (-2.87, -0.38)	0.0232	–	–	–
United Kingdom	HIC	0.5	0.3	-3.51 (-3.57, -3.47)	<0.01	2009-2017	-5.02 (-5.1, -4.94)	<0.01	2017-2021	-2.04 (-2.21, -1.88)	<0.01	2021-2023	-0.3 (-0.78, 0.09)	0.13
Uzbekistan	LMIC	15.8	17.0	0.63 (0.36, 0.89)	<0.01	2009-2023	0.63 (0.36, 0.89)	<0.01	–	–	–	–	–	–
**Latin America and Caribbean**														
Argentina	UMIC	60.7	52.4	-1.09 (-1.19, -0.96)	<0.01	2009-2011	-0.27 (-1.06, 0.79)	0.31	2011-2018	-1.52 (-2, -1.37)	<0.01	2018-2023	-0.83 (-1.16, 0.02)	0.05

Bolivia	LMIC	124.3	105.0	-1.21 (-1.3, -1.16)	<0.01	2009-2021	-1.34 (-1.5, -1.28)	<0.01	2021-2023	-0.41 (-1.19, -0.07)	0.0248	–	–	–
Brazil	UMIC	14.6	13.7	-0.49 (-0.76, -0.19)	<0.01	2009-2013	4.27 (3.03, 5.76)	<0.01	2013-2017	-5.78 (-6.63, -4.39)	<0.01	2017-2023	0.03 (-0.57, 1.05)	0.82
Chile	HIC	118.8	90.1	-1.98 (-2.03, -1.92)	<0.01	2009-2012	-2.56 (-2.9, -2.21)	<0.01	2012-2020	-2.29 (-2.42, -2.11)	<0.01	2020-2023	-0.53 (-0.86, 0.21)	0.08
Colombia	UMIC	94.8	97.2	0.09 (0.02, 0.16)	0.02	2009-2017	0.79 (0.64, 0.95)	<0.01	2017-2023	-0.83 (-1.09, -0.6)	<0.01	–	–	–
Costa Rica	UMIC	43.8	31.2	-2.7 (-3.4, -2.2)	<0.01	2009-2021	-1.04 (-1.51, -0.38)	<0.01	2021-2023	-12.07 (-16.82, -6.72)	<0.01	–	–	–
Dominican Republic	UMIC	68.1	60.0	-0.92 (-0.95, -0.9)	<0.01	2009-2016	-1.08 (-1.24, -1)	<0.01	2016-2021	-0.91 (-1.06, -0.82)	<0.01	2021-2023	-0.39 (-0.69, -0.22)	<0.01
Ecuador	UMIC	54.3	44.7	-1.42 (-1.52, -1.34)	<0.01	2009-2020	-1.88 (-2, -1.79)	<0.01	2020-2023	0.3 (-0.36, 1.49)	0.2404	–	–	–
El Salvador	LMIC	18.5	15.9	-1.2 (-1.35, -1.03)	<0.01	2009-2013	-2.41 (-3.92, -1.7)	<0.01	2013-2023	-0.7 (-0.9, -0.38)	<0.01	–	–	–
Guatemala	UMIC	81.7	81.0	-0.08 (-0.14, -0.02)	0.01	2009-2012	1.9 (1.59, 2.21)	<0.01	2012-2018	-0.45 (-0.56, -0.24)	0.0132	2018-2023	-0.8 (-1.25, -0.67)	<0.01
Honduras	LMIC	32.5	23.9	-2.23 (-2.32, -2.13)	<0.01	2009-2023	-2.23 (-2.32, -2.13)	<0.01	–	–	–	–	–	–
Mexico	UMIC	50.8	63.9	1.92 (0.81, 2.96)	<0.01	2009-2017	4.62 (3.02, 7.92)	<0.01	2017-2023	-1.57 (-6.8, 0.69)	0.1624	–	–	–
Panama	HIC	30.4	25.7	-1.24 (-1.3, -1.18)	<0.01	2009-2023	-1.24 (-1.3, -1.18)	<0.01	–	–	–	–	–	–
Paraguay	UMIC	82.9	81.1	-0.26 (-0.48, -0.06)	0.02	2009-2011	2.29 (0.67, 4.09)	<0.01	2011-2017	0 (-1.12, 0.36)	0.8078	2017-2023	-1.35 (-2.63, -0.89)	0.02
Peru	UMIC	124.0	113.1	-0.64 (-0.69, -0.57)	<0.01	2009-2015	-0.88 (-1.01, -0.48)	<0.01	2015-2019	-1.55 (-1.74, -1.25)	<0.01	2019-2023	0.65 (0.39, 0.95)	<0.01
Uruguay	HIC	52.6	66.4	1.66 (1.51, 1.8)	<0.01	2009-2012	7.31 (6.59, 8.13)	<0.01	2012-2018	0.61 (0.32, 1.07)	<0.01	2018-2023	-0.34 (-1.37, -0.02)	0.03
**Middle East and North Africa**														
Algeria	LMIC	40.8	39.2	-0.34 (-0.41, -0.29)	<0.01	2009-2012	-0.57 (-1.09, -0.28)	<0.01	2012-2017	0.13 (-0.05, 0.37)	0.1532	2017-2023	-0.62 (-0.86, -0.48)	<0.01
Egypt	LMIC	11.4	9.4	-1.32 (-1.72, -0.95)	<0.01	2009-2015	-2.54 (-5.1, -1.69)	<0.01	2015-2023	-0.4 (-0.98, 1.38)	0.4047	–	–	–
Iraq	UMIC	13.8	8.8	-3.25 (-3.45, -3.06)	<0.01	2009-2015	-4.65 (-5.55, -4.07)	<0.01	2015-2023	-2.19 (-2.57, -1.65)	<0.01	–	–	–
Israel	HIC	10.2	5.2	-4.75 (-5.03, -4.51)	<0.01	2009-2016	-8.05 (-8.69, -7.55)	<0.01	2016-2021	-0.07 (-0.97, 0.89)	0.9898	2021-2023	-4.39 (-6.54, -2.08)	<0.01
Jordan	UMIC	31.4	20.5	-2.95 (-3.17, -2.67)	<0.01	2009-2012	-0.08 (-1.49, 2.37)	0.93	2012-2016	-6.37 (-7.06, -5.15)	<0.01	2016-2023	-2.17 (-2.6, -1.45)	<0.01
Kuwait	HIC	2.0	0.9	-5.3 (-5.56, -5.08)	<0.01	2009-2014	-7.41 (-8.82, -6.73)	<0.01	2014-2021	-5.25 (-5.89, -4.61)	<0.01	2021-2023	0.02 (-2.55, 1.69)	0.93
Lebanon	LMIC	9.7	7.1	-2.03 (-2.36, -1.7)	<0.01	2009-2011	1.13 (-1.61, 3.82)	0.40	2011-2015	-10 (-10.94, -8.96)	<0.01	2015-2023	1.41 (0.92, 1.97)	<0.01
Morocco	LMIC	61.3	54.3	-0.86 (-0.89, -0.84)	<0.01	2009-2016	-1.06 (-1.18, -1.01)	<0.01	2016-2021	-0.8 (-0.99, -0.71)	<0.01	2021-2023	-0.34 (-0.61, -0.18)	<0.01
Oman	HIC	2.7	1.1	-6.42 (-6.67, -6.16)	<0.01	2009-2012	-15.38 (-17.91, -14.05)	<0.01	2012-2016	-8.15 (-9.26, -6.17)	<0.01	2016-2023	-1.25 (-1.76, -0.55)	<0.01
Qatar	HIC	5.5	4.0	-2.23 (-2.54, -1.78)	<0.01	2009-2011	-6.96 (-8.92, -3.48)	<0.01	2011-2018	-2.8 (-3.85, -0.67)	0.024	2018-2023	0.56 (-0.76, 3.69)	0.28
Saudi Arabia	HIC	7.4	6.0	-1.37 (-1.51, -1.23)	<0.01	2009-2016	-0.42 (-0.69, -0.05)	0.03	2016-2020	-3.98 (-4.42, -3.2)	<0.01	2020-2023	-0.05 (-0.9, 1.64)	0.95
Tunisia	LMIC	63.5	61.5	-0.25 (-0.28, -0.22)	<0.01	2009-2012	-0.81 (-1.07, -0.64)	<0.01	2012-2018	-0.32 (-0.4, -0.22)	<0.01	2018-2023	0.17 (0.08, 0.29)	<0.01
United Arab Emirates	HIC	5.8	3.7	-3.15 (-3.34, -2.82)	<0.01	2009-2011	-9.13 (-10.81, -6.35)	<0.01	2011-2015	-4.24 (-4.78, -0.82)	<0.01	2015-2023	-1.04 (-1.5, 0.05)	0.06
**North America**														

Canada	HIC	6.6	5.5	-1.28 (-1.38, -1.18)	<0.01	2009-2023	-1.28 (-1.38, -1.18)	<0.01	–	–	–	–	–	–
USA	HIC	2.9	3.8	1.82 (1.54, 2.14)	<0.01	2009-2012	10.21 (7.27, 12.09)	<0.01	2012-2023	-0.35 (-0.71, -0.03)	0.0308	–	–	–
**South Asia**														
Bangladesh	LMIC	34.7	31.5	-0.72 (-0.78, -0.69)	<0.01	2009-2021	-0.85 (-0.91, -0.81)	<0.01	2021-2023	0.04 (-0.52, 0.29)	0.8574	–	–	–
India	LMIC	80.5	73.2	-0.69 (-0.78, -0.63)	<0.01	2009-2021	-0.79 (-1.1, -0.7)	<0.01	2021-2023	-0.05 (-0.75, 0.36)	0.5303	–	–	–
Pakistan	LMIC	17.6	27.2	3.13 (3, 3.31)	<0.01	2009-2011	1.54 (0.59, 3.21)	<0.01	2011-2017	4.84 (4.44, 5.58)	<0.01	2017-2023	1.99 (1.56, 2.39)	<0.01
Sri Lanka	LMIC	45.8	40.9	-0.81 (-0.83, -0.79)	<0.01	2009-2015	-0.74 (-0.8, -0.68)	<0.01	2015-2021	-1.11 (-1.17, -1.06)	<0.01	2021-2023	-0.13 (-0.33, 0.01)	0.06
**Sub-Saharan Africa**														
Angola	LMIC	7.8	13.5	3.92 (3.78, 4.12)	<0.01	2009-2011	2.65 (1.52, 4.48)	<0.01	2011-2016	5.45 (4.89, 6.2)	<0.01	2016-2023	3.2 (2.78, 3.52)	<0.01
Cameroon	LMIC	14.7	14.2	-0.32 (-0.6, -0.05)	0.02	2009-2012	1.16 (-0.12, 3.55)	0.07	2012-2018	-0.04 (-1.82, 0.75)	0.7027	2018-2023	-1.52 (-3.73, -0.38)	0.03
Côte d'Ivoire	LMIC	16.5	14.2	-1.07 (-1.16, -1.01)	<0.01	2009-2016	-0.77 (-0.97, -0.56)	<0.01	2016-2021	-1.61 (-1.9, -0.71)	<0.01	2021-2023	-0.75 (-1.47, -0.28)	<0.01
Ethiopia	LMIC	1.1	0.9	-1.8 (-1.98, -1.62)	<0.01	2009-2023	-1.8 (-1.98, -1.62)	<0.01	–	–	–	–	–	–
Ghana	LMIC	8.4	6.7	-1.76 (-2.07, -1.43)	<0.01	2009-2018	-1.13 (-1.52, 0)	0.05	2018-2023	-2.89 (-5.25, -1.98)	<0.01	–	–	–
Kenya	LMIC	20.2	20.0	-0.11 (-0.22, -0.02)	0.03	2009-2013	2.56 (2.1, 3.05)	<0.01	2013-2017	-0.82 (-1.21, -0.07)	0.0392	2017-2023	-1.39 (-2.27, -1.19)	<0.01
Nigeria	LMIC	55.6	26.5	-5.38 (-5.76, -5.16)	<0.01	2009-2021	-5.98 (-6.59, -5.72)	<0.01	2021-2023	-1.72 (-5.12, 0.05)	0.0548	–	–	–
South Africa	UMIC	9.6	15.7	3.61 (3.46, 3.77)	<0.01	2009-2014	6.43 (6.02, 7.51)	<0.01	2014-2018	4.92 (3.25, 5.54)	<0.01	2018-2023	-0.15 (-0.81, 0.27)	0.38
Tanzania	LMIC	2.5	4.7	4.72 (4.32, 4.98)	<0.01	2009-2017	5.54 (3.86, 7.71)	<0.01	2017-2021	2.52 (1.6, 6.55)	<0.01	2021-2023	5.9 (2.86, 7.97)	<0.01
Uganda	LMIC	7.4	8.6	0.97 (0.48, 1.39)	<0.01	2009-2016	-0.2 (-2.43, 1.13)	0.42	2016-2020	3.66 (-0.19, 4.77)	0.0552	2020-2023	0.2 (-4.13, 2.51)	0.97

Legend: Annual Average Percentage Change (AAPC), Annual Percentage Change (APC) and 95% Confidence Interval (CI) estimated using joinpoint regression models are shown. Original units and the description for the used indicator are reported in Supplementary Table 3. The results represent the percentage change in the density of non-chain outlets for each identified segment and for the entire analysis period (2009 to 2023), by country. Crude metrics for the first (2009) and last (2023) time points are reported. Two-tailed t-test was used to test whether AAPC and APC was statistically different from zero, with no adjustment made for multiple comparisons. LMICs=Low-and-middle-income countries; UMICs=Upper-and-middle-income countries; HICs=High-income countries.

Supplementary Table 7. Ratio of non-chain to chain outlets from 2009 to 2023, by country: joinpoint regression analysis.

	Income status	Ratio of non-chain to chain outlets[a]		AAPC		Segment 1			Segment 2			Segment 3		
		2009	2023	AAPC (95% CI)	p-value	Years	APC (95% CI)	p-value	Years	APC (95% CI)	p-value	Years	APC (95% CI)	p-value
**East Asia and Pacific**														
Australia	HIC	1.0	0.8	-1.2 (-1.42, -0.91)	<0.01	2009-2013	-1.73 (-2.55, 0.48)	0.09	2013-2018	-3.71 (-4.75, -2.95)	<0.01	2018-2023	1.82 (0.97, 3.07)	<0.01
Cambodia	LMIC	800.3	152.8	-11.12 (-11.66, -10.62)	<0.01	2009-2011	-10.32 (-15.2, -6.49)	<0.01	2011-2017	-16.2 (-18.31, -8.54)	<0.01	2017-2023	-6.02 (-7.5, -3.81)	<0.01
China	UMIC	84.7	33.0	-6.51 (-6.63, -6.32)	<0.01	2009-2011	-10.81 (-11.77, -9.13)	<0.01	2011-2015	-7.37 (-7.73, -5.31)	<0.01	2015-2023	-4.96 (-5.21, -4.48)	<0.01
Indonesia	LMIC	396.7	87.9	-10.5 (-10.97, -9.99)	<0.01	2009-2014	-15.01 (-18.33, -13.19)	<0.01	2014-2023	-7.9 (-8.74, -6.76)	<0.01	–	–	–
Japan	HIC	1.8	1.2	-3.1 (-3.21, -2.94)	<0.01	2009-2011	-2.52 (-3.57, -1.25)	<0.01	2011-2015	-5.46 (-5.81, -4.95)	<0.01	2015-2023	-2.04 (-2.24, -1.75)	<0.01
Laos	LMIC	2413.4	331.6	-13.23 (-14.52, -12.08)	<0.01	2009-2011	-9.43 (-22.73, 0.49)	0.06	2011-2015	-28.32 (-31.03, -7.94)	<0.01	2015-2023	-5.55 (-7.73, -2.41)	0.02
Malaysia	UMIC	8.5	2.6	-8.11 (-8.35, -7.88)	<0.01	2009-2023	-8.11 (-8.35, -7.88)	<0.01	–	–	–	–	–	–
Myanmar	LMIC	2727.7	249.2	-16.04 (-17, -14.96)	<0.01	2009-2011	-12.84 (-22.12, -3.9)	<0.01	2011-2017	-24.63 (-28.63, -16.54)	<0.01	2017-2023	-7.62 (-10.65, -2.98)	0.02
New Zealand	HIC	1.2	1.2	0.18 (-0.26, 0.56)	0.36	2009-2011	3.7 (0.26, 6.94)	0.02	2011-2023	-0.39 (-1.41, -0.12)	0.014	–	–	–
Philippines	LMIC	419.2	154.0	-6.8 (-7.23, -6.32)	<0.01	2009-2011	-6.71 (-10.07, -3.2)	<0.01	2011-2019	-10.23 (-11.92, -9.32)	<0.01	2019-2023	0.4 (-1.46, 3.94)	0.52
Singapore	HIC	1.2	1.2	-0.79 (-1.14, -0.36)	<0.01	2009-2011	-4.21 (-6.73, -0.19)	0.04	2011-2017	3.25 (2.26, 5.11)	<0.01	2017-2023	-3.55 (-4.66, -2.57)	<0.01
South Korea	HIC	4.3	0.9	-10.22 (-10.91, -9.57)	<0.01	2009-2018	-12.45 (-14.05, -11.42)	<0.01	2018-2023	-6.07 (-8.5, -1.8)	0.0156	–	–	–
Thailand	UMIC	52.0	26.7	-4.63 (-4.81, -4.46)	<0.01	2009-2012	-3.37 (-4.34, -1.67)	<0.01	2012-2021	-5.91 (-6.22, -5.7)	<0.01	2021-2023	-0.63 (-2.41, 0.41)	0.23
Vietnam	LMIC	838.5	86.0	-15.52 (-16.5, -14.49)	<0.01	2009-2013	-2.54 (-8.38, 3.21)	0.26	2013-2019	-29.15 (-32.23, -26.81)	<0.01	2019-2023	-4.65 (-9.67, 3.71)	0.10
**Europe and Central Asia**														
Austria	HIC	0.3	0.3	-0.11 (-0.32, 0.09)	0.22	2009-2011	2.43 (0.9, 3.88)	<0.01	2011-2019	-0.05 (-0.4, 0.22)	0.5859	2019-2023	-1.46 (-3.04, -0.86)	<0.01
Azerbaijan	UMIC	140.6	14.7	-15.38 (-15.95, -14.8)	<0.01	2009-2014	-7.62 (-9.42, -5.66)	<0.01	2014-2019	-28.26 (-29.68, -26.79)	<0.01	2019-2023	-6.79 (-9.25, -4.31)	<0.01
Belarus	UMIC	1.1	0.7	-3.2 (-3.6, -2.71)	<0.01	2009-2011	10.84 (6.51, 15.46)	<0.01	2011-2016	2.67 (-1.58, 3.58)	0.0836	2016-2023	-10.71 (-11.48, -10.04)	<0.01
Belgium	HIC	1.9	1.4	-1.92 (-2.11, -1.74)	<0.01	2009-2012	-4.38 (-6.42, -3.29)	<0.01	2012-2016	-0.23 (-0.97, 0.36)	0.2775	2016-2023	-1.81 (-2.47, -1.57)	<0.01
Bosnia and Herzegovina	UMIC	5.8	3.0	-4.46 (-4.76, -4.01)	<0.01	2009-2012	-9.83 (-13.84, -7.01)	<0.01	2012-2023	-2.94 (-3.37, -2.33)	<0.01	–	–	–
Bulgaria	UMIC	13.4	8.9	-3.51 (-4.48, -2.71)	<0.01	2009-2013	-12.54 (-17.45, -9.03)	<0.01	2013-2018	3.64 (0.75, 7.4)	0.0164	2018-2023	-2.82 (-9.57, -0.53)	0.02
Croatia	HIC	3.3	1.6	-5.14 (-5.36, -4.87)	<0.01	2009-2012	-7.28 (-9.42, -5.71)	<0.01	2012-2023	-4.54 (-4.79, -4.11)	<0.01	–	–	–
Czech Republic	HIC	1.6	1.1	-2.8 (-2.95, -2.67)	<0.01	2009-2018	-4.23 (-4.53, -4)	<0.01	2018-2023	-0.17 (-0.72, 0.81)	0.3975	–	–	–
Denmark	HIC	0.5	0.5	-0.69 (-1.09, -0.3)	<0.01	2009-2023	-0.69 (-1.09, -0.3)	<0.01	–	–	–	–	–	–
Estonia	HIC	1.3	0.7	-4.86 (-5.26, -4.54)	<0.01	2009-2019	-5.53 (-6.99, -5.09)	<0.01	2019-2023	-3.17 (-4.73, -0.02)	0.0496	–	–	–

Finland	HIC	0.4	0.5	1.34 (1.22, 1.45)	<0.01	2009-2014	-0.29 (-0.85, 0.07)	0.12	2014-2018	3.62 (3.02, 4)	<0.01	2018-2023	1.17 (0.68, 1.47)	<0.01
France	HIC	1.1	1.1	-0.07 (-0.21, 0.05)	0.21	2009-2014	-0.42 (-1.35, -0.03)	0.03	2014-2018	1.75 (1.01, 2.15)	<0.01	2018-2023	-1.15 (-1.69, -0.8)	<0.01
Georgia	UMIC	55.9	8.9	-12.37 (-13.12, -11.55)	<0.01	2009-2014	-6.96 (-9.67, -3.08)	<0.01	2014-2020	-18.44 (-21.6, -16.69)	<0.01	2020-2023	-8.45 (-12.81, -1.07)	0.03
Germany	HIC	1.0	0.8	-1.17 (-1.34, -1.05)	<0.01	2009-2020	-1.45 (-1.85, -1.29)	<0.01	2020-2023	-0.15 (-1.08, 1.08)	0.7367	–	–	–
Greece	HIC	5.9	3.8	-2.98 (-3.2, -2.75)	<0.01	2009-2014	-5.46 (-6.75, -4.63)	<0.01	2014-2023	-1.58 (-1.96, -1.12)	<0.01	–	–	–
Hungary	HIC	1.9	1.6	-1.03 (-1.62, 0.21)	0.09	2009-2011	-10.59 (-14.32, -0.96)	0.02	2011-2023	0.66 (-0.19, 2.85)	0.0964	–	–	–
Ireland	HIC	0.7	0.4	-3.58 (-3.71, -3.43)	<0.01	2009-2012	-1.45 (-2.51, -0.63)	0.02	2012-2018	-5.72 (-6.21, -5.37)	<0.01	2018-2023	-2.23 (-2.67, -1.6)	<0.01
Italy	HIC	4.3	4.0	-0.58 (-0.83, -0.32)	<0.01	2009-2018	0.28 (-0.11, 0.95)	0.16	2018-2023	-2.11 (-4.08, -1.21)	<0.01	–	–	–
Kazakhstan	UMIC	27.1	12.1	-5.38 (-6.42, -4.33)	<0.01	2009-2016	-4.17 (-5.36, 4.17)	0.08	2016-2023	-6.58 (-14.22, -5.39)	<0.01	–	–	–
Latvia	HIC	1.8	1.0	-3.71 (-4.22, -3.16)	<0.01	2009-2014	-11.08 (-13.84, -9.21)	<0.01	2014-2023	0.64 (-0.28, 1.76)	0.1604	–	–	–
Lithuania	HIC	1.7	1.1	-2.6 (-2.9, -2.29)	<0.01	2009-2015	-4.71 (-6.28, -3.82)	<0.01	2015-2023	-0.99 (-1.61, -0.08)	0.04	–	–	–
Netherlands	HIC	1.5	1.4	-0.89 (-1.02, -0.78)	<0.01	2009-2015	-0.37 (-1.2, -0.1)	0.01	2015-2019	0.84 (0.23, 1.19)	<0.01	2019-2023	-3.36 (-3.88, -2.91)	<0.01
North Macedonia	UMIC	18.7	13.2	-2.46 (-2.77, -2.12)	<0.01	2009-2011	-10.41 (-12.4, -7.51)	<0.01	2011-2015	0.42 (-0.81, 1.46)	0.6247	2015-2023	-1.8 (-3.27, -1.42)	<0.01
Norway	HIC	0.2	0.2	1.09 (0.83, 1.48)	<0.01	2009-2014	2.28 (1.51, 4.22)	<0.01	2014-2018	-1.25 (-2.14, 0.03)	0.0572	2018-2023	1.81 (0.87, 4.62)	<0.01
Poland	HIC	4.8	0.04	-29.46 (-30.19, -28.82)	<0.01	2009-2016	-17.16 (-18.99, -14.34)	<0.01	2016-2021	-42.02 (-43.83, -18.55)	<0.01	2021-2023	-34.4 (-40.5, -29.67)	<0.01
Portugal	HIC	6.0	2.2	-7.19 (-7.5, -6.88)	<0.01	2009-2015	-9.8 (-11.12, -8.91)	<0.01	2015-2023	-5.18 (-5.81, -4.35)	<0.01	–	–	–
Romania	HIC	30.7	4.5	-13.31 (-14.11, -12.25)	<0.01	2009-2013	-21.97 (-31.56, -17.38)	<0.01	2013-2023	-9.58 (-10.9, -7.61)	<0.01	–	–	–
Russia	UMIC	10.8	1.2	-14.51 (-14.94, -14.06)	<0.01	2009-2023	-14.51 (-14.94, -14.06)	<0.01	–	–	–	–	–	–
Serbia	UMIC	12.7	6.4	-4.88 (-5.04, -4.73)	<0.01	2009-2014	-6.39 (-7.22, -5.83)	<0.01	2014-2023	-4.04 (-4.29, -3.74)	<0.01	–	–	–
Slovakia	HIC	0.8	0.5	-3.88 (-4.34, -3.2)	<0.01	2009-2011	-11.84 (-14.85, -6.46)	<0.01	2011-2017	-4.05 (-5.65, -1.41)	0.0168	2017-2023	-0.89 (-2.74, 3.18)	0.56
Slovenia	HIC	1.0	0.6	-3.65 (-3.8, -3.5)	<0.01	2009-2012	-5.94 (-7.24, -5.04)	<0.01	2012-2019	-3.89 (-4.25, -3.38)	<0.01	2019-2023	-1.47 (-2.09, -0.44)	0.01
Spain	HIC	1.3	0.8	-3.15 (-3.4, -2.87)	<0.01	2009-2015	-1.43 (-2.13, -0.37)	0.02	2015-2023	-4.41 (-5.02, -3.97)	<0.01	–	–	–
Sweden	HIC	0.5	0.4	-1.54 (-1.89, -1.19)	<0.01	2009-2011	2.13 (-0.29, 5.07)	0.09	2011-2018	-1.04 (-2.45, -0.59)	0.0144	2018-2023	-3.67 (-6.02, -2.85)	<0.01
Switzerland	HIC	0.6	0.5	-1.56 (-1.74, -1.29)	<0.01	2009-2012	-1.14 (-2.09, 1.38)	0.16	2012-2016	-4.07 (-4.67, -3.06)	<0.01	2016-2023	-0.28 (-0.71, 0.5)	0.27
Turkey	UMIC	11.1	5.3	-5.37 (-5.59, -5.18)	<0.01	2009-2015	-6.16 (-6.92, -5.7)	<0.01	2015-2019	-2.45 (-3.58, -1.79)	<0.01	2019-2023	-7.03 (-8.54, -6.23)	<0.01
Ukraine	LMIC	22.6	8.8	-6.85 (-7.51, -6.13)	<0.01	2009-2014	-14.36 (-17.89, -11.9)	<0.01	2014-2023	-2.4 (-3.56, -0.91)	<0.01	–	–	–
United Kingdom	HIC	0.1	0.1	-4.24 (-4.4, -4.06)	<0.01	2009-2011	-9.14 (-10.23, -7.36)	<0.01	2011-2017	-5.46 (-5.87, -4.79)	<0.01	2017-2023	-1.28 (-1.71, -0.73)	<0.01
Uzbekistan	LMIC	585.2	97.1	-13.09 (-14.03, -12.14)	<0.01	2009-2018	-5.87 (-7.63, -3.88)	<0.01	2018-2023	-24.71 (-28.39, -21.43)	<0.01	–	–	–
**Latin America and Caribbean**														
Argentina	UMIC	53.2	41.5	-1.82 (-2.03, -1.61)	<0.01	2009-2017	-2.23 (-3.5, -1.88)	<0.01	2017-2023	-1.27 (-1.76, 0.35)	0.0876	–	–	–

Bolivia	LMIC	2048.9	1145.6	-4.08 (-4.48, -3.71)	<0.01	2009-2015	-8.5 (-10.96, -7.22)	<0.01	2015-2019	-2.45 (-8.39, -0.31)	0.0272	2019-2023	1.23 (-0.63, 4.69)	0.14
Brazil	UMIC	31.4	21.7	-2.44 (-2.7, -2.14)	<0.01	2009-2013	0.22 (-0.91, 2.18)	0.63	2013-2017	-7.84 (-8.66, -6.38)	<0.01	2017-2023	-0.46 (-1.08, 0.49)	0.24
Chile	HIC	128.3	72.4	-4.01 (-4.27, -3.7)	<0.01	2009-2013	-9.11 (-11.47, -7.73)	<0.01	2013-2023	-1.89 (-2.3, -1.4)	<0.01	–	–	–
Colombia	UMIC	165.0	134.4	-1.54 (-1.82, -1.29)	<0.01	2009-2018	-0.79 (-1.14, -0.22)	0.02	2018-2023	-2.86 (-4.5, -2.07)	<0.01	–	–	–
Costa Rica	UMIC	46.4	21.2	-5.77 (-6.44, -5.3)	<0.01	2009-2017	-5.85 (-9.01, -4.98)	<0.01	2017-2021	-1.8 (-4.1, -0.17)	0.0304	2021-2023	-12.93 (-17.3, -8.01)	<0.01
Dominican Republic	UMIC	178.1	127.4	-2.47 (-2.6, -2.34)	<0.01	2009-2013	-5.28 (-6.45, -4.68)	<0.01	2013-2023	-1.33 (-1.52, -1.11)	<0.01	–	–	–
Ecuador	UMIC	228.9	118.1	-4.64 (-5.02, -4.28)	<0.01	2009-2017	-6.75 (-7.83, -6.05)	<0.01	2017-2023	-1.76 (-2.93, 0.47)	0.0744	–	–	–
El Salvador	LMIC	38.5	21.4	-4.14 (-4.36, -3.92)	<0.01	2009-2014	-6.9 (-8.62, -6.18)	<0.01	2014-2018	-4.49 (-6.03, -2.02)	<0.01	2018-2023	-1.02 (-1.79, 0.67)	0.16
Guatemala	UMIC	333.3	150.7	-5.51 (-6.75, -4.35)	<0.01	2009-2012	1.43 (-3.72, 13.47)	0.57	2012-2017	-5.38 (-12.25, -2.06)	0.0128	2017-2023	-8.9 (-18.14, -2.83)	0.01
Honduras	LMIC	85.0	47.4	-4.09 (-4.15, -4.01)	<0.01	2009-2012	-9.51 (-9.83, -9.15)	<0.01	2012-2017	-3.54 (-3.85, -3.32)	<0.01	2017-2023	-1.72 (-1.91, -1.49)	<0.01
Mexico	UMIC	35.8	25.3	-2.5 (-3.2, -1.89)	<0.01	2009-2011	-8.35 (-13.23, -3.1)	<0.01	2011-2017	1.49 (0.07, 4.53)	0.0396	2017-2023	-4.39 (-7.74, -2.92)	<0.01
Panama	HIC	89.4	48.5	-4.28 (-4.51, -4.03)	<0.01	2009-2011	-2.3 (-4.78, -0.27)	0.02	2011-2016	-8.84 (-9.59, -8.28)	<0.01	2016-2023	-1.47 (-1.92, -1.01)	<0.01
Paraguay	UMIC	66.0	45.8	-2.49 (-2.79, -2.17)	<0.01	2009-2014	-1.56 (-2.34, 0.75)	0.09	2014-2023	-2.99 (-4.11, -2.67)	<0.01	–	–	–
Peru	UMIC	821.3	292.7	-6.67 (-7.53, -5.96)	<0.01	2009-2019	-9.74 (-11.22, -8.72)	<0.01	2019-2023	1.47 (-2.94, 12.02)	0.4011	–	–	–
Uruguay	HIC	23.9	24.6	0.19 (0.1, 0.3)	<0.01	2009-2012	6.11 (5.61, 6.71)	<0.01	2012-2023	-1.37 (-1.48, -1.25)	<0.01	–	–	–
**Middle East and North Africa**														
Algeria	LMIC	844.6	504.6	-3.68 (-4, -3.45)	<0.01	2009-2011	-3.23 (-5.15, -1.36)	<0.01	2011-2015	-5.37 (-5.86, -2.39)	<0.01	2015-2023	-2.94 (-4.71, -1.95)	<0.01
Egypt	LMIC	35.6	28.2	-1.75 (-2, -1.51)	<0.01	2009-2011	-3.94 (-5.68, -1.83)	<0.01	2011-2019	-0.72 (-1.02, 0.36)	0.1616	2019-2023	-2.7 (-4.61, -1.78)	<0.01
Iraq	UMIC	100.7	59.2	-3.73 (-3.92, -3.56)	<0.01	2009-2019	-4.65 (-4.96, -4.41)	<0.01	2019-2023	-1.37 (-2.31, 0.21)	0.0732	–	–	–
Israel	HIC	6.0	3.1	-4.46 (-4.78, -4.15)	<0.01	2009-2016	-8.68 (-9.73, -8.02)	<0.01	2016-2021	1.2 (-7.88, 2.34)	0.0956	2021-2023	-3.11 (-5.57, 0.14)	0.0636
Jordan	UMIC	48.0	20.1	-5.88 (-6.01, -5.73)	<0.01	2009-2013	-10.15 (-10.65, -9.59)	<0.01	2013-2023	-4.11 (-4.31, -3.91)	<0.01	–	–	–
Kuwait	HIC	3.3	1.3	-6.28 (-7.34, -5.46)	<0.01	2009-2011	-3.35 (-9.05, 3.1)	0.11	2011-2015	-9.08 (-10.6, -4.46)	<0.01	2015-2023	-5.57 (-11.53, -1.65)	0.01
Lebanon	LMIC	34.0	15.5	-5.49 (-5.74, -5.17)	<0.01	2009-2012	-4.01 (-5.33, -1.31)	0.02	2012-2016	-10.47 (-11.27, -9.16)	<0.01	2016-2023	-3.17 (-3.71, -2.39)	<0.01
Morocco	LMIC	192.0	153.5	-1.51 (-1.63, -1.38)	<0.01	2009-2013	0.97 (0.3, 1.49)	<0.01	2013-2019	-2.86 (-3.33, -2.59)	<0.01	2019-2023	-1.93 (-2.37, -0.87)	<0.01
Oman	HIC	1.7	0.9	-4.41 (-4.73, -4.09)	<0.01	2009-2014	-8.76 (-11.11, -7.74)	<0.01	2014-2018	-4.15 (-7.34, -1.66)	<0.01	2018-2023	-0.08 (-1.13, 2.51)	0.95
Qatar	HIC	4.3	2.0	-5.25 (-5.97, -4.53)	<0.01	2009-2023	-5.25 (-5.97, -4.53)	<0.01	–	–	–	–	–	–
Saudi Arabia	HIC	5.3	3.6	-2.62 (-2.94, -2)	<0.01	2009-2011	-6.29 (-8.44, -2.06)	<0.01	2011-2023	-1.99 (-5.13, 0.2)	0.0592	–	–	–
Tunisia	LMIC	419.9	174.4	-6.4 (-6.73, -6.04)	<0.01	2009-2015	-9.67 (-11.14, -8.63)	<0.01	2015-2023	-3.87 (-4.59, -2.92)	<0.01	–	–	–
United Arab Emirates	HIC	5.3	2.1	-6.73 (-7.75, -5.71)	<0.01	2009-2023	-6.73 (-7.75, -5.71)	<0.01	–	–	–	–	–	–
**North America**														

Canada	HIC	1.7	1.4	-1.03 (-1.17, -0.88)	<0.01	2009-2014	-2.12 (-3.16, -1.24)	<0.01	2014-2018	-1.08 (-2.1, 0.15)	0.07	2018-2023	0.11 (-0.8, 1.17)	0.48
USA	HIC	0.5	0.7	2.35 (1.97, 2.9)	<0.01	2009-2012	10.51 (7.19, 14.09)	<0.01	2012-2023	0.22 (-0.32, 0.68)	0.3351	–	–	–
**South Asia**														
Bangladesh	LMIC	1598.7	883.0	-4.31 (-4.5, -4.11)	<0.01	2009-2012	-8.94 (-10.25, -7.41)	<0.01	2012-2023	-3 (-3.23, -2.76)	<0.01	–	–	–
India	LMIC	821.5	487.1	-3.56 (-3.75, -3.36)	<0.01	2009-2012	-5.12 (-6.45, -4.13)	<0.01	2012-2019	-3.87 (-4.37, -2.95)	<0.01	2019-2023	-1.82 (-2.6, -0.16)	0.03
Pakistan	LMIC	479.7	472.9	0.02 (-0.26, 0.31)	0.88	2009-2017	3.17 (2.67, 3.95)	<0.01	2017-2021	-5.45 (-6.19, 2.57)	0.094	2021-2023	-1.14 (-4.18, 1.09)	0.19
Sri Lanka	LMIC	253.7	43.6	-11.65 (-12.01, -11.31)	<0.01	2009-2017	-17.47 (-18.08, -16.82)	<0.01	2017-2023	-3.26 (-4.3, -2.26)	<0.01	–	–	–
**Sub-Saharan Africa**														
Angola	LMIC	13.9	26.2	4.71 (4.56, 4.87)	<0.01	2009-2012	0.52 (-0.22, 1.33)	0.14	2012-2018	6.63 (6.27, 7.16)	<0.01	2018-2023	5.01 (4.22, 5.44)	<0.01
Cameroon	LMIC	60.5	45.8	-1.86 (-2.01, -1.66)	<0.01	2009-2012	-1.54 (-2.34, 0.26)	0.08	2012-2017	-3.74 (-4.51, -3.19)	<0.01	2017-2023	-0.44 (-0.89, 0.23)	0.16
Côte d'Ivoire	LMIC	195.8	85.1	-5.84 (-6.13, -5.53)	<0.01	2009-2011	-1.15 (-4.41, 1.12)	0.29	2011-2018	-9.17 (-10.01, -8.65)	<0.01	2018-2023	-2.87 (-3.86, -1.57)	<0.01
Ethiopia	LMIC	183.8	116.6	-3.13 (-3.38, -2.83)	<0.01	2009-2013	-2.56 (-3.56, -0.32)	0.03	2013-2019	-5.19 (-6.43, -4.55)	<0.01	2019-2023	-0.54 (-1.75, 1.44)	0.40
Ghana	LMIC	66.9	27.5	-6.2 (-6.56, -5.88)	<0.01	2009-2019	-7.88 (-8.45, -7.43)	<0.01	2019-2023	-1.85 (-3.61, 0.96)	0.142	–	–	–
Kenya	LMIC	235.2	167.4	-2.46 (-2.83, -2.07)	<0.01	2009-2015	-5.06 (-7.39, -3.92)	<0.01	2015-2023	-0.46 (-1.25, 0.78)	0.2963	–	–	–
Nigeria	LMIC	1125.7	635.5	-4.04 (-4.48, -3.69)	<0.01	2009-2016	-10.13 (-10.93, -9.41)	<0.01	2016-2020	5.08 (3.21, 6.36)	0.0328	2020-2023	-0.91 (-4.87, 1.12)	0.20
South Africa	UMIC	8.2	12.3	2.94 (2.79, 3.13)	<0.01	2009-2011	1.83 (0.93, 3.49)	<0.01	2011-2019	4.26 (4.01, 4.82)	<0.01	2019-2023	0.9 (0.18, 1.64)	0.03
Tanzania	LMIC	21.0	27.8	1.87 (1.64, 2.14)	<0.01	2009-2011	-1.47 (-3.15, 1.03)	0.19	2011-2015	3.64 (2.66, 4.49)	<0.01	2015-2023	1.84 (1.01, 2.12)	0.02
Uganda	LMIC	57.0	41.9	-2.32 (-2.68, -1.95)	<0.01	2009-2016	-6.55 (-7.71, -5.7)	<0.01	2016-2023	2.11 (1.21, 3.31)	<0.01	–	–	–

Legend: Annual Average Percentage Change (AAPC), Annual Percentage Change (APC) and 95% Confidence Interval (CI) estimated using joinpoint regression models are shown. Original units and the description for the used indicator are reported in Supplementary Table 3. The results represent the percentage change in the ratio of non-chain to chain outlets for each identified segment and for the entire analysis period (2009 to 2023), by country. Crude metrics for the first (2009) and last (2023) time points are reported. Two-tailed t-test was used to test whether AAPC and APC was statistically different from zero, with no adjustment made for multiple comparisons. LMICs=Low-and-middle-income countries; UMICs=Upper-and-middle-income countries; HICs=High-income countries.

Supplementary Table 8. Percentage of sales from chain retailers from 2009 to 2023, by country: joinpoint regression analysis.

	Income status	Percentage sales from chain retail[a]		AAPC		Segment 1			Segment 2			Segment 3		
		2009	2023	AAPC (95% CI)	p-value	Years	APC (95% CI)	p-value	Years	APC (95% CI)	p-value	Years	APC (95% CI)	p-value
**East Asia and Pacific**														
Australia	HIC	92.7	93.7	0.08 (0.05, 0.1)	<0.01	2009-2011	0.41 (0.2, 0.59)	<0.01	2011-2017	0.13 (-0.05, 0.17)	0.08	2017-2023	-0.08 (-0.21, -0.02)	0.0384
Cambodia	LMIC	4.3	17.6	10.43 (9.8, 11.11)	<0.01	2009-2011	22.43 (16.54, 28.28)	<0.01	2011-2018	12.64 (10.62, 13.66)	<0.01	2018-2023	3.07 (0.51, 4.95)	0.0256
China	UMIC	30.7	38.5	1.62 (1.39, 1.82)	<0.01	2009-2011	3.28 (1.63, 4.92)	<0.01	2011-2021	1.02 (0.24, 1.17)	<0.01	2021-2023	2.97 (1.34, 4.13)	<0.01
Indonesia	LMIC	11.2	22.8	5.04 (3.94, 6.11)	<0.01	2009-2023	5.04 (3.94, 6.11)	<0.01	–	–	–	–	–	–
Japan	HIC	85.5	91.3	0.48 (0.46, 0.5)	<0.01	2009-2014	0.67 (0.61, 0.77)	<0.01	2014-2020	0.43 (0.39, 0.53)	<0.01	2020-2023	0.26 (0.06, 0.35)	<0.01
Laos	LMIC	1.2	7.6	14.49 (13.49, 15.58)	<0.01	2009-2015	26.44 (22.97, 30.51)	<0.01	2015-2023	6.27 (4.15, 8.23)	<0.01	–	–	–
Malaysia	UMIC	51.7	66.7	1.75 (1.58, 1.95)	<0.01	2009-2011	7.93 (5.3, 9.38)	<0.01	2011-2023	0.76 (0.56, 0.94)	<0.01	–	–	–
Myanmar	LMIC	0.4	2.8	13.84 (12.87, 14.99)	<0.01	2009-2011	10.78 (4.44, 22.16)	<0.01	2011-2017	25.03 (17.56, 30.2)	<0.01	2017-2023	4.6 (1.31, 7.34)	0.0232
New Zealand	HIC	91.1	93.0	0.14 (0.13, 0.15)	<0.01	2009-2018	0.23 (0.2, 0.25)	<0.01	2018-2023	-0.01 (-0.08, 0.04)	0.57	–	–	–
Philippines	LMIC	18.1	31.0	3.91 (3.51, 4.32)	<0.01	2009-2016	7.43 (6.39, 8.65)	<0.01	2016-2023	0.5 (-0.56, 1.45)	0.29	–	–	–
Singapore	HIC	74.5	80.6	0.84 (0.5, 1.19)	<0.01	2009-2023	0.84 (0.5, 1.19)	<0.01	–	–	–	–	–	–
South Korea	HIC	84.0	93.2	0.81 (0.73, 0.91)	<0.01	2009-2013	0.07 (-0.84, 0.52)	0.78	2013-2023	1.11 (0.98, 1.29)	<0.01	–	–	–
Thailand	UMIC	51.6	71.7	2.34 (2.11, 2.55)	<0.01	2009-2013	3.06 (2.36, 4.97)	<0.01	2013-2023	2.05 (1.02, 2.24)	<0.01	–	–	–
Vietnam	LMIC	5.2	12.7	6.9 (6.32, 7.47)	<0.01	2009-2023	6.9 (6.32, 7.47)	<0.01	–	–	–	–	–	–
**Europe and Central Asia**														
Austria	HIC	95.4	96.0	0.05 (0.04, 0.06)	<0.01	2009-2013	0.14 (0.1, 0.19)	<0.01	2013-2019	-0.02 (-0.06, 0)	0.09	2019-2023	0.08 (0.04, 0.15)	<0.01
Azerbaijan	UMIC	14.7	47.4	8.88 (8.44, 9.36)	<0.01	2009-2013	3.86 (1.07, 5.81)	<0.01	2013-2018	18.72 (16.9, 20.65)	<0.01	2018-2023	3.71 (2.34, 5.05)	<0.01
Belarus	UMIC	50.1	77.7	3.45 (2.97, 3.95)	<0.01	2009-2019	4.19 (3.73, 5.89)	<0.01	2019-2023	1.6 (-2.39, 3.3)	0.26	–	–	–
Belgium	HIC	84.5	88.4	0.33 (0.32, 0.35)	<0.01	2009-2023	0.33 (0.32, 0.35)	<0.01	–	–	–	–	–	–
Bosnia and Herzegovina	UMIC	50.0	66.7	2.17 (1.94, 2.51)	<0.01	2009-2012	4.55 (3.07, 7.74)	<0.01	2012-2016	-0.2 (-0.97, 1.05)	0.94	2016-2023	2.53 (2.02, 4.11)	<0.01
Bulgaria	UMIC	45.1	67.1	2.92 (2.55, 3.37)	<0.01	2009-2012	6.63 (4.67, 10.03)	<0.01	2012-2019	1.24 (-0.18, 1.85)	0.08	2019-2023	3.14 (1.72, 5.84)	0.014
Croatia	HIC	75.7	83.0	0.66 (0.6, 0.7)	<0.01	2009-2011	0.93 (0.61, 1.26)	<0.01	2011-2023	0.61 (0.33, 0.68)	<0.01	–	–	–
Czech Republic	HIC	83.5	92.2	0.67 (0.62, 0.72)	<0.01	2009-2020	0.82 (0.77, 0.89)	<0.01	2020-2023	0.12 (-0.46, 0.42)	0.40	–	–	–
Denmark	HIC	94.8	94.9	0.01 (-0.02, 0.05)	0.40	2009-2023	0.01 (-0.02, 0.05)	0.40	–	–	–	–	–	–
Estonia	HIC	88.9	95.8	0.53 (0.51, 0.54)	<0.01	2009-2014	1 (0.94, 1.11)	<0.01	2014-2018	0.47 (0.29, 0.67)	<0.01	2018-2023	0.1 (-0.01, 0.16)	0.0716

Finland	HIC	98.3	98.7	0.03 (0.02, 0.04)	<0.01	2009-2023	0.03 (0.02, 0.04)	<0.01	–	–	–	–	–	–
France	HIC	92.3	90.2	-0.18 (-0.21, -0.14)	<0.01	2009-2014	0.01 (-0.1, 0.14)	0.80	2014-2019	-0.64 (-0.79, -0.53)	<0.01	2019-2023	0.18 (0.04, 0.4)	0.0132
Georgia	UMIC	16.1	55.9	9.69 (8.93, 10.25)	<0.01	2009-2016	8.35 (5.46, 9.6)	<0.01	2016-2021	14.7 (12.67, 17.42)	<0.01	2021-2023	2.39 (-3.07, 7.82)	0.26
Germany	HIC	91.1	94.1	0.23 (0.22, 0.25)	<0.01	2009-2013	0.06 (-0.06, 0.12)	0.18	2013-2018	0.24 (0.15, 0.31)	<0.01	2018-2023	0.37 (0.33, 0.47)	<0.01
Greece	HIC	54.8	74.6	2.24 (1.97, 2.52)	<0.01	2009-2012	4.38 (3.32, 6.87)	<0.01	2012-2020	2.01 (1.52, 2.49)	<0.01	2020-2023	0.73 (-1.38, 1.83)	0.30
Hungary	HIC	80.4	82.2	0.17 (0.08, 0.27)	<0.01	2009-2015	0.96 (0.68, 1.32)	<0.01	2015-2023	-0.42 (-0.64, -0.24)	<0.01	–	–	–
Ireland	HIC	97.5	98.6	0.08 (0.08, 0.09)	<0.01	2009-2020	0.1 (0.1, 0.11)	<0.01	2020-2023	0 (-0.07, 0.04)	0.92	–	–	–
Italy	HIC	83.1	83.8	0.05 (0.01, 0.1)	<0.01	2009-2018	0.12 (0.08, 0.3)	<0.01	2018-2023	-0.06 (-0.4, 0.04)	0.23	–	–	–
Kazakhstan	UMIC	15.5	41.8	7.52 (6.97, 8.07)	<0.01	2009-2015	3.94 (2.24, 5.34)	<0.01	2015-2020	16.31 (14.15, 18.99)	<0.01	2020-2023	0.92 (-4.5, 4.2)	0.52
Latvia	HIC	85.3	95.1	0.82 (0.76, 0.87)	<0.01	2009-2017	1.17 (1.08, 1.29)	<0.01	2017-2023	0.34 (0.16, 0.49)	<0.01	–	–	–
Lithuania	HIC	87.1	90.2	0.3 (0.25, 0.34)	<0.01	2009-2016	0.52 (0.42, 0.66)	<0.01	2016-2023	0.07 (-0.08, 0.17)	0.25	–	–	–
Netherlands	HIC	90.9	93.4	0.19 (0.17, 0.2)	<0.01	2009-2016	0.15 (0.07, 0.19)	<0.01	2016-2020	0.29 (0.17, 0.34)	<0.01	2020-2023	0.15 (-0.01, 0.24)	0.061
North Macedonia	UMIC	29.8	36.2	1.3 (1.14, 1.56)	<0.01	2009-2012	3.69 (2.51, 6.36)	<0.01	2012-2023	0.66 (0.41, 0.86)	<0.01	–	–	–
Norway	HIC	98.6	99.0	0.02 (0.01, 0.03)	<0.01	2009-2013	-0.02 (-0.12, 0.02)	0.30	2013-2018	0.11 (0.07, 0.15)	<0.01	2018-2023	-0.02 (-0.08, 0.01)	0.10
Poland	HIC	62.2	95.6	3.17 (2.96, 3.4)	<0.01	2009-2014	4.19 (3.51, 5.64)	<0.01	2014-2023	2.6 (2.07, 2.89)	<0.01	–	–	–
Portugal	HIC	86.5	92.4	0.46 (0.42, 0.53)	<0.01	2009-2014	0.68 (0.55, 0.97)	<0.01	2014-2018	-0.07 (-0.22, 0.19)	0.68	2018-2023	0.67 (0.51, 1.13)	<0.01
Romania	HIC	42.2	67.6	3.46 (3.24, 3.71)	<0.01	2009-2013	6.78 (5.55, 8.4)	<0.01	2013-2023	2.16 (1.82, 2.47)	<0.01	–	–	–
Russia	UMIC	52.0	86.3	3.64 (3.48, 3.77)	<0.01	2009-2012	2.87 (1.52, 3.62)	<0.01	2012-2016	4.95 (4.3, 5.37)	<0.01	2016-2023	3.22 (2.75, 3.46)	<0.01
Serbia	UMIC	40.7	51.3	1.42 (1.19, 1.68)	<0.01	2009-2014	4.32 (3.43, 5.33)	<0.01	2014-2019	-1.75 (-2.76, -0.89)	<0.01	2019-2023	1.88 (0.9, 3.88)	<0.01
Slovakia	HIC	88.3	94.1	0.3 (0.19, 0.42)	<0.01	2009-2023	0.3 (0.19, 0.42)	<0.01	–	–	–	–	–	–
Slovenia	HIC	84.9	88.1	0.24 (0.18, 0.28)	<0.01	2009-2016	-0.12 (-0.31, -0.01)	0.04	2016-2020	0.85 (0.08, 1)	0.04	2020-2023	0.27 (-0.28, 0.59)	0.19
Spain	HIC	93.3	96.3	0.21 (0.19, 0.22)	<0.01	2009-2023	0.21 (0.19, 0.22)	<0.01	–	–	–	–	–	–
Sweden	HIC	95.7	96.7	0.07 (0.06, 0.09)	<0.01	2009-2012	-0.02 (-0.14, 0.05)	0.42	2012-2017	0.18 (0.15, 0.23)	<0.01	2017-2023	0.03 (0, 0.06)	0.06
Switzerland	HIC	94.9	95.1	0.02 (0, 0.03)	<0.01	2009-2014	-0.03 (-0.13, 0.01)	0.15	2014-2020	0.1 (0.07, 0.16)	<0.01	2020-2023	-0.06 (-0.19, 0)	0.07
Turkey	UMIC	28.1	58.3	5.62 (5.35, 5.9)	<0.01	2009-2014	8.56 (7.5, 9.97)	<0.01	2014-2023	4.02 (3.5, 4.45)	<0.01	–	–	–
Ukraine	LMIC	49.9	86.1	3.95 (3.63, 4.2)	<0.01	2009-2019	2.71 (2.19, 3.1)	<0.01	2019-2023	7.1 (5.54, 10.59)	<0.01	–	–	–
United Kingdom	HIC	92.9	94.7	0.13 (0.11, 0.15)	<0.01	2009-2011	0.34 (0.14, 0.5)	<0.01	2011-2023	0.1 (0.05, 0.11)	0.03	–	–	–
Uzbekistan	LMIC	3.7	11.0	8.4 (7.76, 9.51)	<0.01	2009-2011	0.1 (-4.08, 8.54)	0.74	2011-2023	9.84 (9.05, 12.21)	<0.01	–	–	–
**Latin America and Caribbean**														
Argentina	UMIC	46.2	42.3	-0.77 (-1.16, -0.36)	<0.01	2009-2016	-0.09 (-0.7, 2.75)	0.94	2016-2023	-1.44 (-4.1, -0.83)	<0.01	–	–	–

Bolivia	LMIC	9.7	10.8	-0.33 (-1.48, 0.68)	0.5	2009-2018	3.59 (2.05, 5.78)	<0.01	2018-2023	-7.02 (-12.41, -3.75)	<0.01	–	–	–
Brazil	UMIC	55.8	66.6	1.32 (0.98, 1.61)	<0.01	2009-2013	0.41 (-2.48, 1.62)	0.81	2013-2017	4.45 (2.97, 5.47)	<0.01	2017-2023	-0.11 (-1.26, 0.57)	0.60
Chile	HIC	66.7	70.5	0.43 (0.18, 0.67)	<0.01	2009-2023	0.43 (0.18, 0.67)	<0.01	–	–	–	–	–	–
Colombia	UMIC	33.6	32.7	-0.11 (-0.65, 0.34)	0.42	2009-2014	-1.79 (-4.57, 1.74)	0.10	2014-2021	-0.01 (-2.16, 1.14)	0.80	2021-2023	3.86 (-0.12, 6.35)	0.06
Costa Rica	UMIC	68.7	85.3	1.52 (1.29, 1.69)	<0.01	2009-2019	1.16 (0.22, 1.43)	0.03	2019-2023	2.42 (1.53, 4.11)	<0.01	–	–	–
Dominican Republic	UMIC	38.5	56.6	2.81 (2.7, 2.97)	<0.01	2009-2011	8.06 (6.94, 9.35)	<0.01	2011-2017	1.41 (0.9, 1.72)	<0.01	2017-2023	2.52 (2.21, 3.36)	<0.01
Ecuador	UMIC	40.3	59.7	2.91 (2.73, 3.1)	<0.01	2009-2015	4.44 (3.91, 5.5)	<0.01	2015-2020	2.52 (1.82, 4.09)	<0.01	2020-2023	0.53 (-0.97, 1.59)	0.40
El Salvador	LMIC	41.1	55.5	2.12 (1.86, 2.41)	<0.01	2009-2011	-0.3 (-1.64, 2.26)	0.81	2011-2019	3.24 (2.92, 4.43)	<0.01	2019-2023	1.13 (-0.71, 1.97)	0.10
Guatemala	UMIC	15.1	26.2	3.81 (3.38, 4.24)	<0.01	2009-2016	5.3 (4.38, 7.38)	<0.01	2016-2023	2.34 (0.15, 3.21)	0.04	–	–	–
Honduras	LMIC	22.1	33.7	3.05 (2.8, 3.41)	<0.01	2009-2012	7.15 (5.25, 10.45)	<0.01	2012-2016	0.66 (-0.16, 1.96)	0.10	2016-2023	2.71 (2.21, 4.59)	<0.01
Mexico	UMIC	67.9	70.9	0.47 (-0.35, 1.12)	0.22	2009-2019	-1.55 (-3.4, -0.63)	<0.01	2019-2023	5.71 (1.96, 13.58)	<0.01	–	–	–
Panama	HIC	47.2	50.8	0.57 (0.35, 0.8)	<0.01	2009-2016	-0.54 (-1.56, -0.01)	0.05	2016-2023	1.7 (1.16, 2.75)	<0.01	–	–	–
Paraguay	UMIC	17.1	23.6	2.31 (2.02, 2.67)	<0.01	2009-2013	1.2 (-1.25, 2.47)	0.22	2013-2023	2.76 (2.31, 4.38)	<0.01	–	–	–
Peru	UMIC	20.6	28.3	2.34 (0.52, 3.61)	<0.01	2009-2011	8.96 (0.73, 20.45)	0.01	2011-2023	1.28 (-8.66, 3.77)	0.20	–	–	–
Uruguay	HIC	48.0	52.6	0.78 (0.5, 1.08)	<0.01	2009-2014	-1.64 (-3.61, -0.58)	<0.01	2014-2023	2.15 (1.67, 2.83)	<0.01	–	–	–
**Middle East and North Africa**														
Algeria	LMIC	7.8	11.9	2.71 (1.72, 3.46)	<0.01	2009-2011	10.9 (2.82, 17.51)	<0.01	2011-2023	1.41 (-0.72, 2.05)	0.08	–	–	–
Egypt	LMIC	16.0	23.9	2.74 (1.96, 3.36)	<0.01	2009-2015	0.16 (-3.12, 1.71)	0.97	2015-2019	8.94 (5.57, 11.1)	<0.01	2019-2023	0.67 (-5.34, 3.28)	0.87
Iraq	UMIC	4.4	8.8	4.12 (2.66, 5.49)	<0.01	2009-2018	8.07 (6.14, 11.36)	<0.01	2018-2023	-2.62 (-10.65, 1.58)	0.20	–	–	–
Israel	HIC	57.9	64.8	0.78 (0.48, 1.09)	<0.01	2009-2016	2.55 (1.86, 3.51)	<0.01	2016-2023	-0.96 (-1.84, -0.29)	<0.01	–	–	–
Jordan	UMIC	32.9	48.8	2.62 (2.16, 3.06)	<0.01	2009-2018	3.81 (3.2, 4.91)	<0.01	2018-2023	0.51 (-2.4, 1.85)	0.56	–	–	–
Kuwait	HIC	86.8	92.6	0.51 (0.44, 0.58)	<0.01	2009-2023	0.51 (0.44, 0.58)	<0.01	–	–	–	–	–	–
Lebanon	LMIC	22.9	46.7	5.47 (4.72, 6.28)	<0.01	2009-2014	0.3 (-4.91, 3.05)	0.85	2014-2023	8.45 (7.15, 10.43)	<0.01	–	–	–
Morocco	LMIC	12.0	22.1	4.46 (4.23, 4.74)	<0.01	2009-2012	11.42 (8.91, 13.08)	<0.01	2012-2023	2.64 (2.32, 2.91)	<0.01	–	–	–
Oman	HIC	50.0	67.7	2.21 (2.06, 2.37)	<0.01	2009-2015	1.19 (0.66, 1.56)	<0.01	2015-2021	3.65 (3.29, 4.34)	<0.01	2021-2023	1.05 (-0.06, 2.47)	0.06
Qatar	HIC	87.1	91.3	0.32 (0.27, 0.35)	<0.01	2009-2015	-0.01 (-0.23, 0.09)	0.78	2015-2019	0.76 (0.42, 0.89)	<0.01	2019-2023	0.37 (-0.02, 0.54)	0.06
Saudi Arabia	HIC	55.4	64.0	1.08 (0.82, 1.34)	<0.01	2009-2015	0.35 (-1.45, 0.93)	0.49	2015-2023	1.64 (1.25, 2.92)	<0.01	–	–	–
Tunisia	LMIC	10.5	15.0	2.42 (1.76, 3.35)	<0.01	2009-2011	-1.04 (-5.66, 6.18)	0.78	2011-2017	6.62 (0.05, 10.34)	0.05	2017-2023	-0.48 (-3.5, 1.38)	0.45
United Arab Emirates	HIC	91.1	94.5	0.26 (0.23, 0.3)	<0.01	2009-2013	0.17 (-0.07, 0.29)	0.12	2013-2020	0.43 (0.38, 0.57)	<0.01	2020-2023	0 (-0.3, 0.2)	0.99
**North America**														

Canada	HIC	78.3	77.3	-0.08 (-0.19, 0)	0.06	2009-2020	-0.24 (-0.55, -0.15)	<0.01	2020-2023	0.52 (-0.04, 1.27)	0.08	–	–	–
USA	HIC	96.1	96.7	0.04 (0.03, 0.05)	<0.01	2009-2014	-0.01 (-0.07, 0.02)	0.56	2014-2018	0.17 (0.11, 0.2)	<0.01	2018-2023	-0.02 (-0.09, 0.01)	0.1716
**South Asia**														
Bangladesh	LMIC	1.5	2.8	4.33 (3.66, 5.17)	<0.01	2009-2013	11.86 (8.4, 18.04)	<0.01	2013-2023	1.46 (0.36, 2.34)	0.01	–	–	–
India	LMIC	3.1	5.1	3.21 (2.69, 3.74)	<0.01	2009-2017	6.65 (5.61, 7.95)	<0.01	2017-2023	-1.19 (-2.95, 0.29)	0.11	–	–	–
Pakistan	LMIC	3.0	7.0	6.55 (6, 6.98)	<0.01	2009-2014	4.82 (0.93, 6.29)	0.02	2014-2018	11.11 (8.68, 12.61)	<0.01	2018-2023	4.74 (2.08, 5.93)	0.01
Sri Lanka	LMIC	13.5	38.4	7.71 (7.24, 8.24)	<0.01	2009-2012	20.38 (17.53, 25.53)	<0.01	2012-2016	9.84 (6.07, 12.92)	<0.01	2016-2023	1.55 (0.3, 2.32)	0.03
**Sub-Saharan Africa**														
Angola	LMIC	28.7	15.3	-4.89 (-5.72, -4.05)	<0.01	2009-2016	-6.16 (-11.65, -0.33)	0.05	2016-2023	-3.59 (-9.24, 2.29)	0.14	–	–	–
Cameroon	LMIC	18.4	25.0	2.43 (2.14, 2.7)	<0.01	2009-2017	1.57 (0.39, 2.06)	0.02	2017-2023	3.58 (2.81, 5.38)	<0.01	–	–	–
Côte d'Ivoire	LMIC	21.4	28.1	1.96 (1.61, 2.31)	<0.01	2009-2023	1.96 (1.61, 2.31)	<0.01	–	–	–	–	–	–
Ethiopia	LMIC	55.0	38.9	-2.53 (-2.73, -2.37)	<0.01	2009-2013	-4.63 (-6.04, -3.9)	<0.01	2013-2017	-0.31 (-1.29, 0.28)	0.24	2017-2023	-2.59 (-3.37, -2.23)	<0.01
Ghana	LMIC	9.4	17.2	4.41 (3.6, 5.25)	<0.01	2009-2015	14.9 (12, 17.87)	<0.01	2015-2023	-2.83 (-4.44, -1.19)	<0.01	–	–	–
Kenya	LMIC	23.5	28.4	1.16 (0.75, 1.52)	<0.01	2009-2014	6.96 (5.42, 8.24)	<0.01	2014-2021	-3.01 (-4.37, -2.32)	<0.01	2021-2023	1.95 (-1.59, 4.46)	0.42
Nigeria	LMIC	2.8	6.3	5.99 (5.64, 6.34)	<0.01	2009-2016	12.5 (11.64, 13.35)	<0.01	2016-2023	-0.15 (-0.91, 0.59)	0.66	–	–	–
South Africa	UMIC	80.1	74.3	-0.44 (-0.66, -0.22)	<0.01	2009-2017	-1.41 (-2.05, -1.03)	<0.01	2017-2023	0.86 (0.27, 1.84)	<0.01	–	–	–
Tanzania	LMIC	12.3	10.3	-1.04 (-1.76, -0.41)	<0.01	2009-2018	-2.5 (-5.67, -1.51)	<0.01	2018-2023	1.64 (-0.69, 7.47)	0.18	–	–	–
Uganda	LMIC	9.7	15.4	3.89 (2.49, 5.33)	<0.01	2009-2016	11.33 (8.13, 16.14)	<0.01	2016-2023	-3.05 (-6.88, -0.25)	0.03	–	–	–

Legend: Annual Average Percentage Change (AAPC), Annual Percentage Change (APC) and 95% Confidence Interval (CI) estimated using joinpoint regression models are shown. Original units and the description for the used indicator are reported in Supplementary Table 3. The results represent the percentage change in the percentage of grocery sales from chain retailers for each identified segment and for the entire analysis period (2009 to 2023), by country. Crude metrics for the first (2009) and last (2023) time points are reported. Two-tailed t-test was used to test whether AAPC and APC was statistically different from zero, with no adjustment made for multiple comparisons. LMICs=Low-and-middle-income countries; UMICs=Upper-and-middle-income countries; HICs=High-income countries.

Supplementary Table 9. Unhealthy food sales (kg per capita) from 2009 to 2023, by country: joinpoint regression analysis.

	Income status	Kg per capita		AAPC		Segment 1			Segment 2			Segment 3		
		2009	2023	AAPC (95% CI)	p-value	Years	APC (95% CI)	p-value	Years	APC (95% CI)	p-value	Years	APC (95% CI)	p-value
**East Asia and Pacific**														
Australia	HIC	116.0	121.1	0.23 (0, 0.46)	0.04	2009-2023	0.23 (0, 0.46)	0.047	–	–	–	–	–	–
Cambodia*	LMIC	3.6	6.0	3.51 (3.13, 3.93)	<0.01	2009-2021	4.18 (3.89, 4.86)	<0.01	2021-2023	-0.44 (-3.22, 3.18)	0.963	–	–	–
China	UMIC	17.7	23.0	2.03 (1.72, 2.41)	<0.01	2009-2013	4.17 (2.74, 7.01)	<0.01	2013-2023	1.19 (0.58, 1.57)	0.01	–	–	–
Indonesia	LMIC	11.3	15.9	2.51 (2.36, 2.67)	<0.01	2009-2016	3.18 (2.85, 3.71)	<0.01	2016-2023	1.85 (1.34, 2.16)	<0.01	–	–	–
Japan	HIC	99.2	101.3	0.28 (0.13, 0.43)	<0.01	2009-2023	0.28 (0.13, 0.43)	<0.01	–	–	–	–	–	–
Laos*	LMIC	4.2	6.3	2.87 (2.63, 3.1)	<0.01	2009-2016	4.57 (4.13, 5.36)	<0.01	2016-2021	2.21 (1.65, 3.93)	<0.01	2021-2023	-1.27 (-3.18, 0.78)	0.19
Malaysia	UMIC	21.9	25.7	1.11 (0.94, 1.25)	<0.01	2009-2021	1.66 (1.53, 1.83)	<0.01	2021-2023	-2.16 (-3.37, -0.44)	0.02	–	–	–
Myanmar*	LMIC	5.3	8.5	3.29 (3.06, 3.47)	<0.01	2009-2015	3.55 (2.38, 4.03)	<0.01	2015-2021	5.3 (4.81, 6.13)	<0.01	2021-2023	-3.26 (-4.9, -0.92)	<0.01
New Zealand	HIC	106.6	117.1	0.67 (0.54, 0.83)	<0.01	2009-2013	1.78 (1.18, 2.86)	<0.01	2013-2023	0.23 (0.01, 0.4)	0.04	–	–	–
Philippines	LMIC	15.2	21.5	2.7 (2.57, 2.83)	<0.01	2009-2023	2.7 (2.57, 2.83)	<0.01	–	–	–	–	–	–
Singapore	HIC	28.6	33.1	1.13 (0.86, 1.41)	<0.01	2009-2023	1.13 (0.86, 1.41)	<0.01	–	–	–	–	–	–
South Korea	HIC	35.2	49.6	2.82 (2.5, 3.14)	<0.01	2009-2023	2.82 (2.5, 3.14)	<0.01	–	–	–	–	–	–
Thailand	UMIC	13.3	19.9	2.88 (2.61, 3.1)	<0.01	2009-2018	3.44 (3.17, 3.95)	<0.01	2018-2023	1.87 (0.16, 2.52)	0.04	–	–	–
Vietnam	LMIC	12.2	19.6	3.34 (2.97, 3.84)	<0.01	2009-2021	3.62 (2.83, 6.6)	<0.01	2021-2023	1.63 (-1.17, 3.81)	0.12	–	–	–
**Europe and Central Asia**														
Austria	HIC	132.5	138.0	0.41 (0.26, 0.56)	<0.01	2009-2023	0.41 (0.26, 0.56)	<0.01	–	–	–	–	–	–
Azerbaijan*	UMIC	75.2	121.9	3.38 (3.17, 3.62)	<0.01	2009-2014	7.04 (5.96, 7.92)	<0.01	2014-2023	1.4 (1.02, 1.78)	<0.01	–	–	–
Belarus*	UMIC	80.4	76.4	-0.25 (-0.39, -0.08)	<0.01	2009-2012	-0.39 (-1.43, 1.03)	0.53	2012-2016	-2.19 (-2.61, 0.38)	0.07	2016-2023	0.94 (0.59, 1.44)	0.0212
Belgium	HIC	151.0	144.6	-0.14 (-0.39, 0.11)	0.26	2009-2023	-0.14 (-0.39, 0.11)	0.26	–	–	–	–	–	–
Bosnia and Herzegovina*	UMIC	69.6	64.7	-0.59 (-0.7, -0.49)	<0.01	2009-2013	-1.82 (-2.61, -1.41)	<0.01	2013-2020	-0.58 (-0.87, -0.3)	<0.01	2020-2023	1.04 (0.4, 2.01)	<0.01
Bulgaria	UMIC	123.0	136.9	0.75 (0.65, 0.82)	<0.01	2009-2014	0.16 (-0.32, 0.42)	0.22	2014-2021	1.54 (1.41, 1.84)	<0.01	2021-2023	-0.55 (-1.3, 0.27)	0.13
Croatia*	HIC	93.5	104.3	0.8 (0.7, 0.95)	<0.01	2009-2011	0.11 (-0.7, 1.43)	0.70	2011-2015	1.71 (0.29, 2.1)	0.01	2015-2023	0.52 (0.18, 0.98)	0.03
Czech Republic	HIC	136.1	119.3	-0.94 (-1.2, -0.74)	<0.01	2009-2017	-1.11 (-2.24, -0.73)	0.03	2017-2021	0.24 (-1.05, 0.76)	0.60	2021-2023	-2.59 (-4.48, -0.56)	<0.01
Denmark	HIC	131.2	135.4	0.22 (0.07, 0.35)	<0.01	2009-2013	-0.82 (-1.84, -0.25)	<0.01	2013-2020	1.09 (0.84, 1.66)	<0.01	2020-2023	-0.42 (-1.67, 0.29)	0.25
Estonia*	HIC	104.3	106.2	0.16 (0.09, 0.24)	<0.01	2009-2013	0.89 (0.58, 1.29)	<0.01	2013-2019	-0.4 (-0.72, -0.22)	<0.01	2019-2023	0.25 (-0.05, 0.92)	0.08
Finland	HIC	152.4	158.1	0.33 (0.06, 0.54)	0.01	2009-2015	-0.32 (-1.61, 0.24)	0.25	2015-2021	1.79 (1.28, 2.81)	<0.01	2021-2023	-2.06 (-4, -0.15)	0.03

France	HIC	145.5	139.0	-0.43 (-0.58, -0.29)	<0.01	2009-2023	-0.43 (-0.58, -0.29)	<0.01	–	–	–	–	–	–
Georgia*	UMIC	74.9	122.9	3.78 (3.56, 4.08)	<0.01	2009-2012	6.56 (5.02, 9.71)	<0.01	2012-2023	3.03 (2.71, 3.29)	<0.01	–	–	–
Germany	HIC	179.6	172.9	-0.26 (-0.53, -0.07)	0.01	2009-2017	-0.51 (-1.46, -0.16)	0.02	2017-2021	1.43 (0.42, 2.03)	0.02	2021-2023	-2.62 (-4.47, -0.71)	0.02
Greece	HIC	94.4	91.4	-0.22 (-0.3, -0.15)	<0.01	2009-2017	-0.82 (-1.02, -0.66)	<0.01	2017-2023	0.57 (0.34, 0.91)	<0.01	–	–	–
Hungary	HIC	116.0	111.9	-0.27 (-0.37, -0.17)	<0.01	2009-2014	-0.6 (-1.29, -0.29)	<0.01	2014-2021	0.25 (0.09, 0.65)	<0.01	2021-2023	-1.25 (-2.02, -0.42)	<0.01
Ireland	HIC	167.7	173.4	0.36 (0.17, 0.57)	<0.01	2009-2017	-0.22 (-0.9, 0.81)	0.16	2017-2021	1.82 (-0.59, 2.29)	0.13	2021-2023	-0.25 (-1.58, 1.39)	0.79
Italy	HIC	139.9	147.4	0.34 (0.24, 0.44)	<0.01	2009-2016	-0.54 (-0.95, -0.27)	0.03	2016-2021	1.57 (-0.5, 2.04)	0.10	2021-2023	0.39 (-0.45, 1.33)	0.21
Kazakhstan*	UMIC	72.6	69.3	-0.24 (-0.4, -0.08)	<0.01	2009-2023	-0.24 (-0.4, -0.08)	<0.01	–	–	–	–	–	–
Latvia*	HIC	93.8	106.9	1.08 (0.86, 1.29)	<0.01	2009-2015	1.48 (1.11, 3.04)	<0.01	2015-2023	0.78 (-0.61, 1.03)	0.09	–	–	–
Lithuania*	HIC	91.4	99.4	0.62 (0.42, 0.83)	<0.01	2009-2011	-0.59 (-1.48, 1.09)	0.51	2011-2021	1.47 (1.32, 2.05)	<0.01	2021-2023	-2.33 (-3.86, -0.73)	<0.01
Netherlands	HIC	183.9	168.9	-0.58 (-0.84, -0.34)	<0.01	2009-2017	-1.11 (-2.84, -0.63)	0.021	2017-2023	0.13 (-0.57, 2.14)	0.61	–	–	–
North Macedonia*	UMIC	111.6	143.5	1.78 (1.71, 1.84)	<0.01	2009-2018	2.16 (2.06, 2.28)	<0.01	2018-2023	1.1 (0.78, 1.34)	<0.01	–	–	–
Norway	HIC	179.7	179.2	-0.05 (-0.27, 0.11)	0.59	2009-2017	-0.43 (-0.91, -0.14)	<0.01	2017-2021	2.46 (1.66, 2.99)	<0.01	2021-2023	-3.42 (-5.02, -1.67)	<0.01
Poland	HIC	126.4	123.20	-0.19 (-0.27, -0.12)	<0.01	2009-2018	-1.2 (-1.33, -1.08)	<0.01	2018-2023	1.65 (1.39, 1.9)	<0.01	–	–	–
Portugal	HIC	92.2	97.9	0.43 (0.3, 0.59)	<0.01	2009-2014	-0.26 (-1.51, 0.26)	0.29	2014-2023	0.82 (0.6, 1.44)	<0.01	–	–	–
Romania	HIC	113.3	109.9	-0.19 (-0.3, -0.08)	<0.01	2009-2011	-0.87 (-1.42, -0.05)	0.03	2011-2021	0.09 (0.02, 0.5)	0.03	2021-2023	-0.92 (-1.77, -0.17)	<0.01
Russia	UMIC	93.3	94.0	0.08 (-0.05, 0.21)	0.23	2009-2023	0.08 (-0.05, 0.21)	0.23	–	–	–	–	–	–
Serbia*	UMIC	139.0	139.8	0.11 (0.01, 0.24)	0.03	2009-2012	-1.77 (-2.74, -1.05)	<0.01	2012-2017	-0.51 (-0.95, 1.24)	0.24	2017-2023	1.59 (1.27, 2.14)	<0.01
Slovakia	HIC	126.0	116.0	-0.56 (-0.87, -0.25)	<0.01	2009-2023	-0.56 (-0.87, -0.25)	<0.01	–	–	–	–	–	–
Slovenia*	HIC	111.4	107.4	-0.24 (-0.32, -0.16)	<0.01	2009-2016	-1.59 (-1.79, -1.4)	<0.01	2016-2023	1.13 (0.94, 1.32)	<0.01	–	–	–
Spain	HIC	128.3	123.9	-0.17 (-0.53, 0.32)	0.23	2009-2021	0.07 (-0.76, 3.04)	0.41	2021-2023	-1.58 (-4.37, 0.32)	0.21	–	–	–
Sweden	HIC	148.3	137.8	-0.6 (-0.72, -0.5)	<0.01	2009-2017	-0.5 (-1.07, -0.35)	<0.01	2017-2021	0.25 (-0.19, 0.58)	0.32	2021-2023	-2.66 (-3.51, -1.61)	<0.01
Switzerland	HIC	129.4	122.2	-0.38 (-0.56, -0.21)	<0.01	2009-2017	-0.67 (-1.63, -0.47)	<0.01	2017-2023	0.01 (-0.31, 1.04)	0.89	–	–	–
Turkey	UMIC	134.9	107.2	-1.6 (-1.94, -1.21)	<0.01	2009-2011	0.04 (-3.09, 2.99)	0.85	2011-2017	-5.36 (-6.91, -4.56)	<0.01	2017-2023	1.75 (0.8, 3.15)	<0.01
Ukraine	LMIC	74.1	69.9	-1.52 (-2.58, -0.46)	<0.01	2009-2023	-1.52 (-2.58, -0.46)	<0.01	–	–	–	–	–	–
United Kingdom	HIC	161.2	142.8	-0.97 (-1.22, -0.8)	<0.01	2009-2017	-0.96 (-1.97, -0.67)	<0.01	2017-2021	0.76 (-0.07, 1.34)	0.07	2021-2023	-4.4 (-6.2, -2.44)	<0.01
Uzbekistan*	LMIC	93.1	117.5	1.63 (1.42, 1.83)	<0.01	2009-2012	0.41 (-1.5, 1.66)	0.45	2012-2018	4.13 (3.61, 5.03)	<0.01	2018-2023	-0.56 (-1.45, 0.08)	0.08
**Latin America and Caribbean**														
Argentina	UMIC	92.0	75.9	-1.44 (-1.59, -1.3)	<0.01	2009-2015	0.44 (0.1, 0.78)	0.02	2015-2020	-4.79 (-5.31, -4.39)	<0.01	2020-2023	0.53 (-0.43, 1.46)	0.18
Bolivia*	LMIC	27.9	31.5	0.74 (0.54, 0.88)	<0.01	2009-2020	1.21 (1.06, 1.42)	<0.01	2020-2023	-0.99 (-2.99, -0.08)	0.04	–	–	–

Brazil	UMIC	54.7	63.3	1 (0.91, 1.1)	<0.01	2009-2013	1.57 (1.24, 2.24)	<0.01	2013-2017	-0.36 (-0.65, 0.14)	0.16	2017-2023	1.53 (1.3, 1.9)	<0.01
Chile	HIC	123.1	122.2	-0.56 (-1.28, 0.18)	0.13	2009-2023	-0.56 (-1.28, 0.18)	0.13	–	–	–	–	–	–
Colombia	UMIC	36.4	38.8	0.48 (0.28, 0.68)	<0.01	2009-2014	0.46 (-0.19, 1.39)	0.16	2014-2020	-2.27 (-2.97, -1.79)	<0.01	2020-2023	6.24 (5.13, 7.32)	<0.01
Costa Rica*	UMIC	49.8	65.1	2.06 (1.9, 2.23)	<0.01	2009-2023	2.06 (1.9, 2.23)	<0.01	–	–	–	–	–	–
Dominican Republic*	UMIC	22.7	22.9	0.03 (-0.14, 0.16)	0.76	2009-2016	0.44 (-0.47, 1.15)	0.23	2016-2020	1.1 (0.57, 1.47)	<0.01	2020-2023	-2.33 (-3.46, -1.47)	<0.01
Ecuador*	UMIC	34.7	39.1	0.77 (0.52, 0.98)	<0.01	2009-2018	0.37 (-0.98, 0.75)	0.35	2018-2023	1.49 (0.73, 3.31)	0.01	–	–	–
El Salvador*	LMIC	42.6	47.3	0.71 (0.6, 0.84)	<0.01	2009-2020	0.85 (0.76, 1.35)	<0.01	2020-2023	0.19 (-0.75, 0.72)	0.49	–	–	–
Guatemala*	UMIC	52.5	60.7	1.11 (1.01, 1.22)	<0.01	2009-2023	1.11 (1.01, 1.22)	<0.01	–	–	–	–	–	–
Honduras*	LMIC	13.3	15.4	1.02 (0.89, 1.14)	<0.01	2009-2014	0.64 (-0.36, 1.03)	0.12	2014-2018	2.74 (2.04, 3.13)	<0.01	2018-2023	0.05 (-0.51, 0.38)	0.87
Mexico	UMIC	142.1	142.8	0.21 (-0.23, 0.65)	0.26	2009-2014	-1.62 (-4.84, -0.32)	0.01	2014-2023	1.25 (0.68, 2.64)	<0.01	–	–	–
Panama*	HIC	50.5	68.7	2.19 (2, 2.37)	<0.01	2009-2013	1.5 (-0.29, 2.22)	0.07	2013-2017	4.82 (3.84, 5.44)	<0.01	2017-2023	0.94 (0.35, 1.35)	<0.01
Paraguay*	UMIC	30.0	35.6	0.75 (0.24, 1.27)	<0.01	2009-2016	2.91 (1.8, 4.67)	<0.01	2016-2023	-1.36 (-2.97, -0.33)	<0.01	–	–	–
Peru	UMIC	38.3	41.9	0.59 (-0.34, 1.5)	0.166	2009-2023	0.59 (-0.34, 1.5)	0.16	–	–	–	–	–	–
Uruguay*	HIC	95.8	100.7	0.33 (0.24, 0.4)	<0.01	2009-2015	1.06 (0.83, 1.27)	<0.01	2015-2020	-0.81 (-1.17, -0.52)	<0.01	2020-2023	0.79 (0.24, 1.69)	<0.01
**Middle East and North Africa**														
Algeria*	LMIC	145.6	156.3	0.49 (0.43, 0.54)	<0.01	2009-2019	0.79 (0.72, 0.87)	<0.01	2019-2023	-0.27 (-0.67, 0)	0.05	–	–	–
Egypt	LMIC	39.2	45.0	0.9 (0.68, 1.21)	<0.01	2009-2013	2.34 (1.34, 4.52)	<0.01	2013-2023	0.34 (-0.12, 0.6)	0.10	–	–	–
Iraq*	UMIC	75.3	79.4	0.07 (-1.13, 1.37)	0.90	2009-2015	-3.3 (-12.28, -0.13)	0.04	2015-2023	2.67 (0.47, 10.71)	0.02	–	–	–
Israel	HIC	76.8	72.0	-0.46 (-0.71, -0.24)	<0.01	2009-2012	0.63 (-0.44, 2.75)	0.28	2012-2023	-0.75 (-1.78, -0.58)	<0.01	–	–	–
Jordan*	UMIC	61.2	59.4	-0.53 (-1.22, 0.29)	0.14	2009-2013	3.61 (0.59, 10.18)	0.02	2013-2023	-2.14 (-3.51, -1.37)	<0.01	–	–	–
Kuwait*	HIC	96.0	88.1	-0.19 (-0.61, 0.15)	0.26	2009-2019	-0.74 (-2.6, -0.26)	0.03	2019-2023	1.22 (-0.3, 4.27)	0.13	–	–	–
Lebanon*	LMIC	114.0	169.9	2.68 (2.16, 3.2)	<0.01	2009-2023	2.68 (2.16, 3.2)	<0.01	–	–	–	–	–	–
Morocco	LMIC	44.1	55.9	1.59 (1.45, 1.73)	<0.01	2009-2019	1.93 (1.77, 2.21)	<0.01	2019-2023	0.74 (-0.43, 1.32)	0.14	–	–	–
Oman*	HIC	64.2	74.5	1.47 (0.73, 2.2)	<0.01	2009-2023	1.47 (0.73, 2.2)	<0.01	–	–	–	–	–	–
Qatar*	HIC	45.8	69.3	3.04 (2.73, 3.33)	<0.01	2009-2018	2.45 (1.39, 2.79)	0.01	2018-2023	4.13 (3.3, 6.13)	<0.01	–	–	–
Saudi Arabia	HIC	107.3	115.1	0.49 (0.15, 0.8)	<0.01	2009-2014	1.13 (0.45, 3.76)	<0.01	2014-2023	0.13 (-2.1, 0.46)	0.67	–	–	–
Tunisia*	LMIC	84.0	93.5	0.74 (0.64, 0.81)	<0.01	2009-2017	0.38 (0.13, 0.52)	0.01	2017-2021	1.67 (0.63, 1.89)	<0.01	2021-2023	0.31 (-0.43, 1.22)	0.25
United Arab Emirates	HIC	42.5	55.7	2.18 (1.71, 2.72)	<0.01	2009-2011	-4.62 (-7.69, 0.05)	0.05	2011-2016	5.18 (3.9, 7.32)	<0.01	2016-2023	2.07 (0.14, 2.73)	0.04
**North America**														
Canada	HIC	115.1	108.7	-0.29 (-0.58, 0.01)	0.06	2009-2023	-0.29 (-0.58, 0.01)	0.06	–	–	–	–	–	–

USA	HIC	132.3	132.4	-0.02 (-0.31, 0.34)	0.80	2009-2017	-0.62 (-1.64, 0.56)	0.06	2017-2021	2.15 (-1.07, 3.05)	0.10	2021-2023	-1.91 (-3.94, 1.27)	0.17
**South Asia**														
Bangladesh*	LMIC	2.0	2.8	2.45 (2.09, 2.8)	<0.01	2009-2011	11.52 (8.1, 14.17)	<0.01	2011-2021	3.4 (3.02, 3.74)	<0.01	2021-2023	-10.12 (-12.21, -7.93)	<0.01
India	LMIC	4.0	7.2	4.35 (3.94, 4.75)	<0.01	2009-2023	4.35 (3.94, 4.75)	<0.01	–	–	–	–	–	–
Pakistan*	LMIC	3.8	7.7	5.24 (4.67, 5.66)	<0.01	2009-2015	3.72 (0.8, 4.87)	0.02	2015-2019	9.92 (7.4, 11.4)	<0.01	2019-2023	2.99 (-1.05, 4.7)	0.11
Sri Lanka*	LMIC	7.6	9.9	1.81 (1.45, 2.19)	<0.01	2009-2016	5.27 (4.65, 6.66)	<0.01	2016-2021	2.34 (1.09, 3.59)	<0.01	2021-2023	-10.61 (-13.15, -7.01)	<0.01
**Sub-Saharan Africa**														
Angola*	LMIC	6.1	5.4	-0.95 (-1.27, -0.65)	<0.01	2009-2018	0.51 (0.04, 1.1)	0.03	2018-2023	-3.51 (-5, -2.48)	<0.01	–	–	–
Cameroon*	LMIC	4.9	4.5	-0.38 (-0.84, -0.06)	0.02	2009-2019	0.8 (0.38, 1.37)	<0.01	2019-2023	-3.27 (-6.23, -1.8)	<0.01	–	–	–
Côte d'Ivoire*	LMIC	5.4	6.1	1.02 (0.61, 1.31)	<0.01	2009-2019	1.81 (1.47, 2.38)	<0.01	2019-2023	-0.92 (-3.66, 0.31)	0.11	–	–	–
Ethiopia*	LMIC	3.2	2.7	-1.16 (-1.64, -0.84)	<0.01	2009-2020	-1.64 (-3.85, 1.5)	0.07	2020-2023	0.66 (-1.68, 3.5)	0.74	–	–	–
Ghana*	LMIC	10.4	11.6	0.88 (0.5, 1.19)	<0.01	2009-2019	2.17 (1.75, 2.69)	<0.01	2019-2023	-2.28 (-4.79, -0.86)	<0.01	–	–	–
Kenya*	LMIC	7.2	9.3	1.85 (1.52, 2.12)	<0.01	2009-2013	-0.55 (-2.67, 0.71)	0.31	2013-2020	4.03 (3.47, 5.27)	<0.01	2020-2023	0.05 (-2.85, 1.54)	0.93
Nigeria	LMIC	14.7	10.3	-3.26 (-4.61, -1.9)	<0.01	2009-2023	-3.26 (-4.61, -1.9)	<0.01	–	–	–	–	–	–
South Africa	UMIC	31.4	37.1	1.22 (1.04, 1.38)	<0.01	2009-2013	2.94 (2.43, 4.15)	<0.01	2013-2021	1.65 (1.38, 1.87)	<0.01	2021-2023	-3.8 (-5.02, -1.93)	<0.01
Tanzania*	LMIC	6.5	7.4	1.07 (0.78, 1.4)	<0.01	2009-2019	1.66 (1.31, 2.75)	<0.01	2019-2023	-0.4 (-3.01, 0.87)	0.52	–	–	–
Uganda*	LMIC	5.5	5.8	0.57 (0.25, 0.87)	<0.01	2009-2017	1.3 (0.86, 2.32)	<0.01	2017-2023	-0.4 (-1.98, 0.26)	0.22	–	–	–

Legend: Annual Average Percentage Change (AAPC), Annual Percentage Change (APC) and 95% Confidence Interval (CI) estimated using joinpoint regression models are shown. Original units and the description for the used indicator are reported in Supplementary Table 3. The results represent the percentage change in the unhealthy food sales per capita for each identified segment and for the entire analysis period (2009 to 2023), by country. Crude metrics for the first (2009) and last (2023) time points are reported. Two-tailed t-test was used to test whether AAPC and APC was statistically different from zero, with no adjustment made for multiple comparisons. LMICs=Low-and-middle-income countries; UMICs=Upper-and-middle-income countries; HICs=High-income countries. *Modelled data used for some or all unhealthy food categories included in the analysis.

Supplementary Table 10. Percentage of unhealthy food sales for selected food categories from chain retailers from 2009 to 2023, by country: joinpoint regression analysis.

	Income status	Percentage of unhealthy food sales from chain retail*		AAPC		Segment 1			Segment 2			Segment 3		
		2009	2023	AAPC (95% CI)	p-value	Years	APC (95% CI)	p-value	Years	APC (95% CI)	p-value	Years	APC (95% CI)	p-value
**East Asia and Pacific**														
Australia	HIC	95.5	95.7	0.04 (0.02, 0.07)	<0.001	2009-2023	0.04 (0.02, 0.07)	<0.001	–	–	–	–	–	–
Cambodia	LMIC	3.9	18.1	10.39 (8.06, 12.17)	<0.001	2009-2012	25.6 (11.89, 36.37)	<0.001	2012-2023	6.57 (1.65, 8.33)	0.03	–	–	–
China	UMIC	75.4	83.2	0.83 (0.74, 0.93)	<0.001	2009-2016	1.3 (1.09, 1.61)	<0.001	2016-2023	0.36 (0.06, 0.56)	0.03	–	–	–
Indonesia	LMIC	42.2	41.9	0.7 (0.02, 1.41)	0.05	2009-2023	0.7 (0.02, 1.41)	0.05	–	–	–	–	–	–
Japan	HIC	98.7	98.5	0 (-0.01, 0.02)	0.88	2009-2012	-0.14 (-0.21, -0.01)	0.02	2012-2023	0.04 (0.02, 0.09)	0.01	–	–	–
Laos	LMIC	32.0	42.1	1.44 (0.79, 1.92)	<0.001	2009-2012	6.06 (2.26, 8.66)	<0.001	2012-2023	0.22 (-0.86, 0.7)	0.62	–	–	–
Malaysia	UMIC	74.6	62.1	-0.37 (-1.02, 0.31)	0.27	2009-2023	-0.37 (-1.02, 0.31)	0.27	–	–	–	–	–	–
Myanmar	LMIC	64.2	61.8	0.15 (-0.24, 0.78)	0.38	2009-2012	-2.75 (-4.69, 0.31)	0.10	2012-2023	0.95 (0.42, 2.8)	0.02	–	–	–
New Zealand	HIC	89.9	92.4	0.18 (0.12, 0.23)	<0.001	2009-2012	0.42 (0.17, 0.69)	<0.001	2012-2023	0.12 (-0.13, 0.17)	0.09	–	–	–
Philippines	LMIC	61.3	63.9	0.72 (0.31, 1.41)	<0.001	2009-2012	-3.76 (-5.65, 0.06)	0.05	2012-2023	1.98 (1.42, 3.22)	0.00	–	–	–
Singapore	HIC	74.9	81.7	0.53 (0.44, 0.62)	<0.001	2009-2023	0.53 (0.44, 0.62)	<0.001	–	–	–	–	–	–
South Korea	HIC	88.2	91.4	0.41 (0.31, 0.52)	<0.001	2009-2023	0.41 (0.31, 0.52)	<0.001	–	–	–	–	–	–
Thailand	UMIC	68.5	72.4	0.37 (0.3, 0.43)	<0.001	2009-2012	1.38 (0.97, 1.68)	<0.001	2012-2018	0.61 (0.23, 0.77)	0.02	2018-2023	-0.51 (-0.83, -0.31)	<0.001
Vietnam	LMIC	5.2	18.8	8.78 (7.37, 9.82)	<0.001	2009-2012	16.32 (9.08, 22.1)	<0.001	2012-2023	6.8 (3.12, 7.9)	0.01	–	–	–
**Europe and Central Asia**														
Austria	HIC	93.9	95.2	0.1 (0.08, 0.11)	<0.001	2009-2023	0.1 (0.08, 0.11)	<0.001	–	–	–	–	–	–
Azerbaijan	UMIC	22.9	37.6	3.91 (3.52, 4.55)	<0.001	2009-2012	0.09 (-1.8, 3.53)	0.72	2012-2023	4.97 (4.46, 6.28)	<0.001	–	–	–
Belarus	UMIC	25.8	65.7	7.03 (6.42, 7.66)	<0.001	2009-2015	11.87 (10.01, 14.51)	<0.001	2015-2023	3.54 (2.07, 4.71)	<0.001	–	–	–
Belgium	HIC	92.2	93.2	0.09 (0.08, 0.1)	<0.001	2009-2012	0.05 (-0.01, 0.12)	0.12	2012-2016	0.2 (0.15, 0.28)	0.01	2016-2023	0.04 (0.01, 0.07)	0.02
Bosnia and Herzegovina	UMIC	39.0	59.0	3.08 (2.69, 3.62)	<0.001	2009-2013	6.51 (4.69, 9.84)	<0.001	2013-2016	-1.88 (-3.65, 0.97)	0.29	2016-2023	3.33 (2.42, 5.65)	<0.001
Bulgaria	UMIC	39.4	68.3	3.92 (3.77, 4.06)	<0.001	2009-2013	9.97 (9.42, 10.52)	<0.001	2013-2019	2.23 (1.95, 2.71)	<0.001	2019-2023	0.64 (-0.19, 1.2)	0.12
Croatia	HIC	82.1	90.1	0.65 (0.58, 0.73)	<0.001	2009-2014	1.07 (0.83, 1.48)	<0.001	2014-2020	0.61 (0.45, 1.1)	<0.001	2020-2023	0.04 (-0.3, 0.41)	0.58
Czech Republic	HIC	86.3	92.1	0.46 (0.41, 0.51)	<0.001	2009-2023	0.46 (0.41, 0.51)	<0.001	–	–	–	–	–	–
Denmark	HIC	94.3	95.9	0.14 (0.12, 0.16)	<0.001	2009-2015	0.21 (0.16, 0.33)	<0.001	2015-2023	0.08 (0, 0.12)	0.05	–	–	–

Estonia	HIC	88.2	92.8	0.33 (0.29, 0.37)	<0.001	2009-2023	0.33 (0.29, 0.37)	<0.001	–	–	–	–	–	–
Finland	HIC	97.8	97.9	0.04 (0.02, 0.06)	<0.001	2009-2023	0.04 (0.02, 0.06)	<0.001	–	–	–	–	–	–
France	HIC	88.9	92.1	0.2 (0.15, 0.24)	<0.001	2009-2012	0.54 (0.25, 0.73)	<0.001	2012-2023	0.11 (0.01, 0.15)	0.04	–	–	–
Georgia	UMIC	20.9	40.0	4.31 (3.59, 4.98)	<0.001	2009-2017	2.66 (-0.6, 3.87)	0.09	2017-2023	6.56 (4.74, 10.47)	<0.001	–	–	–
Germany	HIC	92.4	93.9	0.15 (0.13, 0.18)	<0.001	2009-2023	0.15 (0.13, 0.18)	<0.001	–	–	–	–	–	–
Greece	HIC	78.5	77.5	0.67 (0.21, 1.15)	0.00	2009-2023	0.67 (0.21, 1.15)	0.00	–	–	–	–	–	–
Hungary	HIC	81.6	89.9	0.68 (0.63, 0.73)	<0.001	2009-2012	1.72 (1.31, 2.02)	<0.001	2012-2023	0.4 (0.32, 0.45)	<0.001	–	–	–
Ireland	HIC	90.3	94.3	0.33 (0.3, 0.35)	<0.001	2009-2018	0.48 (0.44, 0.52)	<0.001	2018-2023	0.06 (-0.05, 0.15)	0.21	–	–	–
Italy	HIC	87.8	88.2	0.04 (0.02, 0.07)	<0.001	2009-2023	0.04 (0.02, 0.07)	<0.001	–	–	–	–	–	–
Kazakhstan	UMIC	10.8	33.6	9.21 (8.53, 9.89)	<0.001	2009-2016	5.42 (3.14, 6.93)	0.00	2016-2023	13.13 (11.54, 15.39)	<0.001	–	–	–
Latvia	HIC	89.0	94.7	0.46 (0.42, 0.5)	<0.001	2009-2012	0.45 (0.26, 0.73)	<0.001	2012-2015	1.05 (0.46, 1.23)	0.01	2015-2023	0.24 (0.15, 0.3)	0.00
Lithuania	HIC	73.9	88.7	1.12 (0.77, 1.4)	<0.001	2009-2012	2.43 (0.94, 3.89)	<0.001	2012-2023	0.77 (-0.53, 1.11)	0.08	–	–	–
Netherlands	HIC	90.5	95.1	0.31 (0.25, 0.35)	<0.001	2009-2012	0.62 (0.32, 0.84)	<0.001	2012-2023	0.22 (0.07, 0.27)	0.03	–	–	–
North Macedonia	UMIC	34.3	34.5	0.26 (0.11, 0.49)	0.00	2009-2012	-1.58 (-2.33, -0.26)	0.02	2012-2023	0.76 (0.58, 1.12)	<0.001	–	–	–
Norway	HIC	99.4	99.7	0.02 (0.01, 0.02)	<0.001	2009-2023	0.02 (0.01, 0.02)	<0.001	–	–	–	–	–	–
Poland	HIC	62.6	93.0	2.81 (2.69, 2.92)	<0.001	2009-2012	8.32 (7.55, 9)	<0.001	2012-2015	3.12 (2.15, 3.6)	<0.001	2015-2023	0.71 (0.45, 0.88)	<0.001
Portugal	HIC	85.1	87.7	0.26 (0.23, 0.32)	<0.001	2009-2012	-0.01 (-0.18, 0.27)	0.81	2012-2023	0.34 (0.29, 0.49)	<0.001	–	–	–
Romania	HIC	31.5	60.4	4.53 (3.94, 5)	<0.001	2009-2012	10.42 (6.58, 12.92)	<0.001	2012-2023	2.98 (2.17, 3.49)	0.00	–	–	–
Russia	UMIC	51.2	77.0	3.04 (2.89, 3.2)	<0.001	2009-2013	3.65 (2.73, 4.34)	<0.001	2013-2017	5.47 (4.61, 6.36)	<0.001	2017-2023	1.06 (0.62, 1.44)	0.00
Serbia	UMIC	42.8	72.1	3.92 (3.71, 4.2)	<0.001	2009-2020	4.18 (3.83, 5.21)	<0.001	2020-2023	2.97 (1.84, 4.13)	<0.001	–	–	–
Slovakia	HIC	95.8	97.9	0.17 (0.14, 0.2)	<0.001	2009-2014	0.35 (0.27, 0.46)	<0.001	2014-2019	-0.03 (-0.19, 0.05)	0.30	2019-2023	0.18 (0.08, 0.32)	<0.001
Slovenia	HIC	95.8	97.0	0.1 (0.09, 0.11)	<0.001	2009-2017	0.19 (0.17, 0.22)	<0.001	2017-2023	-0.01 (-0.05, 0.02)	0.34	–	–	–
Spain	HIC	95.4	93.2	-0.04 (-0.12, 0.1)	0.69	2009-2012	-0.61 (-1.04, 0.04)	0.07	2012-2023	0.12 (-0.02, 0.54)	0.07	–	–	–
Sweden	HIC	95.3	96.1	0.06 (0.06, 0.07)	<0.001	2009-2017	0.05 (0.01, 0.06)	0.01	2017-2023	0.09 (0.07, 0.12)	<0.001	–	–	–
Switzerland	HIC	98.6	98.1	-0.03 (-0.03, -0.02)	<0.001	2009-2012	-0.12 (-0.15, -0.07)	<0.001	2012-2018	-0.02 (-0.05, 0.01)	0.12	2018-2023	0.03 (0.01, 0.07)	0.02
Turkey	UMIC	25.2	73.1	6.92 (6.03, 7.84)	<0.001	2009-2023	6.92 (6.03, 7.84)	<0.001	–	–	–	–	–	–
Ukraine	LMIC	49.5	77.1	3.22 (2.97, 3.48)	<0.001	2009-2023	3.22 (2.97, 3.48)	<0.001	–	–	–	–	–	–
United Kingdom	HIC	92.5	93.0	0.07 (0.02, 0.11)	0.01	2009-2023	0.07 (0.02, 0.11)	0.01	–	–	–	–	–	–
Uzbekistan	LMIC	1.1	11.2	18.05 (16.3, 19.98)	<0.001	2009-2015	12.64 (3.47, 17.33)	0.01	2015-2023	22.27 (19.03, 31.82)	<0.001	–	–	–
**Latin America and Caribbean**														

Argentina	UMIC	50.3	50.6	0.44 (0.09, 0.79)	0.02	2009-2023	0.44 (0.09, 0.79)	0.02	–	–	–	–	–	–
Bolivia	LMIC	9.9	14.3	2.56 (2.25, 2.96)	<0.001	2009-2012	6.03 (3.87, 8.09)	<0.001	2012-2015	-0.01 (-1.28, 1.6)	0.75	2015-2023	2.26 (1.65, 3.87)	<0.001
Brazil	UMIC	60.8	59.0	-0.09 (-0.22, 0.14)	0.46	2009-2012	-0.92 (-1.51, 0.15)	0.11	2012-2023	0.14 (-0.67, 0.91)	0.17	–	–	–
Chile	HIC	55.7	66.1	1.28 (1.03, 1.51)	<0.001	2009-2017	0.89 (-0.08, 1.41)	0.06	2017-2020	3.16 (0.63, 4.01)	0.01	2020-2023	0.46 (-0.76, 2)	0.29
Colombia	UMIC	38.2	42.3	1.25 (0.78, 2.02)	<0.001	2009-2012	-3.26 (-5.45, 0.79)	0.15	2012-2023	2.52 (1.88, 4.21)	0.00	–	–	–
Costa Rica	UMIC	63.5	60.6	-0.16 (-0.26, 0.02)	0.09	2009-2012	-0.97 (-1.52, -0.12)	0.01	2012-2023	0.07 (-0.08, 0.57)	0.25	–	–	–
Dominican Republic	UMIC	47.8	52.1	0.99 (0.74, 1.24)	<0.001	2009-2023	0.99 (0.74, 1.24)	<0.001	–	–	–	–	–	–
Ecuador	UMIC	63.6	57.6	-0.21 (-0.64, 0.48)	0.58	2009-2012	-4.33 (-6.44, -0.61)	0.01	2012-2023	0.94 (0.39, 2.43)	0.01	–	–	–
El Salvador	LMIC	76.1	73.9	0.2 (-0.04, 0.44)	0.09	2009-2023	0.2 (-0.04, 0.44)	0.09	–	–	–	–	–	–
Guatemala	UMIC	22.2	22.7	0.22 (0.1, 0.33)	<0.001	2009-2017	-0.04 (-0.52, 0.14)	0.60	2017-2023	0.56 (0.29, 1.13)	<0.001	–	–	–
Honduras	LMIC	42.4	43.8	0.23 (0.07, 0.39)	0.01	2009-2016	1.38 (1.04, 2.12)	<0.001	2016-2020	0.22 (-0.34, 1.18)	0.30	2020-2023	-2.39 (-3.27, -1.43)	<0.001
Mexico	UMIC	56.9	50.3	-0.33 (-0.75, 0.33)	0.38	2009-2012	-3.36 (-5.39, -0.18)	0.03	2012-2023	0.51 (-0.06, 2.42)	0.07	–	–	–
Panama	HIC	80.6	80.0	0.09 (-0.01, 0.22)	0.08	2009-2019	0.26 (0.1, 0.74)	0.03	2019-2023	-0.33 (-0.91, 0.13)	0.18	–	–	–
Paraguay	UMIC	44.6	37.9	-1.63 (-2.1, -1.17)	<0.001	2009-2018	0.29 (-0.42, 1.28)	0.38	2018-2023	-4.99 (-7.34, -3.44)	<0.001	–	–	–
Peru	UMIC	22.4	25.2	1.03 (0.71, 1.39)	<0.001	2009-2012	-1.08 (-2.43, 1.03)	0.34	2012-2020	2.5 (2.19, 4.58)	<0.001	2020-2023	-0.74 (-2.35, 1.09)	0.46
Uruguay	HIC	74.5	67.0	-0.35 (-0.76, 0.35)	0.27	2009-2012	-6.96 (-8.8, -2.8)	<0.001	2012-2023	1.54 (0.92, 2.55)	<0.001	–	–	–
**Middle East and North Africa**														
Algeria	LMIC	38.5	42.0	0.98 (0.54, 1.74)	<0.001	2009-2012	-4.61 (-6.65, -0.35)	0.03	2012-2023	2.56 (1.91, 3.84)	<0.001	–	–	–
Egypt	LMIC	44.4	46.5	0.81 (0.17, 1.53)	0.02	2009-2012	-6.99 (-9.98, -2.85)	<0.001	2012-2017	6.06 (4.35, 11.07)	<0.001	2017-2023	0.61 (-2.16, 1.98)	0.53
Iraq	UMIC	11.5	19.7	3.5 (2.72, 4.5)	<0.001	2009-2012	12.6 (6.59, 18.7)	<0.001	2012-2016	-5.44 (-10.34, -2.18)	0.00	2016-2023	5.12 (3.23, 9.26)	<0.001
Israel	HIC	77.7	81.9	0.44 (0.37, 0.53)	<0.001	2009-2017	0.56 (0.46, 0.95)	<0.001	2017-2023	0.28 (-0.13, 0.44)	0.13	–	–	–
Jordan	UMIC	44.3	44.1	0.03 (-0.32, 0.65)	0.77	2009-2012	-3.57 (-5.14, -0.28)	0.03	2012-2023	1.03 (0.51, 2.43)	0.01	–	–	–
Kuwait	HIC	51.2	67.4	1.58 (1.04, 1.96)	<0.001	2009-2012	4.79 (1.86, 6.88)	<0.001	2012-2023	0.72 (-0.42, 1.12)	0.11	–	–	–
Lebanon	LMIC	65.9	65.1	-0.03 (-0.12, 0.14)	0.68	2009-2012	-1.05 (-1.47, -0.12)	0.02	2012-2023	0.25 (0.11, 0.64)	0.01	–	–	–
Morocco	LMIC	45.2	37.3	-0.06 (-0.9, 0.81)	0.91	2009-2023	-0.06 (-0.9, 0.81)	0.91	–	–	–	–	–	–
Oman	HIC	77.3	73.1	0.15 (-0.16, 0.46)	0.32	2009-2023	0.15 (-0.16, 0.46)	0.32	–	–	–	–	–	–
Qatar	HIC	81.7	80.8	0.09 (-0.08, 0.37)	0.27	2009-2012	-1.6 (-2.46, -0.07)	0.03	2012-2023	0.55 (0.33, 1.15)	0.00	–	–	–
Saudi Arabia	HIC	57.9	76.6	1.74 (1.42, 2.08)	<0.001	2009-2015	2.67 (2.01, 4.39)	<0.001	2015-2023	1.05 (-0.11, 1.47)	0.06	–	–	–
Tunisia	LMIC	16.5	33.8	5.91 (5.21, 6.64)	<0.001	2009-2012	0.8 (-2.63, 5.14)	0.59	2012-2016	13.11 (10.14, 18)	<0.001	2016-2023	4.18 (2.14, 5.53)	0.01
United Arab Emirates	HIC	90.0	92.9	0.13 (0.04, 0.22)	0.01	2009-2016	0.49 (0.3, 0.78)	<0.001	2016-2023	-0.23 (-0.52, -0.05)	0.01	–	–	–

**North America**														
Canada	HIC	86.2	86.1	0.06 (0.01, 0.11)	0.02	2009-2023	0.06 (0.01, 0.11)	0.02	–	–	–	–	–	–
USA	HIC	91.9	95.5	0.22 (0.15, 0.27)	<0.001	2009-2012	0.65 (0.26, 0.92)	<0.001	2012-2023	0.1 (-0.04, 0.16)	0.10	–	–	–
**South Asia**														
Bangladesh	LMIC	18.1	45.8	4.68 (1.76, 6.82)	0.00	2009-2012	25.43 (7.05, 39.35)	<0.001	2012-2023	-0.36 (-5.48, 1.82)	0.55	–	–	–
India	LMIC	14.4	16.0	1.81 (1.02, 3.1)	<0.001	2009-2012	-3.91 (-7.68, 2.05)	0.33	2012-2023	3.43 (2.36, 7.04)	0.01	–	–	–
Pakistan	LMIC	10.3	23.4	5.9 (5.5, 6.26)	<0.001	2009-2012	8.33 (6.1, 10.38)	<0.001	2012-2020	4.48 (2.34, 4.82)	<0.001	2020-2023	7.31 (5.38, 8.87)	<0.001
Sri Lanka	LMIC	19.3	24.1	1.89 (1.67, 2.1)	<0.001	2009-2023	1.89 (1.67, 2.1)	<0.001	–	–	–	–	–	–
**Sub-Saharan Africa**														
Angola	LMIC	5.6	8.1	1.06 (-0.74, 2.87)	0.19	2009-2018	4.78 (2.78, 10.07)	<0.001	2018-2023	-5.32 (-14.22, -0.6)	0.03	–	–	–
Cameroon	LMIC	34.1	44.7	1.91 (1.74, 2.22)	<0.001	2009-2012	0.63 (-0.18, 2.12)	0.10	2012-2023	2.27 (1.87, 3.29)	<0.001	–	–	–
Côte d'Ivoire	LMIC	33.4	31.0	-0.03 (-0.52, 0.57)	0.97	2009-2020	1.2 (0.71, 2.19)	<0.001	2020-2023	-4.42 (-6.98, -1)	0.00	–	–	–
Ethiopia	LMIC	0.8	2.7	7.3 (4.11, 9.84)	<0.001	2009-2012	27.46 (8.68, 43.92)	<0.001	2012-2023	2.38 (-4.69, 4.77)	0.31	–	–	–
Ghana	LMIC	4.9	18.2	8.77 (4.73, 12.39)	0.00	2009-2012	42.89 (13.92, 71)	<0.001	2012-2023	0.97 (-5.56, 4.13)	0.81	–	–	–
Kenya	LMIC	61.6	47.1	-1.81 (-2.16, -1.53)	<0.001	2009-2017	-0.98 (-1.4, -0.29)	0.02	2017-2020	-5.79 (-7.1, -3.86)	0.01	2020-2023	0.06 (-2.01, 1.68)	1.00
Nigeria	LMIC	1.9	6.9	9.62 (9.3, 9.92)	<0.001	2009-2015	11.34 (9.67, 12.19)	<0.001	2015-2019	16.29 (14.8, 18.35)	<0.001	2019-2023	0.95 (-0.26, 2.09)	0.11
South Africa	UMIC	80.6	83.1	0.24 (0.2, 0.28)	<0.001	2009-2018	-0.06 (-0.15, 0.01)	0.09	2018-2023	0.78 (0.62, 1.01)	<0.001	–	–	–
Tanzania	LMIC	7.0	16.2	5.32 (3.62, 6.72)	<0.001	2009-2012	23 (11.35, 31.32)	<0.001	2012-2023	0.96 (-1.28, 2.39)	0.37	–	–	–
Uganda	LMIC	6.9	9.2	2.23 (1.56, 2.99)	<0.001	2009-2014	5.97 (3.68, 10.19)	<0.001	2014-2023	0.21 (-1.27, 1.15)	0.75	–	–	–

Legend: Annual Average Percentage Change (AAPC), Annual Percentage Change (APC) and 95% Confidence Interval (CI) estimated using joinpoint regression models are shown. Original units and the description for the used indicator are reported in Supplementary Table 3. The results represent the percentage change in the unhealthy food sales from chain retailers for each identified segment and for the entire analysis period (2009 to 2023), by country. Crude metrics for the first (2009) and last (2023) time points are reported. Two-tailed t-test was used to test whether AAPC and APC was statistically different from zero, with no adjustment made for multiple comparisons. LMICs=Low-and-middle-income countries; UMICs=Upper-and-middle-income countries; HICs=High-income countries. [*]The percentage of unhealthy food sales (US$) for selected unhealthy food categories from chain outlets of total sales of these unhealthy food categories. Selected unhealthy food categories included: Baked goods, Breakfast Cereals, Confectionery, Sweet Spreads, Processed Meat, Seafood and Alternatives to Meat, Sauces, Dips and Condiments, Savoury Snacks, and Sweet Biscuits, Snack Bars and Fruit Snacks.

Supplementary Table 11. Grocery sales per capita (US$/year) through digital channels, overall and by income group and at national level, 2014–2023 based on joinpoint analysis including 27 countries.

	US$ per capita		Segment 1			Segment 2		
	2014	2023	Years	APC (95%CI)	p-value	Years	APC (95%CI)	p-value
**Overall (n=27)**	27.4	116.7	2014-2023	20.04 (14.91, 25.87)	<0.01	–	–	–
LMIC (n=3)	0.2	3.3	2014-2020	54.18 (47.21, 68.53)	<0.01	2020-2023	10.05 (-20.05, 24.7)	0.28
India	0.1	1.4	2014-2019	64.45 (49.5, 95.11)	<0.01	2019-2023	9.17 (-13.77, 23.97)	0.34
Indonesia	0.1	1.4	2014-2021	71.45 (52.38, 170.89)	<0.01	2021-2023	-29.8 (-63.18, 34.54)	0.41
Philippines	0.4	7.1	2014-2019	46.9 (40.15, 71.41)	<0.01	2019-2023	28.86 (4.92, 37.05)	0.01
UMIC (n=8)	3.8	29.1	2014-2018	10.57 (-15.47, 26.4)	0.36	2018-2023	44.15 (34.46, 69.68)	<0.01
Brazil	11.5	12.9	2014-2018	-8.67 (-34.43, 20.91)	0.23	2018-2023	12.97 (-16.79, 57.62)	0.11
China	4.4	26.8	2014-2020	27.86 (25.43, 33.52)	<0.01	2020-2023	14.23 (1.71, 20.63)	0.02
Colombia	2.4	37.1	2014-2023	39.17 (31.57, 47.04)	<0.01	–	–	–
Mexico	6.9	44.5	2014-2018	10.14 (-10.78, 21.7)	0.31	2018-2023	39.14 (28.96, 68.39)	<0.01
Russia	2.0	62.9	2014-2017	1.05 (-45.66, 59.6)	0.96	2017-2023	98.79 (64.96, 255.26)	<0.01
South Africa	0.6	11.1	2014-2017	15.34 (-7.88, 35.76)	0.21	2017-2023	59.02 (50.23, 81.64)	<0.01
Thailand	1.9	17.4	2014-2018	3.48 (-16.53, 17.08)	0.65	2018-2023	58.33 (44.46, 84.83)	<0.01
Turkey	0.7	19.8	2014-2017	15.16 (-9.88, 37.15)	0.21	2017-2023	62.28 (52.97, 87.53)	<0.01
HIC (n=16)	44.4	181.8	2014-2023	19.58 (14.5, 25.36)	<0.01	–	–	–
Australia	57.0	341.3	2014-2023	24.85 (20.64, 29.11)	<0.01	–	–	–
Canada	27.0	281.9	2014-2021	38.83 (32.26, 62.64)	<0.01	2021-2023	7.24 (-10.67, 32.19)	0.39
Chile	12.7	86.2	2014-2023	28.69 (17.75, 40.29)	<0.01	–	–	–
Czech Republic	5.1	164.1	2014-2017	91.06 (60.73, 176.82)	<0.01	2017-2023	35.33 (14.05, 44.12)	0.03
France	106.9	246.7	2014-2023	10.96 (7.81, 14.06)	<0.01	–	–	–
Germany	23.1	54.2	2014-2017	2.54 (-14.42, 21.32)	0.68	2017-2023	17.28 (-1.97, 40.19)	0.06
Italy	11.1	35.5	2014-2019	11.53 (0.48, 22.99)	0.04	2019-2023	19.51 (7.94, 33.86)	<0.01
Japan	103.1	153.1	2014-2020	1.89 (-2.19, 3.39)	0.26	2020-2023	9.57 (5.24, 15.99)	<0.01
Poland	3.8	17.1	2014-2018	8.92 (-1.95, 15.25)	0.09	2018-2023	26.29 (21.13, 36.38)	<0.01
Singapore	18.2	149.9	2014-2023	30.84 (24.69, 36.96)	<0.01	–	–	–
South Korea	38.3	77.1	2014-2020	2.52 (-7.46, 6.43)	0.55	2020-2023	22.02 (10.37, 41.23)	<0.01
Spain	20.4	73.2	2014-2018	8.31 (-0.54, 13.47)	0.06	2018-2023	22.75 (18.74, 31.22)	<0.01
Sweden	16.2	150.4	2014-2021	37.31 (32.43, 47.61)	<0.01	2021-2023	-9.15 (-26.16, 15.79)	0.52
United Arab Emirates	92.2	617.4	2014-2023	44.63 (34.75, 55.03)	<0.01	–	–	–
United Kingdom	3.1	74.1	2014-2023	11.48 (6.65, 16.33)	<0.01	–	–	–
USA	171.6	387.3	2014-2023	26.36 (21.05, 31.79)	<0.01	–	–	–

Legend: Annual Percentage Change (APC) and 95% Confidence Interval (CI) estimated using joinpoint regression models are shown. Original units and the description of the calculated indicator reported in Supplementary Table 3. The results represent the annual percentage change in the US$ per capita spend in online grocery shopping for each identified segment over the analysis period (2014 to 2023), by country and income groups. Crude metrics for the first (2014) and last (2023) time points are reported. Two-tailed t-test was used to test whether APC was statistically different from zero, with no adjustment made for multiple comparisons. LMICs=Low-and-middle-income countries; UMICs=Upper-and-middle-income countries; HICs=High-income countries.

Supplementary Table 12. Obesity prevalence from 2009 to 2023, by geographic region and country income group: joinpoint regression analysis.

	% prevalence of obesity*		AAPC		Segment 1			Segment 2			Segment 3		
	2009	2022	AAPC (95% CI)	p-value	Years	APC (95% CI)	p-value	Years	APC (95% CI)	p-value	Years	APC (95% CI)	p-value
**Geographic regions**													
Overall (n=97)	18.17%	23.66%	2.05 (2.04, 2.05)	<0.001	2009-2011	2.15 (2.11, 2.19)	<0.001	2011-2017	2.04 (2.03, 2.05)	<0.001	2017-2022	2.01 (1.99, 2.02)	<0.001
East Asia and Pacific (n=14)	8.37%	12.95%	3.41 (3.41, 3.42)	<0.001	2009-2014	3.3 (3.25, 3.32)	<0.001	2014-2019	3.46 (3.38, 3.49)	<0.001	2019-2022	3.54 (3.5, 3.59)	<0.001
South Asia (n=4)	5.35%	11.85%	6.32 (6.3, 6.33)	<0.001	2009-2014	6.68 (6.65, 6.76)	<0.001	2014-2018	6.43 (6.37, 6.5)	<0.001	2018-2022	5.75 (5.68, 5.8)	<0.001
Europe and Central Asia (n=38)	19.47%	23.59%	1.48 (1.48, 1.49)	<0.001	2009-2011	1.58 (1.52, 1.63)	<0.001	2011-2019	1.45 (1.42, 1.46)	<0.001	2019-2022	1.52 (1.47, 1.57)	<0.001
Middle East and North Africa (n=13)	29.49%	35.32%	1.4 (1.39, 1.4)	<0.001	2009-2012	1.62 (1.58, 1.65)	<0.001	2012-2017	1.46 (1.45, 1.48)	<0.001	2017-2022	1.2 (1.19, 1.21)	<0.001
Sub-Saharan Africa (n=10)	9.11%	13.30%	2.93 (2.91, 2.96)	<0.001	2009-2017	2.78 (2.71, 2.83)	<0.001	2017-2022	3.18 (3.1, 3.33)	<0.001	–	–	–
Latin America and Caribbean (n=16)	21.86%	31.77%	2.92 (2.91, 2.93)	<0.001	2009-2013	3.12 (3.1, 3.16)	<0.001	2013-2017	2.95 (2.91, 2.98)	<0.001	2017-2022	2.73 (2.71, 2.75)	<0.001
North America (n=2)	30.23%	34.73%	1.07 (1.07, 1.08)	<0.001	2009-2011	1.37 (1.32, 1.42)	<0.001	2011-2015	1.06 (1.04, 1.08)	<0.001	2015-2022	0.99 (0.97, 1)	<0.001
**Country income groups**													
LMIC (n= 29)	11.3%	16.9%	3.13 (3.12, 3.13)	<0.001	2009-2011	3.28 (3.26, 3.29)	<0.001	2011-2017	3.14 (3.14, 3.15)	<0.001	2017-2022	3.05 (3.04, 3.05)	<0.001
UMIC (n= 26)	20.8%	27.8%	2.26 (2.25, 2.26)	<0.001	2009-2012	2.4 (2.37, 2.44)	<0.001	2012-2016	2.3 (2.26, 2.32)	<0.001	2016-2022	2.16 (2.15, 2.17)	<0.001
HIC (n= 42)	21.3%	25.8%	1.48 (1.47, 1.48)	<0.001	2009-2011	1.58 (1.53, 1.62)	<0.001	2011-2015	1.44 (1.42, 1.45)	<0.001	2015-2022	1.47 (1.46, 1.5)	<0.001

Legend: Annual Average Percentage Change (AAPC), Annual Percentage Change (APC) and 95% Confidence Interval (CI) estimated using joinpoint regression models are shown. The results represent the percentage change in the obesity prevalence for each identified segment and for the entire analysis period (2009 to 2023), by regions and country income groups. *Prevalence of BMI>=30 kg/m² (obesity) among adults (over 18 years old), average of males and females. The sample size (number of countries) is given for each category (first column). Crude metrics for the first (2009) and last (2022) time points are reported. Two-tailed t-test was used to test whether AAPC and APC was statistically different from zero, with no adjustment made for multiple comparisons. LMICs=Low-and-middle-income countries; UMICs=Upper-and-middle-income countries; HICs=High-income countries.

Supplementary Table 13. Obesity prevalence from 2009 to 2023, by country: joinpoint regression analysis.

	Income status	% prevalence of obesity[a]		AAPC		Segment 1			Segment 2			Segment 3		
		2009	2023	AAPC (95% CI)	p-value	Years	APC (95% CI)	p-value	Years	APC (95% CI)	p-value	Years	APC (95% CI)	p-value
**East Asia and Pacific**														
Australia	HIC	25.0%	31.0%	1.61 (1.6, 1.62)	<0.01	2009-2014	1.57 (1.52, 1.59)	<0.01	2014-2022	1.64 (1.62, 1.66)	<0.01	–	–	–
Cambodia	LMIC	2.0%	4.0%	6.98 (6.96, 6.99)	<0.01	2009-2018	7.09 (7.07, 7.11)	<0.01	2018-2022	6.73 (6.68, 6.79)	<0.01	–	–	–
China	UMIC	4.0%	8.0%	6.18 (6.17, 6.19)	<0.01	2009-2017	6.23 (6.21, 6.25)	<0.01	2017-2022	6.1 (6.05, 6.14)	<0.01	–	–	–
Indonesia	LMIC	5.0%	12.0%	6.66 (6.64, 6.68)	<0.01	2009-2015	7 (6.96, 7.04)	<0.01	2015-2019	6.62 (6.56, 6.71)	<0.01	2019-2022	6.04 (5.94, 6.13)	<0.01
Japan	HIC	4.0%	6.0%	3.49 (3.48, 3.51)	<0.01	2009-2014	3.17 (3.13, 3.2)	<0.01	2014-2018	3.54 (3.49, 3.58)	<0.01	2018-2022	3.85 (3.81, 3.92)	<0.01
Laos	LMIC	4.0%	8.0%	5.42 (5.41, 5.43)	<0.01	2009-2018	5.47 (5.45, 5.48)	<0.01	2018-2022	5.31 (5.27, 5.35)	<0.01	–	–	–
Malaysia	UMIC	15.0%	23.0%	3.29 (3.28, 3.3)	<0.01	2009-2011	3.73 (3.65, 3.8)	<0.01	2011-2017	3.32 (3.29, 3.34)	<0.01	2017-2022	3.09 (3.05, 3.12)	<0.01
Myanmar	LMIC	5.0%	8.0%	3.53 (3.52, 3.54)	<0.01	2009-2015	3.43 (3.39, 3.46)	<0.01	2015-2019	3.58 (3.44, 3.62)	<0.01	2019-2022	3.67 (3.62, 3.73)	<0.01
New Zealand	HIC	28.0%	34.0%	1.44 (1.44, 1.45)	<0.01	2009-2011	1.79 (1.75, 1.85)	<0.01	2011-2016	1.45 (1.43, 1.47)	<0.01	2016-2022	1.33 (1.31, 1.34)	<0.01
Philippines	LMIC	5.0%	9.0%	4.25 (4.25, 4.26)	<0.01	2009-2014	4.18 (4.18, 4.19)	<0.01	2014-2022	4.3 (4.29, 4.3)	<0.01	–	–	–
Singapore	HIC	7.0%	14.0%	5.32 (5.31, 5.33)	<0.01	2009-2013	5.06 (5.02, 5.09)	<0.01	2013-2017	5.39 (5.37, 5.43)	<0.01	2017-2022	5.48 (5.46, 5.54)	<0.01
South Korea	HIC	4.0%	7.0%	5.59 (5.57, 5.61)	<0.01	2009-2013	4.84 (4.76, 4.91)	<0.01	2013-2017	5.62 (5.55, 5.71)	<0.01	2017-2022	6.18 (6.13, 6.26)	<0.01
Thailand	UMIC	9.0%	15.0%	4.34 (4.33, 4.35)	<0.01	2009-2011	4.44 (4.39, 4.49)	<0.01	2011-2018	4.37 (4.35, 4.38)	<0.01	2018-2022	4.24 (4.21, 4.27)	<0.01
Vietnam	LMIC	1.0%	2.0%	9.2 (9.18, 9.22)	<0.01	2009-2015	9.62 (9.58, 9.66)	<0.01	2015-2019	9.08 (9.01, 9.17)	<0.01	2019-2022	8.54 (8.44, 8.62)	<0.01
**Europe and Central Asia**														
Austria	HIC	14.0%	16.0%	0.71 (0.69, 0.72)	<0.01	2009-2015	0.5 (0.44, 0.53)	<0.01	2015-2019	0.81 (0.57, 0.9)	<0.01	2019-2022	1 (0.92, 1.12)	<0.01
Azerbaijan	UMIC	20.0%	27.0%	2.37 (2.36, 2.38)	<0.01	2009-2013	2.65 (2.63, 2.68)	<0.01	2013-2017	2.37 (2.34, 2.4)	<0.01	2017-2022	2.14 (2.12, 2.16)	<0.01
Belarus	UMIC	21.0%	22.0%	0.36 (0.35, 0.37)	<0.01	2009-2012	0.68 (0.63, 0.74)	<0.01	2012-2016	0.36 (0.32, 0.4)	<0.01	2016-2022	0.2 (0.17, 0.21)	<0.01
Belgium	HIC	18.0%	20.0%	1.1 (1.09, 1.11)	<0.01	2009-2015	0.97 (0.92, 0.99)	<0.01	2015-2019	1.14 (1.01, 1.21)	<0.01	2019-2022	1.3 (1.23, 1.39)	<0.01
Bosnia and Herzegovina	UMIC	17.0%	22.0%	2.02 (2.02, 2.03)	<0.01	2009-2014	1.89 (1.87, 1.91)	<0.01	2014-2019	2.05 (2.03, 2.07)	<0.01	2019-2022	2.2 (2.17, 2.26)	<0.01
Bulgaria	UMIC	17.0%	21.0%	1.59 (1.59, 1.6)	<0.01	2009-2011	1.82 (1.74, 1.89)	<0.01	2011-2015	1.62 (1.57, 1.64)	<0.01	2015-2022	1.52 (1.5, 1.53)	<0.01
Croatia	HIC	22.0%	32.0%	2.95 (2.95, 2.96)	<0.01	2009-2012	3.17 (3.14, 3.2)	<0.01	2012-2017	2.96 (2.94, 2.98)	<0.01	2017-2022	2.81 (2.79, 2.83)	<0.01
Czech Republic	HIC	22.0%	27.0%	1.67 (1.67, 1.68)	<0.01	2009-2013	1.51 (1.48, 1.54)	<0.01	2013-2022	1.75 (1.74, 1.76)	<0.01	–	–	–
Denmark	HIC	13.0%	14.0%	0.48 (0.46, 0.5)	<0.01	2009-2016	0.34 (0.27, 0.39)	<0.01	2016-2022	0.65 (0.59, 0.74)	<0.01	–	–	–
Estonia	HIC	21.0%	23.0%	0.68 (0.67, 0.69)	<0.01	2009-2011	1.08 (0.98, 1.17)	<0.01	2011-2015	0.69 (0.65, 0.72)	<0.01	2015-2022	0.56 (0.54, 0.58)	<0.01
Finland	HIC	19.0%	22.0%	1 (0.99, 1)	<0.01	2009-2015	0.89 (0.85, 0.9)	<0.01	2015-2019	1.05 (0.91, 1.09)	<0.01	2019-2022	1.15 (1.1, 1.21)	<0.01

France	HIC	13.0%	10.0%	-1.8 (-1.82, -1.78)	<0.01	2009-2012	-1.34 (-1.51, -1.24)	<0.01	2012-2022	-1.94 (-1.97, -1.92)	<0.01	–	–	–
Georgia	UMIC	25.0%	35.0%	2.6 (2.59, 2.61)	<0.01	2009-2013	3 (2.96, 3.05)	<0.01	2013-2017	2.64 (2.59, 2.69)	<0.01	2017-2022	2.25 (2.22, 2.28)	<0.01
Germany	HIC	21.0%	21.0%	0.002 (-0.01, 0.01)	0.62	2009-2011	0.23 (0.12, 0.32)	<0.01	2011-2018	-0.08 (-0.12, -0.06)	<0.01	2018-2022	0.03 (-0.01, 0.12)	0.15
Greece	HIC	24.0%	29.0%	1.34 (1.33, 1.35)	<0.01	2009-2011	1.63 (1.52, 1.7)	<0.01	2011-2020	1.27 (1.24, 1.28)	<0.01	2020-2022	1.35 (1.28, 1.39)	<0.01
Hungary	HIC	24.0%	33.0%	2.36 (2.36, 2.37)	<0.01	2009-2011	2.52 (2.47, 2.55)	<0.01	2011-2017	2.37 (2.36, 2.38)	<0.01	2017-2022	2.29 (2.28, 2.31)	<0.01
Ireland	HIC	25.0%	29.0%	1.29 (1.28, 1.29)	<0.01	2009-2011	1.48 (1.4, 1.53)	<0.01	2011-2022	1.25 (1.25, 1.26)	<0.01	–	–	–
Italy	HIC	16.0%	18.0%	0.7 (0.69, 0.72)	<0.01	2009-2011	0.85 (0.73, 0.97)	<0.01	2011-2017	0.59 (0.53, 0.62)	<0.01	2017-2022	0.77 (0.73, 0.86)	<0.01
Kazakhstan	UMIC	15.0%	19.0%	1.58 (1.57, 1.58)	<0.01	2009-2012	1.55 (1.5, 1.58)	<0.01	2012-2016	1.65 (1.62, 1.66)	<0.01	2016-2022	1.54 (1.53, 1.56)	<0.01
Latvia	HIC	21.0%	25.0%	1.21 (1.2, 1.22)	<0.01	2009-2011	1.49 (1.44, 1.54)	<0.01	2011-2015	1.23 (1.2, 1.25)	<0.01	2015-2022	1.12 (1.1, 1.13)	<0.01
Lithuania	HIC	23.0%	26.0%	1 (0.99, 1.01)	<0.01	2009-2012	1.08 (1.04, 1.13)	<0.01	2012-2022	0.97 (0.96, 0.98)	<0.01	–	–	–
Netherlands	HIC	14.0%	15.0%	0.54 (0.53, 0.56)	<0.01	2009-2011	0.65 (0.54, 0.77)	<0.01	2011-2017	0.43 (0.37, 0.46)	<0.01	2017-2022	0.64 (0.59, 0.72)	<0.01
North Macedonia	UMIC	22.0%	28.0%	2.15 (2.14, 2.15)	<0.01	2009-2015	2.11 (2.09, 2.12)	<0.01	2015-2020	2.17 (2.1, 2.18)	<0.01	2020-2022	2.2 (2.17, 2.22)	<0.01
Norway	HIC	16.0%	20.0%	1.5 (1.49, 1.51)	<0.01	2009-2011	1.72 (1.6, 1.82)	<0.01	2011-2017	1.41 (1.37, 1.44)	<0.01	2017-2022	1.51 (1.48, 1.6)	<0.01
Poland	HIC	22.0%	28.0%	2.16 (2.15, 2.16)	<0.01	2009-2011	2.21 (2.18, 2.25)	<0.01	2011-2017	2.16 (2.14, 2.17)	<0.01	2017-2022	2.13 (2.1, 2.14)	<0.01
Portugal	HIC	18.0%	22.0%	1.54 (1.53, 1.55)	<0.01	2009-2011	1.63 (1.56, 1.71)	<0.01	2011-2018	1.47 (1.44, 1.49)	<0.01	2018-2022	1.62 (1.58, 1.69)	<0.01
Romania	HIC	19.0%	35.0%	4.66 (4.64, 4.68)	<0.01	2009-2013	5.23 (5.16, 5.36)	<0.01	2013-2017	4.75 (4.59, 4.86)	<0.01	2017-2022	4.14 (4.05, 4.2)	<0.01
Russia	UMIC	22.0%	25.0%	0.99 (0.98, 1)	<0.01	2009-2012	1.24 (1.17, 1.3)	<0.01	2012-2016	0.95 (0.91, 1.02)	<0.01	2016-2022	0.89 (0.81, 0.91)	<0.01
Serbia	UMIC	18.0%	23.0%	2.1 (2.09, 2.1)	<0.01	2009-2014	1.96 (1.95, 1.97)	<0.01	2014-2019	2.13 (2.12, 2.15)	<0.01	2019-2022	2.25 (2.23, 2.31)	<0.01
Slovakia	HIC	20.0%	28.0%	2.56 (2.56, 2.57)	<0.01	2009-2011	2.61 (2.59, 2.64)	<0.01	2011-2017	2.57 (2.56, 2.58)	<0.01	2017-2022	2.54 (2.52, 2.54)	<0.01
Slovenia	HIC	16.0%	20.0%	1.49 (1.47, 1.52)	<0.01	2009-2014	0.86 (0.79, 0.92)	<0.01	2014-2018	1.54 (1.44, 1.62)	<0.01	2018-2022	2.25 (2.17, 2.35)	<0.01
Spain	HIC	20.0%	16.0%	-1.69 (-1.72, -1.66)	<0.01	2009-2012	-1.13 (-1.35, -0.99)	<0.01	2012-2022	-1.86 (-1.89, -1.83)	<0.01	–	–	–
Sweden	HIC	15.0%	16.0%	0.39 (0.37, 0.4)	<0.01	2009-2011	0.47 (0.35, 0.59)	<0.01	2011-2016	0.23 (0.17, 0.27)	<0.01	2016-2022	0.49 (0.45, 0.54)	<0.01
Switzerland	HIC	12.0%	12.0%	0.15 (0.13, 0.16)	<0.01	2009-2015	-0.05 (-0.12, -0.01)	0.022	2015-2019	0.24 (0, 0.33)	0.05	2019-2022	0.43 (0.34, 0.56)	<0.01
Turkey	UMIC	29.0%	34.0%	1.23 (1.22, 1.23)	<0.01	2009-2017	1.32 (1.31, 1.32)	<0.01	2017-2022	1.08 (1.07, 1.09)	<0.01	–	–	–
Ukraine	LMIC	21.0%	24.0%	0.93 (0.92, 0.94)	<0.01	2009-2012	1.11 (1.04, 1.16)	<0.01	2012-2022	0.87 (0.86, 0.88)	<0.01	–	–	–
United Kingdom	HIC	25.0%	28.0%	0.87 (0.86, 0.87)	<0.01	2009-2011	1.17 (1.12, 1.22)	<0.01	2011-2022	0.81 (0.8, 0.82)	<0.01	–	–	–
Uzbekistan	LMIC	19.0%	31.0%	3.88 (3.85, 3.9)	<0.01	2009-2013	4.59 (4.5, 4.72)	<0.01	2013-2017	3.94 (3.79, 4.06)	<0.01	2017-2022	3.26 (3.17, 3.33)	<0.01
**Latin America and Caribbean**														
Argentina	UMIC	25.0%	36.0%	2.99 (2.99, 3)	<0.01	2009-2011	3 (2.97, 3.05)	<0.01	2011-2018	3.05 (3.04, 3.07)	<0.01	2018-2022	2.89 (2.87, 2.91)	<0.01
Bolivia	LMIC	19.0%	29.0%	3.43 (3.42, 3.44)	<0.01	2009-2013	3.63 (3.61, 3.67)	<0.01	2013-2017	3.45 (3.4, 3.49)	<0.01	2017-2022	3.25 (3.23, 3.27)	<0.01

Brazil	UMIC	17.0%	29.0%	3.96 (3.95, 3.98)	<0.01	2009-2017	4.11 (4.08, 4.14)	<0.01	2017-2022	3.73 (3.67, 3.8)	<0.01	–	–	–
Chile	HIC	28.0%	40.0%	2.74 (2.73, 2.74)	<0.01	2009-2016	2.84 (2.83, 2.85)	<0.01	2016-2020	2.67 (2.65, 2.7)	<0.01	2020-2022	2.52 (2.48, 2.56)	<0.01
Colombia	UMIC	17.0%	24.0%	2.83 (2.81, 2.84)	<0.01	2009-2012	3.43 (3.36, 3.52)	<0.01	2012-2016	2.96 (2.9, 3.01)	<0.01	2016-2022	2.44 (2.41, 2.47)	<0.01
Costa Rica	UMIC	24.0%	32.0%	2.43 (2.42, 2.44)	<0.01	2009-2012	2.76 (2.72, 2.81)	<0.01	2012-2016	2.5 (2.47, 2.52)	<0.01	2016-2022	2.22 (2.2, 2.24)	<0.01
Dominican Republic	UMIC	21.0%	30.0%	2.83 (2.83, 2.84)	<0.01	2009-2012	3.12 (3.07, 3.16)	<0.01	2012-2017	2.85 (2.82, 2.87)	<0.01	2017-2022	2.65 (2.63, 2.68)	<0.01
Ecuador	UMIC	19.0%	28.0%	3.18 (3.18, 3.18)	<0.01	2009-2013	3.32 (3.31, 3.34)	<0.01	2013-2017	3.17 (3.15, 3.2)	<0.01	2017-2022	3.07 (3.05, 3.08)	<0.01
El Salvador	LMIC	24.0%	31.0%	2.1 (2.1, 2.11)	<0.01	2009-2012	2.41 (2.38, 2.45)	<0.01	2012-2016	2.15 (2.12, 2.17)	<0.01	2016-2022	1.92 (1.9, 1.93)	<0.01
Guatemala	UMIC	18.0%	28.0%	3.1 (3.09, 3.11)	<0.01	2009-2013	3.46 (3.43, 3.51)	<0.01	2013-2017	3.1 (3.05, 3.14)	<0.01	2017-2022	2.81 (2.77, 2.84)	<0.01
Honduras	LMIC	21.0%	30.0%	2.77 (2.75, 2.78)	<0.01	2009-2013	3.17 (3.13, 3.22)	<0.01	2013-2017	2.75 (2.7, 2.8)	<0.01	2017-2022	2.46 (2.41, 2.49)	<0.01
Mexico	UMIC	28.0%	37.0%	2.13 (2.12, 2.13)	<0.01	2009-2013	2.38 (2.36, 2.41)	<0.01	2013-2017	2.13 (2.1, 2.15)	<0.01	2017-2022	1.92 (1.9, 1.94)	<0.01
Panama	HIC	25.0%	37.0%	3.14 (3.13, 3.16)	<0.01	2009-2013	3.48 (3.44, 3.55)	<0.01	2013-2017	3.19 (3.1, 3.25)	<0.01	2017-2022	2.83 (2.79, 2.87)	<0.01
Paraguay	UMIC	24.0%	34.0%	2.85 (2.85, 2.86)	<0.01	2009-2016	2.94 (2.93, 2.94)	<0.01	2016-2020	2.8 (2.79, 2.81)	<0.01	2020-2022	2.68 (2.65, 2.71)	<0.01
Peru	UMIC	17.0%	28.0%	3.94 (3.93, 3.95)	<0.01	2009-2018	4 (3.99, 4.02)	<0.01	2018-2022	3.79 (3.73, 3.84)	<0.01	–	–	–
Uruguay	HIC	24.0%	34.0%	2.92 (2.92, 2.93)	<0.01	2009-2017	2.98 (2.97, 2.99)	<0.01	2017-2022	2.83 (2.81, 2.84)	<0.01	–	–	–
**Middle East and North Africa**														
Algeria	LMIC	18.0%	25.0%	2.44 (2.43, 2.44)	<0.01	2009-2017	2.57 (2.56, 2.58)	<0.01	2017-2022	2.22 (2.21, 2.24)	<0.01	–	–	–
Egypt	LMIC	36.0%	46.0%	1.78 (1.78, 1.79)	<0.01	2009-2013	2.07 (2.05, 2.12)	<0.01	2013-2017	1.86 (1.83, 1.89)	<0.01	2017-2022	1.5 (1.47, 1.52)	<0.01
Iraq	UMIC	33.0%	41.0%	1.75 (1.74, 1.76)	<0.01	2009-2012	2.01 (1.96, 2.08)	<0.01	2012-2017	1.84 (1.82, 1.87)	<0.01	2017-2022	1.5 (1.48, 1.52)	<0.01
Israel	HIC	22.0%	23.0%	0.29 (0.28, 0.3)	<0.01	2009-2011	0.53 (0.4, 0.64)	<0.01	2011-2018	0.19 (0.14, 0.21)	<0.01	2018-2022	0.34 (0.29, 0.47)	<0.01
Jordan	UMIC	37.0%	40.0%	0.68 (0.67, 0.7)	<0.01	2009-2012	1.02 (0.96, 1.13)	<0.01	2012-2016	0.78 (0.67, 0.84)	<0.01	2016-2022	0.45 (0.41, 0.47)	<0.01
Kuwait	HIC	42.0%	44.0%	0.36 (0.36, 0.37)	<0.01	2009-2012	0.5 (0.47, 0.53)	<0.01	2012-2017	0.42 (0.41, 0.43)	<0.01	2017-2022	0.22 (0.21, 0.23)	<0.01
Lebanon	LMIC	26.0%	31.0%	1.38 (1.37, 1.39)	<0.01	2009-2012	1.66 (1.6, 1.72)	<0.01	2012-2017	1.44 (1.41, 1.47)	<0.01	2017-2022	1.16 (1.13, 1.18)	<0.01
Morocco	LMIC	16.0%	23.0%	2.79 (2.78, 2.8)	<0.01	2009-2012	3.03 (3, 3.11)	<0.01	2012-2017	2.93 (2.9, 2.95)	<0.01	2017-2022	2.5 (2.48, 2.52)	<0.01
Oman	HIC	26.0%	34.0%	2.18 (2.17, 2.19)	<0.01	2009-2012	2.39 (2.36, 2.45)	<0.01	2012-2017	2.3 (2.28, 2.32)	<0.01	2017-2022	1.93 (1.91, 1.95)	<0.01
Qatar	HIC	37.0%	47.0%	1.76 (1.75, 1.77)	<0.01	2009-2012	2.11 (2.04, 2.16)	<0.01	2012-2017	1.84 (1.81, 1.87)	<0.01	2017-2022	1.46 (1.44, 1.49)	<0.01
Saudi Arabia	HIC	35.0%	43.0%	1.53 (1.52, 1.54)	<0.01	2009-2012	1.81 (1.76, 1.85)	<0.01	2012-2017	1.61 (1.58, 1.63)	<0.01	2017-2022	1.28 (1.26, 1.3)	<0.01
Tunisia	LMIC	21.0%	28.0%	2.01 (2.01, 2.02)	<0.01	2009-2012	2.18 (2.16, 2.23)	<0.01	2012-2017	2.1 (2.09, 2.12)	<0.01	2017-2022	1.81 (1.8, 1.83)	<0.01
United Arab Emirates	HIC	33.0%	35.0%	0.35 (0.34, 0.37)	<0.01	2009-2012	0.58 (0.52, 0.65)	<0.01	2012-2017	0.4 (0.35, 0.42)	<0.01	2017-2022	0.18 (0.15, 0.21)	<0.01
**North America**														
Canada	HIC	25.0%	27.0%	0.65 (0.64, 0.66)	<0.01	2009-2011	0.96 (0.85, 1.02)	<0.01	2011-2022	0.6 (0.59, 0.6)	<0.01	–	–	–

USA	HIC	36.0%	43.0%	1.35 (1.34, 1.36)	<0.01	2009-2011	1.68 (1.63, 1.73)	<0.01	2011-2016	1.35 (1.33, 1.37)	<0.01	2016-2022	1.24 (1.22, 1.26)	<0.01
**South Asia**														
Bangladesh	LMIC	2.0%	5.0%	7.87 (7.84, 7.9)	<0.01	2009-2013	8.64 (8.51, 8.8)	<0.01	2013-2018	7.94 (7.82, 8.04)	<0.01	2018-2022	7.01 (6.89, 7.14)	<0.01
India	LMIC	4.0%	8.0%	5.9 (5.89, 5.91)	<0.01	2009-2011	6.24 (6.12, 6.34)	<0.01	2011-2018	6.02 (5.99, 6.04)	<0.01	2018-2022	5.51 (5.47, 5.56)	<0.01
Pakistan	LMIC	11.0%	24.0%	6.09 (6.07, 6.11)	<0.01	2009-2016	6.48 (6.45, 6.52)	<0.01	2016-2020	5.85 (5.78, 5.96)	<0.01	2020-2022	5.19 (5.03, 5.34)	<0.01
Sri Lanka	LMIC	5.0%	11.0%	6.41 (6.39, 6.43)	<0.01	2009-2013	6.76 (6.69, 6.86)	<0.01	2013-2018	6.5 (6.43, 6.55)	<0.01	2018-2022	5.95 (5.89, 6.01)	<0.01
**Sub-Saharan Africa**														
Angola	LMIC	8.0%	12.0%	2.99 (2.96, 3.01)	<0.01	2009-2017	2.87 (2.81, 2.91)	<0.01	2017-2022	3.18 (3.09, 3.34)	<0.01	–	–	–
Cameroon	LMIC	11.0%	15.0%	2.42 (2.41, 2.45)	<0.01	2009-2011	2.74 (2.54, 2.9)	<0.01	2011-2017	2.21 (2.14, 2.24)	<0.01	2017-2022	2.56 (2.5, 2.65)	<0.01
Côte d'Ivoire	LMIC	8.0%	12.0%	3.43 (3.39, 3.47)	<0.01	2009-2016	3.12 (2.98, 3.21)	<0.01	2016-2022	3.79 (3.67, 3.97)	<0.01	–	–	–
Ethiopia	LMIC	1.0%	3.0%	6.63 (6.62, 6.64)	<0.01	2009-2014	6.42 (6.39, 6.44)	<0.01	2014-2018	6.66 (6.62, 6.69)	<0.01	2018-2022	6.87 (6.84, 6.91)	<0.01
Ghana	LMIC	10.0%	13.0%	2.4 (2.39, 2.42)	<0.01	2009-2011	2.6 (2.43, 2.74)	<0.01	2011-2016	2.16 (2.08, 2.21)	<0.01	2016-2022	2.55 (2.5, 2.6)	<0.01
Kenya	LMIC	7.0%	13.0%	4.44 (4.43, 4.45)	<0.01	2009-2012	4.74 (4.7, 4.78)	<0.01	2012-2017	4.42 (4.4, 4.46)	<0.01	2017-2022	4.27 (4.24, 4.3)	<0.01
Nigeria	LMIC	8.0%	13.0%	3.56 (3.52, 3.59)	<0.01	2009-2016	3.3 (3.2, 3.38)	<0.01	2016-2022	3.85 (3.76, 3.99)	<0.01	–	–	–
South Africa	UMIC	28.0%	31.0%	0.9 (0.89, 0.91)	<0.01	2009-2011	1.2 (1.13, 1.27)	<0.01	2011-2018	0.81 (0.78, 0.82)	<0.01	2018-2022	0.91 (0.88, 0.99)	<0.01
Tanzania	LMIC	7.0%	13.0%	5.3 (5.29, 5.31)	<0.01	2009-2011	5.56 (5.52, 5.6)	<0.01	2011-2017	5.28 (5.27, 5.29)	<0.01	2017-2022	5.22 (5.2, 5.24)	<0.01
Uganda	LMIC	3.0%	8.0%	6.6 (6.58, 6.61)	<0.01	2009-2014	6.16 (6.07, 6.21)	<0.01	2014-2018	6.66 (6.49, 6.81)	<0.01	2018-2022	7.09 (7.02, 7.21)	<0.01

Legend: Annual Average Percentage Change (AAPC), Annual Percentage Change (APC) and 95% Confidence Interval (CI) estimated using joinpoint regression models are shown. The results represent the obesity prevalence for each identified segment and for the entire analysis period (2009 to 2022), by country. *Prevalence of BMI>=30 kg/m² (obesity) among adults (over 18 years old), average of males and females. Crude metrics for the first (2009) and last (2022) time points are reported. Two-tailed t-test was used to test whether AAPC and APC was statistically different from zero, with no adjustment made for multiple comparisons. LMICs=Low-and-middle-income countries; UMICs=Upper-and-middle-income countries; HICs=High-income countries.
